# Deep-learning single-cell profiling uncovers interbacterial and drug-driven reshaping of pathogen virulence

**DOI:** 10.1038/s41467-026-77989-w

**Published:** 2026-09-29

**Authors:** Bence Bognár, Terézia Kovács, Réka Muresan, Ede Migh, Tímea Balogh, Nándor Milán Kovács, Áron Somogyi, Árpád Molnár, Bálint Csörgő, Csaba Pál, Péter Horváth, Szilvia Juhász, Viktória Lázár

**Affiliations:** 1 Synthetic and Systems Biology Unit, Institute of Biochemistry, HUN-REN Biological Research Centre, Szeged, Hungary; 2 HCEMM-BRC Pharmacodynamic Drug Interaction Research Group, Szeged, Hungary; 3 Doctoral School of Biology, University of Szeged, Szeged, Hungary; 4 HCEMM Cancer Microbiome Core Group, Szeged, Hungary; 5 Single-Cell Technologies Ltd., Szeged, Hungary; 6 Institute of AI for Health, Helmholtz Zentrum München, Neuherberg, Germany; 7 Institute for Molecular Medicine Finland, University of Helsinki, Helsinki, Finland

**Keywords:** Antibiotics, Phenotypic screening, Cellular microbiology

## Abstract

Bacterial invasion and genotoxicity are key contributors to infectious disease progression and carcinogenesis. Yet current methods do not quantify these processes systematically and at single-cell resolution, limiting our ability to explore how interplay of virulence factors and drug effects shape pathogenicity. Here, we present MALVINA, a high-throughput deep-learning workflow integrating host-pathogen co-culture and high-content imaging to jointly measure bacterial invasion and host DNA damage. Applied to *Escherichia coli* isolates and human colorectal epithelial cells, we demonstrate MALVINA’s versatility by deciphering (1) strains with elevated invasive or genotoxic potential, (2) interbacterial modulation of virulence, (3) pro-virulent drug effects, (4) virulence-targeting drugs, and (5) bacterial potentiation of drug toxicity. MALVINA revealed strain-specific virulence profiles and colibactin-dependent suppression of competitors’ invasion coupled with enhanced genotoxicity. It also enabled identification of drug-induced modulation of bacterial invasion and genotoxicity, offering a new perspective for virulence-aware drug profiling. Overall, MALVINA offers an adaptable framework for dissecting pathogen-host-drug interactions.

## Abstract (web summary)

This study introduces MALVINA, a deep-learning platform that measures bacterial invasion and DNA damage in individual human cells, revealing how bacterial interactions and commonly used drugs reshape pathogen virulence.

## Introduction

Accurate identification and characterisation of highly virulent bacterial strains are crucial for uncovering novel pathogenic mechanisms, elucidating host-pathogen interactions, informing public health, and enabling early disease detection. Bacterial pathogenesis arises from virulence factor-mediated interactions that drive tissue damage, inflammatory responses, and clinical manifestations of disease. Two complementary virulence traits are central. Bacterial invasion enables penetration of host barriers, intracellular replication, immune evasion, and persistence within the host^1–3^. These processes include adhesins, secretion systems that deliver virulence effectors, and bacterial factors promoting epithelial attachment, uptake, and intracellular persistence. Genotoxicity may arise from bacterial genotoxins; among these, colibactin produced by polyketide synthetase (pks) island-harbouring Enterobacteriaceae has emerged as a major driver of host DNA double-strand breaks and genomic instability^4^. Additional virulence-associated mechanisms, including cytolethal distending toxins and infection-induced oxidative stress, can further contribute to host-cell DNA damage^5^. Emerging evidence links adherent-invasive and genotoxic gut bacteria to severe gastrointestinal diseases, including gastritis, inflammatory bowel disease, and gastric or colorectal cancer^6–8^. Despite mounting evidence implicating gut bacteria in these diseases, several factors influencing bacterial virulence remain poorly understood.

Most studies of bacterial virulence focus on single strains, despite growing evidence that bacteria can modulate each other’s pathogenicity. Bacterial interactions are typically classified by fitness outcomes, overlooking the modulation of virulence factors through social dynamics^9^. Microbial communities shape pathogenicity through interbacterial interactions that can be cooperative or competitive, driven by mechanisms evolved to detect and respond to signals from neighbouring bacteria^10,11^. Cooperative sharing of exoenzymes, siderophores, biosurfactants, or quorum-sensing signals can enhance virulence, whereas resource competition, cheating, or toxin production can suppress it^12–16^. Understanding inter- and intraspecies virulence interactions could guide the design of targeted probiotics and novel strategies to mitigate pathogenic behaviour within complex microbial communities.

While pharmaceutical research has traditionally focused on pathogen killing, growth inhibition, and therapeutic safety, much less is known about how drugs influence bacterial virulence or how bacteria modulate drug-induced toxicity^17–22^. Consequently, the impact of sub-therapeutic or off-target drug effects on virulence processes, such as intracellular replication, tissue dispersion, and genotoxicity, remains largely unexplored^23^. Even subtle drug-induced changes in pathogenic behaviour can alter disease severity, treatment outcomes, and resistance emergence^18^. Emerging antimicrobial strategies targeting infection-related functions rather than viability alone may broaden therapeutic options and help protect the host microbiome^17,18^. Moreover, bacteria themselves can modulate drugs within host cells, affecting their bioactivity or toxicity^24,25^. Systematically elucidating these drug-pathogen-host interactions can inform the design of safer, more effective treatments for patients.

Current methods for evaluating bacterial invasion in human cell lines include antibiotic protection assays, fluorescence microscopy, flow cytometry, and automated high-content imaging approaches. Antibiotic protection assays use gentamicin to eliminate extracellular bacteria, followed by host cell lysis and quantification of internalised bacteria through colony-forming unit counts^26,27^. While simple and widely used, these assays provide only population-level data, obscuring single-cell heterogeneity and failing to distinguish actively replicating intracellular bacteria from those that persist or spread across cell layers. Fluorescence microscopy and flow cytometry enable single-cell analysis of intracellular bacteria but remain limited by low throughput, semi-quantitative readouts, or artefacts arising from biofilm aggregates and non-specific adherence^28–35^. More recently, high-content microscopy and machine learning–assisted image analysis have been applied to quantify intracellular infection and host-pathogen interaction phenotypes^36^.

Genotoxicity is typically assessed separately, using immunofluorescence detection of phosphorylated histone γH2AX as a marker of DNA double-strand breaks^37^. Cells infected with genotoxic *Escherichia coli* strains carrying the polyketide synthetase (pks) island, which encodes colibactin, induce γH2AX foci detectable by confocal microscopy or by high-throughput flow cytometry^38–42^. Elevated γH2AX levels have also been reported in *Staphylococcus aureus*–infected cells compared to uninfected control experiments, indicating bacteria-induced host-stress-related DNA damage^5^. However, bacterial invasion and host DNA damage are typically measured in separate assays, precluding direct correlation of bacterial burden and host genotoxicity at the single-cell level. To date, no existing approach integrates both of these readouts into a unified, high-throughput, single-cell-resolution assay.

Machine learning has become central to imaging-based human cell analysis, improving classification accuracy and reducing artefacts in microscopy data. Open-source and commercial deep learning tools enable supervised classification by training models on user-annotated cells, providing scalable and consistent image analysis with higher throughput^43–46^. These accessible platforms have advanced nuclear segmentation in dense or poorly stained samples and automated quantification of DNA double-strand breaks via γH2AX foci detection^47–49^. They also capture subtle morphological differences to classify tumour subtypes and cellular phenotypes^50,51^. Despite these advances, current host-pathogen imaging workflows do not integrate bacterial invasion and host-cell DNA damage within a unified single-cell framework.

To address these methodological gaps, we developed MALVINA (Machine Learning–Based Virulence Interaction Analysis), a broadly applicable framework integrating high-content microscopy with deep learning–based cell classification to quantify bacterial invasion and host-cell DNA damage at single-cell resolution. MALVINA enables the extraction of multiparametric virulence scores from a unified high-throughput assay, allowing direct correlation of intracellular bacterial burden with carcinogenesis-relevant host responses. This versatile workflow enables systematic analysis of how strain properties, interbacterial interactions, and drug exposure modulate host-pathogen dynamics.

## Results

### MALVINA overview

**Fig. 1:**
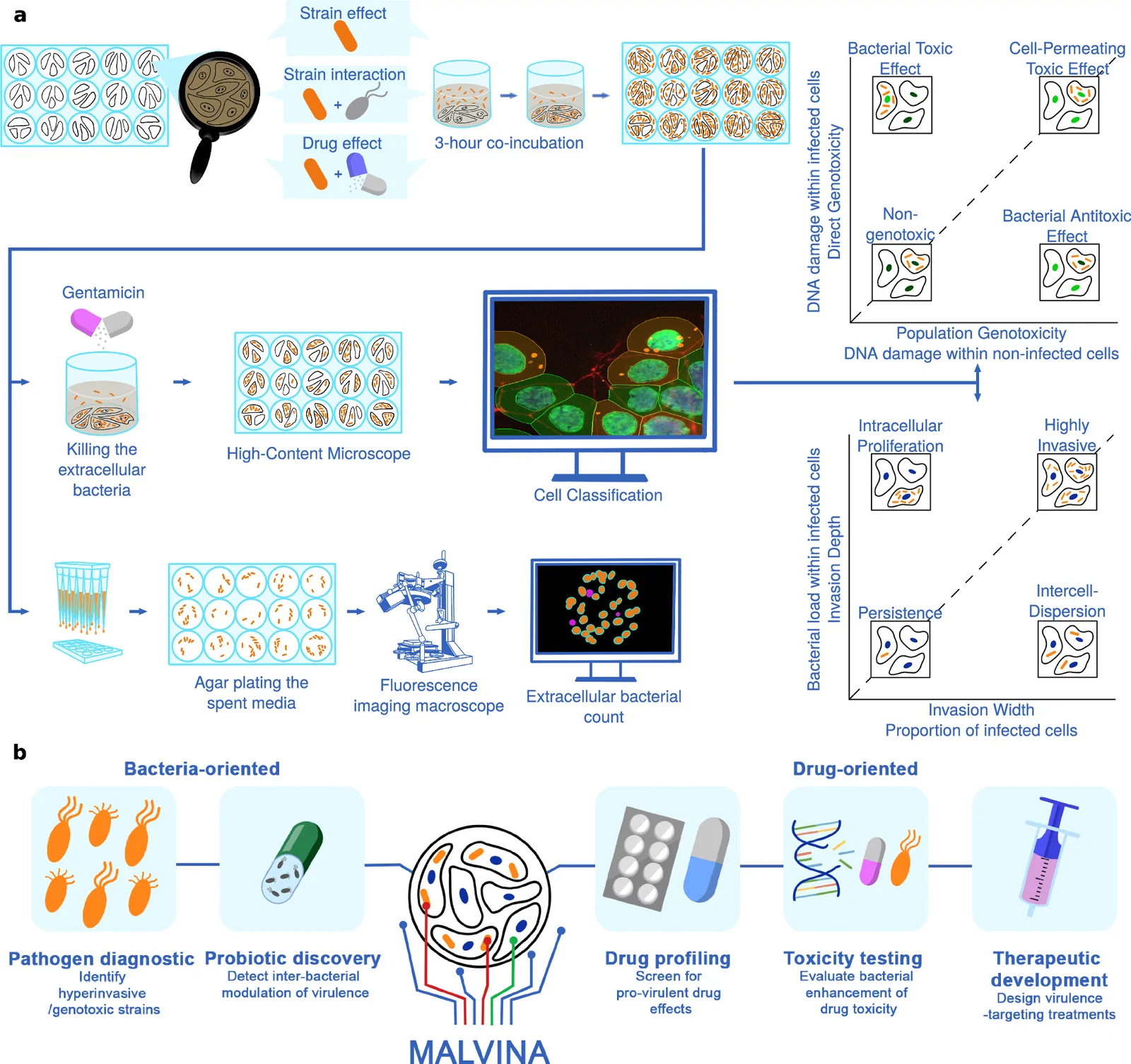
Schematic of the MALVINA workflow. **a** Human colorectal epithelial cells are seeded into 96-well plates, allowed to form monolayers, and infected for 3 h under one of three conditions: (i) a single fluorescently labelled strain, (ii) co-infection with labelled and unlabelled strains, or (iii) a labelled strain exposed to various drugs. After incubation, the workflow splits into two branches. Bottom branch: supernatant is collected, diluted, plated, and colonies counted to quantify extracellular bacteria—key for the drug screen. Top branch: monolayers are treated with gentamicin for 6 h, fixed, stained for γH2AX (DNA damage, green), ZO-1 (tight junctions, red), and nuclei (blue), then imaged. Automated segmentation and machine learning classify infected (cells with orange bacterial signal) and uninfected cells and extract four single-cell metrics: invasion width (proportion of infected cells), invasion depth (intracellular bacterial load within infected cells), population genotoxicity (DNA damage in noninfected cells) and direct genotoxicity (DNA damage in infected cells). **b** Potential use-cases of the MALVINA workflow.

To systematically quantify pathogen invasion, genotoxicity, and their interplay under diverse conditions, we developed a high-throughput workflow (Fig. 1). The system integrates multi-well pathogen-host cell co-culturing, immunofluorescence staining, high-content imaging, and deep learning–based classification. Briefly, human colorectal epithelial cells are seeded into 96-well plates, allowed to form confluent monolayers, and infected with fluorescently labelled bacteria, alone or in combination with unlabelled partner strains or test compounds. After a 3-h co-incubation, culture supernatant is collected to assess drug-induced bacterial killing by colony-forming unit counts. The remaining monolayer is treated with gentamicin to remove extracellular bacteria, allowing intracellular replication and associated genotoxic effects to develop over a 6-h period. Cells are then fixed and immunostained to label cell boundaries (ZO-1), DNA double-strand breaks (γH2AX), and nuclei (Hoechst 33342), followed by high-content imaging (Perkin-Elmer Operetta) capturing ~1000 host cells per well.

For automated image analysis, we developed a custom pipeline within the Biological Image Analysis Software (BIAS) platform^52^. Nuclei were segmented using the built-in module^53^, and successive dilations approximated full cell boundaries (“Methods”, Supplementary Fig. 1). A multilayer perceptron model trained on 2373 manually annotated cells classified infected versus uninfected cells with 86% accuracy. The pipeline also filtered out artefacts such as out-of-focus cells and extracellular bacterial aggregates, yielding clean, single-cell datasets (Supplementary Fig. 1). Automated single-cell classification enables quantification of (1) invasion width, defined as the proportion of infected cells within the total cell population, and (2) invasion depth, measured as the average integrated bacterial signal in infected cells. Genotoxicity is likewise evaluated along two dimensions: (1) population-level genotoxicity, based on the average integrated genotoxic signal in uninfected cells, and (2) direct genotoxicity, derived from the average integrated genotoxic signal in infected cells. These high-resolution readouts distinguish actively replicating intracellular bacteria from those persisting with limited replication and reveal differences in their extent of dispersal across the host cell layer. Concurrently, the refined genotoxicity measure separates host DNA damage triggered by intracellular bacterial stress response from that induced by secreted toxins. This integrative, multiparametric approach reveals complex pathogen-host interactions and enables detailed characterisation of bacterial virulence under microbial or pharmacological modulation. Using MALVINA, we analysed 1320 microwells, 85,800 imaging fields, and 1,300,000 human colorectal epithelial host cells to (1) profile the virulence of four *Escherichia coli* isolates, (2) map inter-strain virulence modulation, and (3) screen 23 drugs for their effects on two invasive clinical isolates^7,54–56^.

### Profiling pathogen invasiveness and genotoxicity

To demonstrate that our multiparametric single-cell approach enables accurate characterisation of bacterial pathogenicity, we evaluated the invasion and genotoxicity profiles of four *E. coli* isolates in a human colorectal epithelial (Caco-2) infection model^57^. The panel included a highly virulent adherent-invasive clinical strain (LF82)^54,55^, two colibactin-producing clinical isolates (CCR20^7,56^ and ATCC25922^58^, hereafter referred to as ATCC), and a non-pathogenic laboratory strain (BW25113^59,60^, hereafter referred to as BW). All strains were transformed with an mScarlet plasmid, yielding comparable fluorescence intensities with minimal variation (maximum signal ratio = 1.42; Supplementary Fig. 2a). The Caco-2 cell line was chosen for its ability to form polarised epithelial monolayers with tight junctions and microvilli, resembling the human intestinal barrier, the primary site of *E. coli* colonisation and invasion^57^. Its uniform growth in 96-well plates provides a reliable, scalable model for high-throughput co-infection assays across multiple bacterial strains^38,61^.

**Fig. 2:**
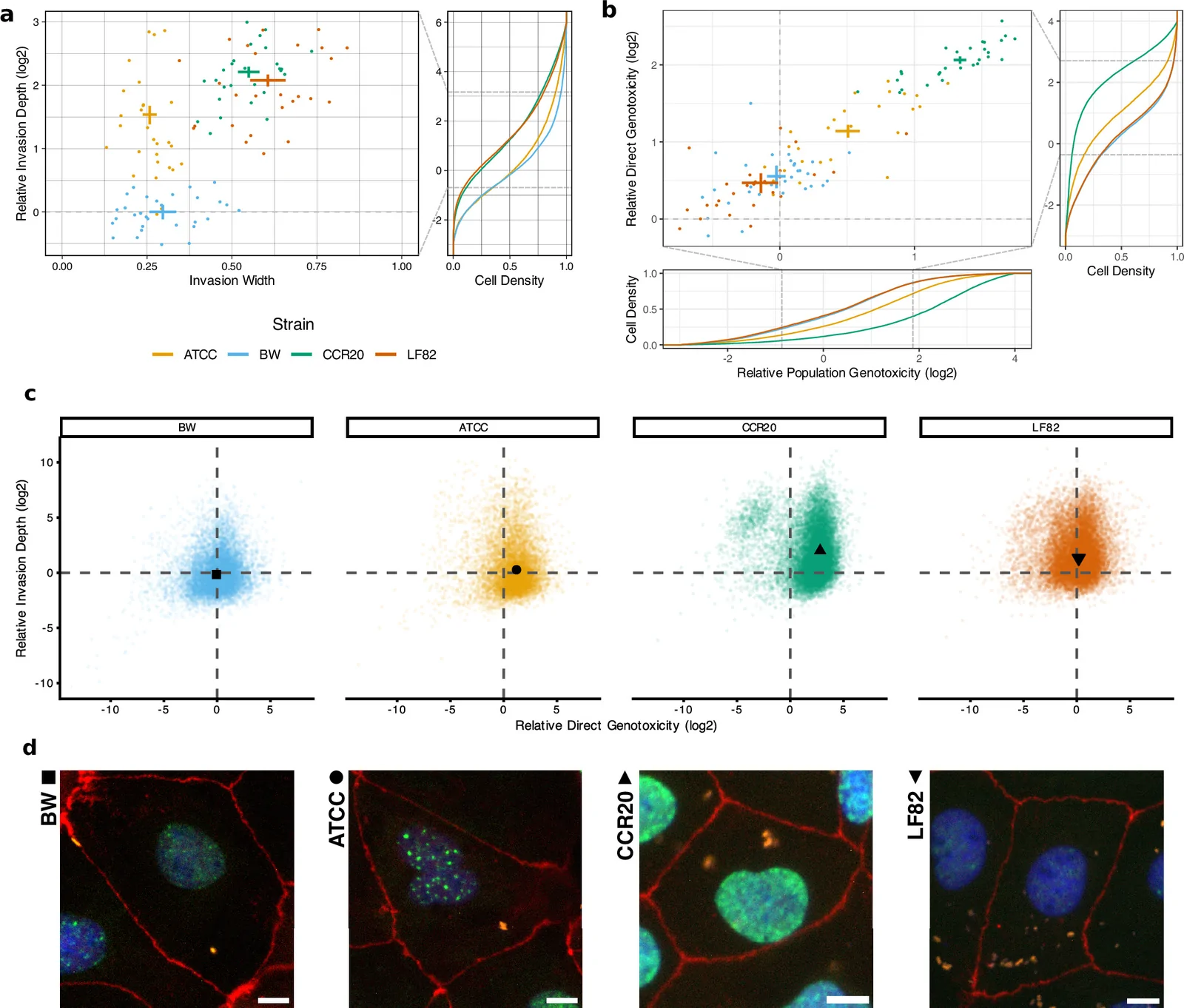
Single-cell quantification of bacterial invasion and genotoxicity reveals strain-specific virulence profiles. **a**, **b** Invasion (**a**) and genotoxicity (**b**) profiles of four *E. coli* strains. Invasion depth was calculated relative to the BW strain, while direct and population genotoxicity relative to uninfected controls. Each point is a replicate measurement; crosses show the mean ± confidence interval. Geometrically normalised cumulative density plots show single-cell distributions for each strain across the different single-cell–derived metrics. Data were derived from three biological replicates, each with at least ten technical replicates. **c** Comparative scatter plot of direct genotoxicity versus invasion depth for single cells infected with four *E. coli* strains, shown relative to the BW strain: BW (blue), ATCC (orange), CCR20 (green), and LF82 (red). Each point represents a single cell measurement aggregated across replicates. Notably, a small subpopulation of, e.g., CCR20-containing cells displayed low genotoxicity signal, indicating heterogeneous induction of DNA damage signalling. **d** Representative single-cell images of BW (square), ATCC (circle), CCR20 (downward-pointing triangle), and LF82 (upward-pointing triangle), as indicated in Fig. 2c. Staining corresponds to host nuclei (Hoechst 33342, blue), the host DNA damage marker γH2AX (green), the tight junction protein ZO-1 (red), and mScarlet-labelled bacteria (orange). Scale bar: 10 µm. Data were derived from three biological replicates, each with at least ten technical replicates.

Invasion profiling revealed marked strain-specific differences in epithelial invasion behaviour (Fig. 2a, c, d and Supplementary Fig. 2c–f). LF82 and CCR20 displayed similarly high invasion width and depth, consistent with strongly invasive phenotypes characterised by broad epithelial dissemination and intracellular accumulation. This aligns with the established adherent-invasive *E. coli* (AIEC) phenotype of LF82 and its association with inflammatory bowel disease^54,62,63^. Hereafter, LF82 and CCR20 are referred to as “hyper-invasive” strains due to their simultaneously high invasion width and invasion depth. In contrast, ATCC showed lower invasion width but relatively high invasion depth, suggesting limited spread across the host cell layer despite substantial intracellular burden. As expected, BW displayed minimal invasion and dispersal, serving as a non-pathogenic reference strain. To provide genetic context for these phenotypes, we annotated virulence-associated loci in the genomes of the four *E. coli* strains (Supplementary Fig. 2g). Compared to the minimally invasive BW strain, the invasive clinical isolates encoded expanded host-adaptation and virulence repertoires, including Chu/Shu heme uptake systems, yersiniabactin-associated iron acquisition loci, the epithelial adhesion factor FdeC, the autotransporter toxin Vat, and K1 capsule-associated loci. Notably, the two most widely disseminating strains, CCR20 and LF82, both encoded T6SS-associated loci absent from the ATCC strain, consistent with a potential contribution of secretion-associated host interaction mechanisms to epithelial invasion.

Assessment of DNA damage further distinguished strain-specific genotoxicity profiles (Fig. 2b). BW and the highly invasive LF82 showed the lowest genotoxicity, consistent with their lack of genotoxin production, yet both induced higher direct genotoxicity than uninfected controls, likely reflecting host stress responses to intracellular bacteria (Fig. 2b–d and Supplementary Fig. 2c, e, g). In contrast, the clinical isolates CCR20 and ATCC, both carrying the colibactin-producing pks island, exhibited elevated genotoxicity profiles consistent with colibactin-mediated DNA damage (Fig. 2b–d and Supplementary Fig. 2d, f, g). The ATCC isolate showed a milder genotoxic profile, suggesting variability in colibactin production even at similar bacterial loads. The combined high invasiveness and genotoxicity of CCR20 highlights its multifactorial invasive and tumorigenic potential (Fig. 2b–d and Supplementary Fig. 2d)^7,56^. These findings indicate that integrated invasion and genotoxicity profiling captures functionally distinct pathogenic phenotypes. Combined with comparative genetic analysis, this approach enables interpretation of how distinct virulence gene repertoires relate to invasion and genotoxicity phenotypes.

To test whether MALVINA can be adapted beyond the Caco-2/*E. coli* infection model, we next applied the workflow to an independent infection system using human lung epithelial A549 adenocarcinoma cells and five clinical *Klebsiella pneumoniae* isolates. Because of the distinct morphology and staining characteristics of A549 cells, we trained a new infection classification model for this host-cell background (“Methods”). Despite the broadly similar virulence gene repertoires of the *K. pneumoniae* isolates, our approach resolved moderate strain-specific differences in invasion width and depth (Supplementary Fig. 3). Several isolates also induced modest strain-specific increases in direct DNA damage signal, whereas population-level DNA damage remained limited, consistent with the absence of known genotoxin production in these strains. Together, these results further demonstrate the adaptability of MALVINA across distinct host-pathogen contexts in identifying high-risk virulent strains.

### Mapping interbacterial modulation of virulence

**Fig. 3:**
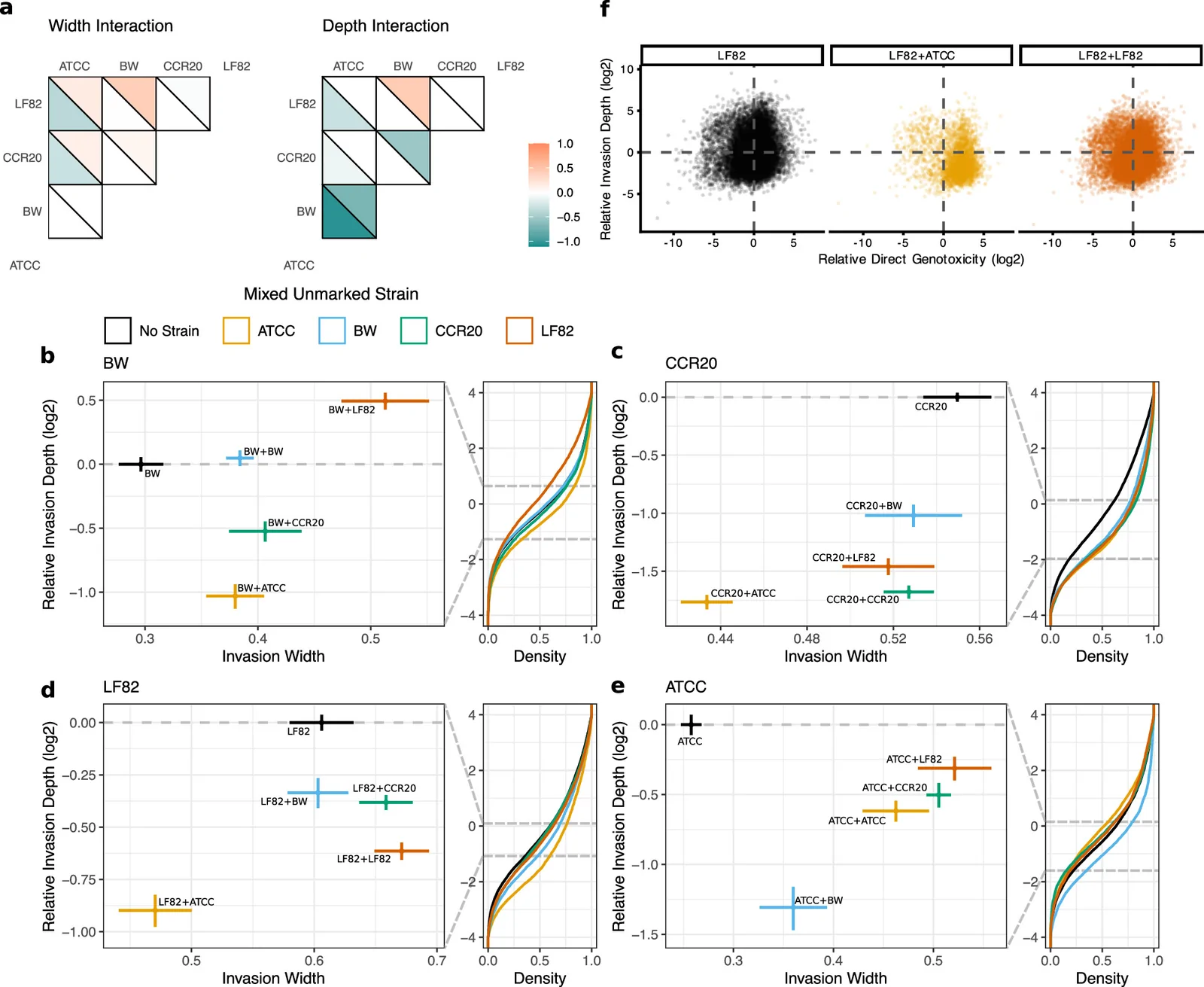
Mapping inter-strain modulation of bacterial invasion. **a** Directional interaction scores for the two key invasion traits, invasion width and invasion depth, across all pairwise combinations of the four *E. coli* strains. The top (bottom) triangles show the interaction score for the effect of the bacteria in the row (column) on the bacteria in the column (row). Orange indicates enhancement, cyan indicates suppression. **b**–**e** Invasion modulation landscapes for fluorescently labelled BW (**b**), CCR20 (**c**), LF82 (**d**), and ATCC (**e**) in the presence of the various unlabelled partner strains. Invasion depth is calculated relative to the corresponding fluorescently labelled single-strain control, indicated by the black cross in each plot. Geometrically normalised cumulative density plots show single-cell distributions of the relative invasion depth of the labelled strain, either alone (black) or mixed with various unlabelled strains (ATCC, orange; BW, blue; CCR20, green; LF82, red). **f** Comparative scatter plot of direct genotoxicity versus invasion depth for single cells identified as labelled LF82-infected, either infected alone (black) or co-infected with unlabelled ATCC (orange) or LF82 (red), shown relative to the mean of the labelled LF82 control. Each point represents a single cell measurement aggregated across replicates. Data were derived from three biological replicates, each with at least three technical replicates (Supplementary Data 1). Errors represent s.e.m.

To investigate how *E. coli* strains influence one another’s invasive phenotype, we applied a co-infection assay using fluorescently labelled and unlabelled variants and quantified the labelled strain’s invasiveness in each pairing (“Methods”, Fig. 1). Systematic analysis of all pairwise combinations generated directional interaction scores for invasion width and depth (Fig. 3a). Scores were calculated relative to two baselines: (1) a single-strain control, where the labelled strain was cultured alone at half the total bacterial load used in the infection mix, and (2) a self-mix control, where labelled and unlabelled cells of the same strain were combined at equal ratios. For each invasion trait, decreases were measured relative to the lower baseline and increases relative to the higher one, ensuring only deviations beyond intra-strain variability were classified as significant suppressive or enhancing effects. This scoring approach was applied to both virulence metrics, enabling robust detection of strain interactions that modulate these traits (“Methods”).

The typically non-invasive laboratory strain BW showed increased invasion width during co-infection with hyper-invasive strains LF82 and CCR20, most prominently with LF82, which also enhanced BW’s invasion depth (Fig. 3a, b). These results suggest cooperative invasion or bystander uptake, where invasive strains facilitate entry of otherwise non-invasive bacteria, potentially by altering host entry pathways or disrupting epithelial barriers^64^. In contrast, co-infection with ATCC or CCR20 reduced BW’s invasion depth, indicating intracellular competition (Fig. 3a, b). Notably, ATCC-BW co-infection revealed mutual antagonism, with both strains showing decreased invasion depth, suggesting competition for intracellular niches (Fig. 3a, b, e). Interestingly, the genotoxic ATCC isolate exhibited unidirectional modulation during co-infection with LF82 and CCR20, showing modestly increased dissemination while suppressing invasion of both hyper-invasive partners (Fig. 3a, c–f). This asymmetric interaction indicates a competitive advantage for ATCC during co-infection. Notably, ATCC uniquely encoded several toxin- and host-modulation-associated determinants, including the autotransporter toxins Sat and Pic, hemolysin-associated loci, and the immune-modulatory factor TcpC. In addition, the strain carries an inactivated rpoS allele, which may influence stress adaptation and host-associated behaviour (Supplementary Fig. 2g). Together, these features may contribute to competitive exclusion and altered intracellular persistence phenotypes, although the precise mechanisms remain to be determined. Such dominance dynamics in polymicrobial infections may reshape invasion hierarchies and influence disease progression^65^.

**Fig. 4:**
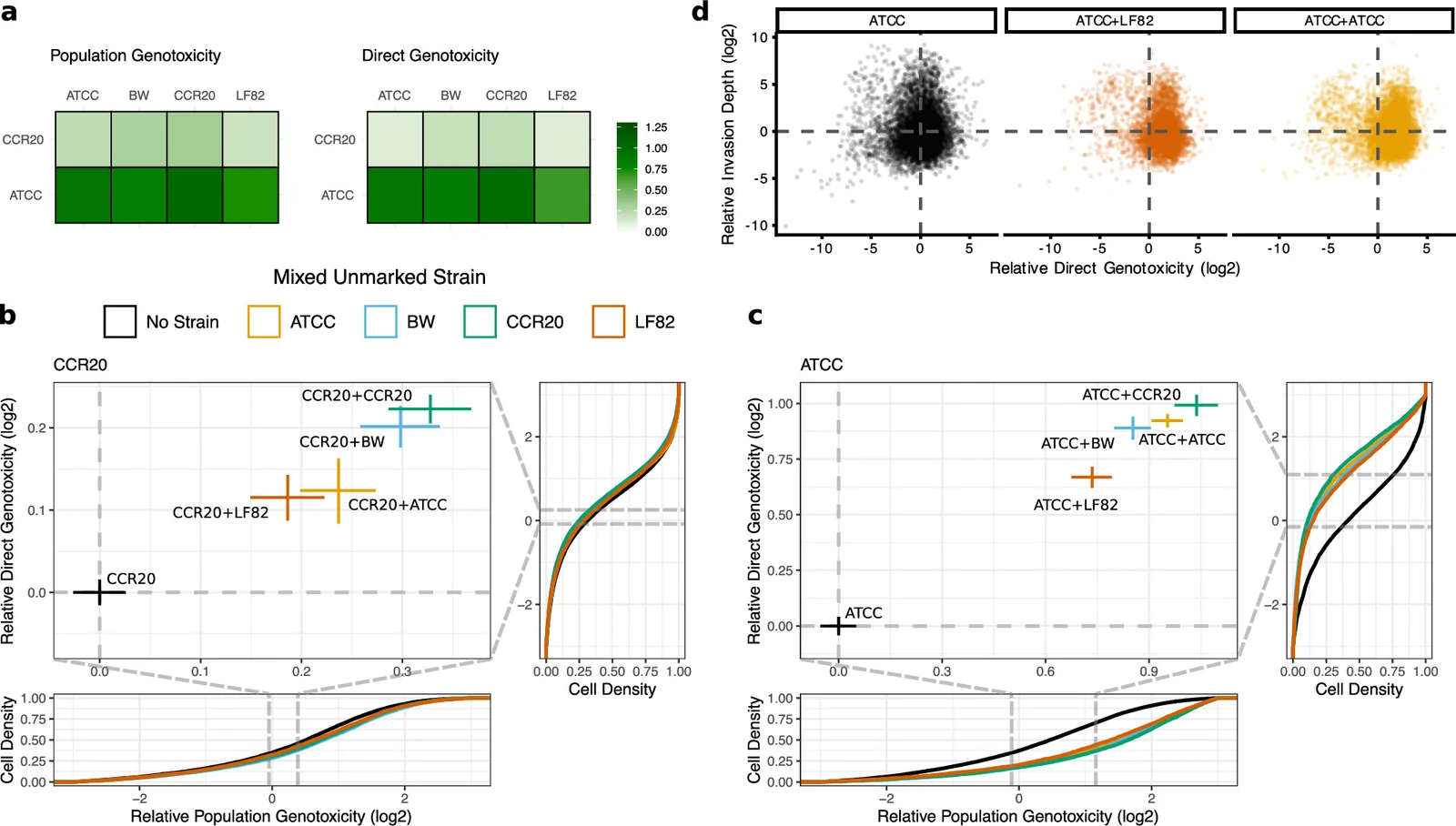
Inter-strain interactions enhance the genotoxic potency of colibactin-producing *E. coli* strains*.* **a** Genotoxicity modulation matrix: unlabelled strains (columns) were co-cultured with the two labelled genotoxic strains (rows), and changes in direct and population-level genotoxicity are shown relative to the effect of the corresponding labelled single-strain reference. **b**, **c** Genotoxicity modulation landscapes for CCR20 (**b**) and ATCC (**c**) in the presence of various unlabelled strains. Each plot shows changes in population genotoxicity versus direct genotoxicity relative to the labelled single-strain reference, indicated by the black cross in each plot. Geometrically normalised cumulative density plots show single-cell distributions of relative direct and population genotoxicity for the labelled strain, either alone (black) or mixed with various unlabelled strains—ATCC (orange), BW (blue), CCR20 (green), and LF82 (red). **d** Comparative scatter plot of direct genotoxicity versus invasion depth for single cells identified as labelled ATCC–infected, either infected alone (black) or co-infected with unlabelled ATCC (orange) or LF82 (red), shown relative to the mean of the labelled ATCC control. Each point represents a single cell measurement aggregated across replicates. Data were derived from three biological replicates, each with at least three technical replicates (Supplementary Data 2). Errors represent s.e.m.

To explore how cooperative and competitive invasion dynamics shape genotoxicity, we examined the impact of bacterial co-infections on the colibactin-producing strains ATCC and CCR20. Relative genotoxicity was quantified by comparing each co-infection to its corresponding single-strain baseline (Fig. 4a). Under single-strain baseline conditions, ATCC showed moderate population-level and direct genotoxicity, which markedly increased upon self-mixing (ATCC + ATCC), more than for CCR20, suggesting density-dependent colibactin production^66^ (Figs. 2b and 4a, c, d). Strikingly, we found that co-infection with non-genotoxic strains (LF82 or BW) increased both direct and population-level genotoxicity of the colibactin producers, particularly ATCC, implying that non-genotoxic partners can potentiate colibactin-associated DNA damage (Fig. 4a–d)^67,68^. This observation, together with the reduced invasion depth of non-genotoxic strains co-infected with the genotoxic ATCC variant (Fig. 3a, b, d), supports colibactin-mediated intracellular exclusion, possibly driven by increased mutational burden or phage activation^67,68^. To test this directly, we compared the invasiveness of fluorescent LF82 co-infected with either wild-type ATCC or its non-genotoxic isogenic mutant lacking the terminal step of colibactin biosynthesis (“Methods”). The mutant was markedly less effective at suppressing LF82 invasion, confirming that intact colibactin is responsible for both host-cell genotoxicity and intracellular dominance (Supplementary Fig. 4).

Collectively, these results reveal a complex interplay between bacterial invasion and genotoxicity, where dominant strains suppress the invasiveness of co-infecting bacteria while amplifying their own genotoxic potential. MALVINA’s single-cell, multiparametric readouts provide a powerful framework to dissect these interactions and to identify microbial or probiotic strains capable of counteracting the invasiveness and genotoxicity of cancer-promoting pathogens.

### Drug-induced reshaping of pathogen invasion and host-cell DNA damage

To assess MALVINA’s applicability for analysing drug-induced modulation of pathogen-host interactions, we screened 23 compounds, including antibiotics, biocides, and chemotherapeutics, for their effects on two key phenotypes: host-cell invasion and genotoxicity. Each compound was tested at 1× and 2× its approximate minimum inhibitory concentration (Supplementary Table 1). In parallel, extracellular bacterial counts from drug-treated co-cultures were quantified by colony-forming unit assays to directly assess each compound’s bactericidal activity (Fig. 1a, “Methods”). This enabled assessment of whether reductions in invasion or genotoxicity were attributable to reduced extracellular bacterial viability during infection, rather than to drug-associated modulation of these phenotypes. To evaluate strain specificity, screens were performed on colorectal epithelial cells infected with either of two hyper-invasive *E. coli* strains: the genotoxic CCR20 and the non-genotoxic LF82.

**Fig. 5:**
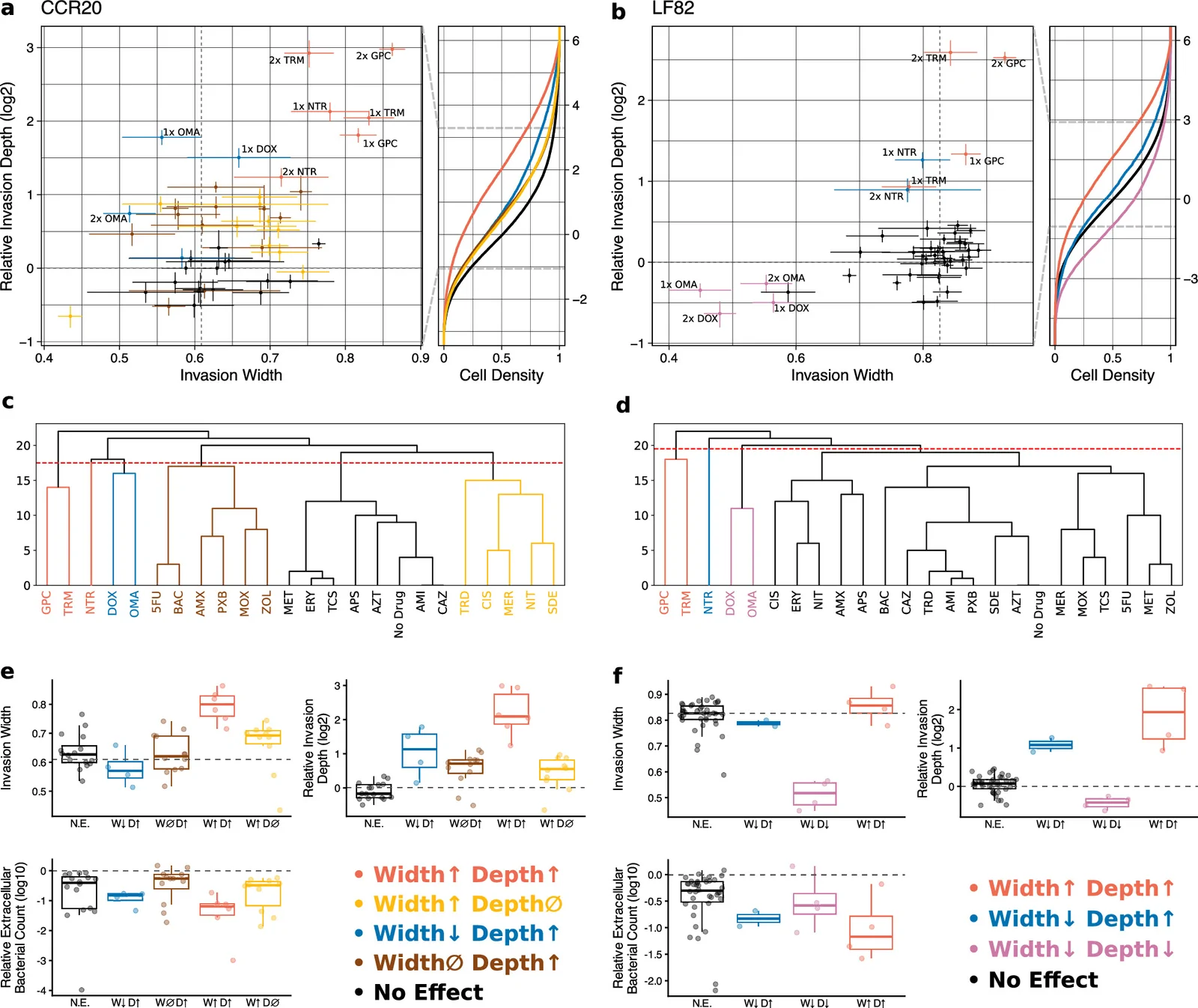
Drug-induced modulation of bacterial invasion. **a**, **b** Invasion width and depth for two hyper-invasive *E. coli* strains CCR20 (**a**) and LF82 (**b**) in the presence of various drugs and doses (1x, 2x; Supplementary Table 1). The invasion depth is shown relative to the respective untreated controls. Points are colour-coded by invasion-modulation drug class (see the legends of (**e**) and (**f**)). Geometrically normalised cumulative density plots show single-cell distributions of relative invasion depth across the invasion-modulation drug classes. **c**, **d** Agglomerative hierarchical clustering of the invasion metrics (width and depth at the two tested doses per drug) for CCR20 (**c**) and LF82 (**d**). In the dendrograms, simultaneous increases (decreases) in both invasion width and depth are coloured red (pink). Selective increases in width only are shown in yellow, depth-only increases in brown, and opposing effects with width decreases and depth increases are indicated in blue. Drugs with invasion width and depth metrics close to the untreated control (labelled “No Drug”) are coloured black (regarded as “No Effect”). Red dashed lines indicate cutoff heights used to define clusters; branches sharing a colour belong to the same response class. **e**, **f** Boxplots summarising invasion changes (invasion depth relative to the untreated controls) and extracellular bacterial counts (also relative to the untreated controls) in CCR20 (**e**) and LF82 (**f**). Dots represent drug treatments separately at the two tested doses and are colour-coded by invasion-modulating drug class. Data were derived from three biological replicates (Supplementary Data 3). Boxplot hinges represent the first and third quantiles, and the upper (lower) whisker extends to the largest (lowest) value no further than 1.5 times the distance between hinges. Errors represent s.e.m.

Bacterial invasion was quantified using two metrics: invasion depth (bacterial load per infected cell relative to untreated controls) and invasion width (fraction of infected cells) (Fig. 5a, b). Hierarchical clustering of these metrics across treatments and concentrations revealed distinct, strain-specific invasion-modulatory profiles (Fig. 5c, d). Three compounds—gepotidacin (GPC), trimethoprim (TRM), and N-nitroso-N-ethylurea (NTR)—showed the most pronounced and consistent effects. Notably, GPC and TRM significantly increased both invasion width and depth in CCR20 and LF82, despite reducing extracellular bacterial counts by up to three orders of magnitude, indicating drug-induced modulation of invasion phenotypes independent of bacterial load (Fig. 5). This paradoxical effect is striking given gepotidacin’s status as a recently approved dual-target antibiotic^69^. The carcinogen NTR increased both invasion metrics in CCR20, whereas in LF82 it selectively enhanced invasion depth (Fig. 5e, f). Omadacycline (OMA) and doxycycline (DOX) exhibited a similar strain-dependent pattern, selectively increasing invasion depth in CCR20 while reducing both invasion width and depth in LF82 infections (Fig. 5a, c, e). Clustering also identified antibiotic groups that moderately enhanced at least one invasion parameter in CCR20, reinforcing a strain-dependent invasion modulation landscape (Fig. 5c). Moreover, drugs acting on the same bacterial cellular targets or processes showed significantly more similar invasion-modulation profiles than those acting on different targets, a pattern consistent across both strains (Supplementary Fig. 5). These findings suggest that an antibiotic’s mechanism of action determines how it modulates pathogen invasion.

To assess whether invasion-modulating drug effects arise from direct bacterial responses or altered host susceptibility, we performed follow-up pre-treatment assays with selected compounds (TRM, GPC, NTR and OMA) that showed the most pronounced invasion-modulatory effects in the screen. In these experiments, performed in both CCR20 and LF82 infections, either bacteria or host cells were exposed to the drugs, followed by washout prior to infection (“Methods”). Short (1.5 h) bacterial pre-exposure induced only subtle increases in invasion width and/or invasion depth under selected conditions (Supplementary Fig. 7g, h). In contrast, host-cell pre-treatment for 4 h alone did not enhance invasion and instead resulted in a modest global reduction across the tested conditions (Supplementary Fig. 7e, f). Importantly, the strongest invasion-modulating effects were observed only during co-treatment conditions, in which bacteria, host cells, and drugs were present simultaneously during infection (Fig. 5a, b). These findings indicate that the observed invasion phenotypes emerge primarily from dynamic host-pathogen-drug interactions rather than from major drug-induced host-cell damage or epithelial barrier disruption, consistent with independent measurements of epithelial integrity and membrane permeability (Supplementary Fig. 7a–d).

To further assess whether invasion modulation reflected bacterial clearance, we compared drug-induced changes in invasion traits with extracellular bacterial counts for each strain. The absence of strong positive correlations indicated that invasion modulation was not attributable to bactericidal activity alone. Several drugs that markedly reduced bacterial loads simultaneously enhanced invasion parameters (Fig. 5e, f and Supplementary Fig. 8a, b, e, f). Remarkably, 16 of the 23 compounds screened (70%) shifted at least one invasion metric in one or more strains, even though the library was assembled with no prior knowledge of invasion-modulatory activity. These findings indicate that chemical modulation of bacterial invasion during host-pathogen interactions is a widespread pharmacological phenomenon. By disentangling invasion-modulating effects from general antibacterial activity, MALVINA provides a quantitative platform for mapping pro- and anti-invasive drug responses.

**Fig. 6:**
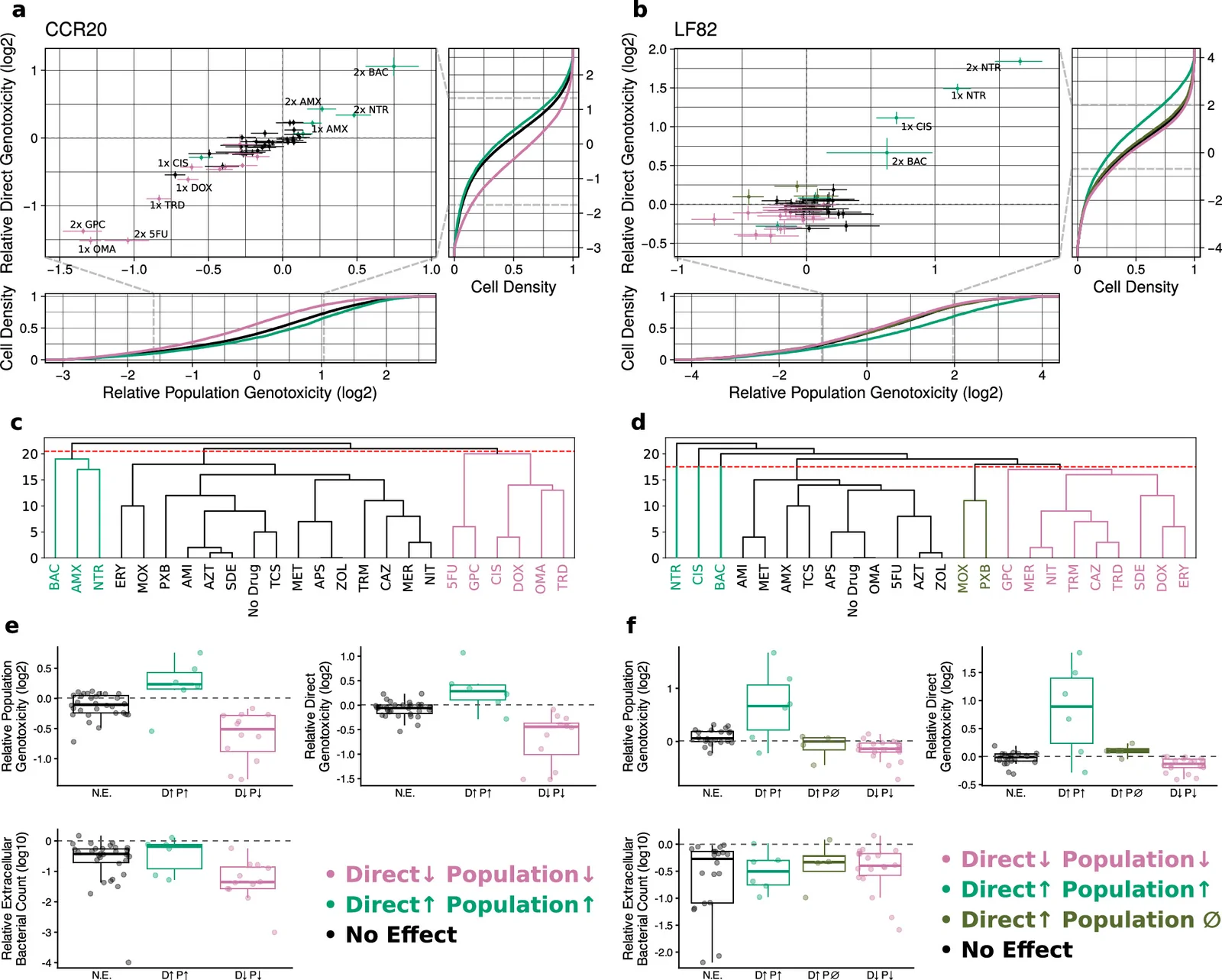
Drug-induced modulation of host-cell DNA damage. **a**, **b** Population and direct genotoxicity for two hyper-invasive *E. coli* strains CCR20 (**a**) and LF82 (**b**) in the presence of various drugs and doses (1x, 2x; Supplementary Table 1), shown relative to the respective untreated controls. Points are colour-coded by genotoxicity-modulation drug class (see the legends of (**e**) and (**f**)). Geometrically normalised cumulative density plots display single-cell distributions of both genotoxicity metrics across the different genotoxicity-modulation drug classes. **c**, **d** Agglomerative hierarchical clustering of the genotoxicity metrics (population and direct genotoxicity at the two tested doses per drug) for CCR20 (**c**) and LF82 (**d**). In the dendrograms, simultaneous increases (decreases) in both genotoxicity metrics are coloured green (pink); selective direct genotoxicity increases are coloured dark green. Drugs with genotoxicity metrics close to the untreated control (labelled “No Drug”) are coloured black (regarded as “No Effect”). Red dashed lines indicate cutoff heights used to define clusters; branches sharing a colour belong to the same response class. **e**, **f** Boxplots summarising genotoxicity changes (population and direct genotoxicity relative to the untreated controls) and extracellular bacterial counts (also relative to the untreated controls) in CCR20 (**e**) and LF82 (**f**). Dots represent drug treatments separately at the two tested doses and are colour-coded by genotoxicity-modulation drug class. Data were derived from three biological replicates (Supplementary Data 4). Boxplot hinges represent the first and third quantiles, and the upper (lower) whisker extends to the largest (lowest) value no further than 1.5 times the distance between hinges. Errors represent s.e.m.

Concurrent with invasion profiling, we simultaneously quantified drug-induced host-cell DNA damage during infection. Both direct and population-level genotoxicity were determined relative to untreated controls (Fig. 6a, b). Stratifying genotoxicity modulation by bacterial target revealed that, in LF82 infection, drugs acting on the same bacterial structure exhibited significantly more similar genotoxicity profiles than those acting on different targets (Supplementary Fig. 6). These findings indicate that an antibiotic’s target influences its genotoxicity modulation, particularly in infections with the non-genotoxic LF82 strain, where antibiotic-induced host cell DNA damage becomes more apparent in the absence of bacterial genotoxins.

Consistent with this, drug-modulated host genotoxicity was pathogen-dependent, likely reflecting intrinsic differences in the genotoxic potential of the two strains (Fig. 6). Polymyxin B (PXB) and moxifloxacin (MOX) selectively increased direct genotoxicity in LF82-infected cells but not in CCR20 (Fig. 6b, d, f). In contrast, DNA-damaging agents such as benzalkonium chloride (BAC) and N-nitroso-N-ethylurea (NTR) elevated both direct and population-level genotoxicity in infections with either strain, consistent with their known genotoxic properties (Fig. 6)^70–72^. The chemotherapeutic cisplatin (CIS) produced an opposing pattern, enhancing host-cell genotoxicity in LF82 infections while reducing it in CCR20 (Fig. 6)^73,74^. This likely reflects cisplatin’s bactericidal activity against CCR20, which lowers extracellular bacterial abundance and thus host exposure to colibactin (Fig. 6a, c, e). Supporting this, extracellular bacterial counts after treatment correlated moderately with host DNA damage in CCR20- but not LF82-infected samples (Supplementary Fig. 8c, d, g, h).

Together, these results indicate that in infections with genotoxin-producing pathogens, antibiotics can modulate host genotoxicity both directly, by inhibiting bacterial genotoxin production, and indirectly, by reducing extracellular bacterial loads and consequently decreasing toxin exposure. Notably, several compounds, including omadacycline (OMA), cisplatin (CIS), gepotidacin (GPC), tridecaptin (M152-P3 variant; TRD), doxycycline (DOX), and 5-fluorouracil (5-FU), reduced both direct and population-level genotoxicity in CCR20 infections (Fig. 6a, c, e).

### Dissecting drug effects on pathogen-associated genotoxicity

**Fig. 7:**
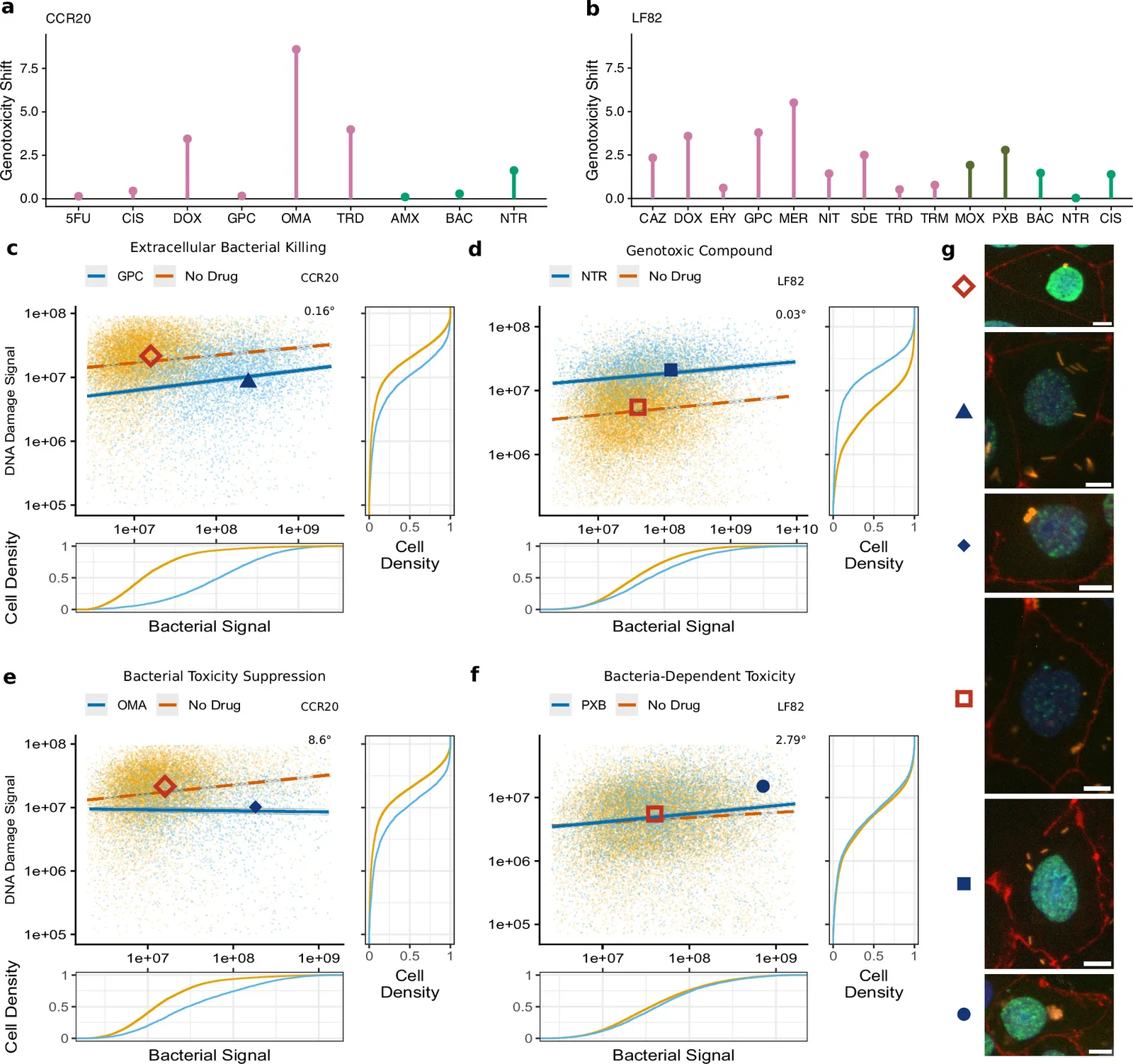
Genotoxicity shift reveals direct drug effects on bacteria-mediated genotoxicity. **a**, **b** Genotoxicity shifts for drugs from different genotoxicity-modulation classes during CCR20 (**a**) or LF82 (**b**) infection (see Fig. 6). The shift is defined as the absolute change in the slope angle of the log–log relationship between intracellular bacterial load and DNA damage in infected host cells, measured between drug-treated and untreated conditions (“Methods”). Bars are colour-coded by genotoxicity-modulation drug class (see legend of Fig. 6e, f). **c**–**f** Exemplars of genotoxicity shifts. Non-shifting drugs leave bacterial load–dependent genotoxicity unchanged (**c** gepotidacin, GPC ; **d** N-nitroso-N-ethylurea, NTR), whereas shifting drugs alter the intracellular bacteria–dependent dynamics of genotoxicity (**e** omadacycline, OMA; **f** polymyxin B, PXB). Examples are shown for CCR20 (**c**, **e**) and LF82 (**d**, **f**) infections. Bacterial and DNA damage signals of individual infected human cells are plotted and fitted by linear regression l. The genotoxicity shift angle (top right of each plot) quantifies the difference between untreated (blue) and treated (orange) conditions. Geometrically normalised cumulative density plots show the distributions of intracellular bacterial and DNA damage signals in infected cells, colour-coded as untreated (blue) or treated (orange). **g** Representative single-cell images of the effects of gepotidacin (GPC, blue upwards triangle), N-nitroso-N-ethylurea (NTR, blue square), omadacycline (OMA, blue diamond), and polymyxin B (PXB, blue circle); and the CCR20 (orange hollow diamond) and LF82 (orange hollow square) untreated controls, as indicated in (**c**–**f**). Data were obtained from three biological replicates at two doses per drug.

To determine whether drug-induced changes in genotoxicity resulted from direct modulation of bacteria-mediated DNA damage or indirectly from reduced extracellular bacterial load or drug-induced host cell toxicity, we introduced an invasion-normalised metric termed the genotoxicity shift (Fig. 7 and Supplementary Figs. 9 and 10). This metric, calculated from co-treatment measurements where drugs are present during host-pathogen interaction, quantifies the absolute change in the slope angle of the log–log relationship between intracellular bacterial load and host DNA damage under drug-treated versus untreated conditions (Fig. 7; “Methods”). The angle reflects how genotoxicity scales with intracellular bacterial burden, and its deviation from the untreated control indicates that the drug alters bacterial load–dependent facets of host cell genotoxicity.

As expected, the classical DNA-damaging agent N-nitroso-N-ethylurea (NTR) produced near-zero genotoxicity shifts in LF82-infected cells, where DNA damage increased proportionally with bacterial load, mirroring the pattern observed in untreated controls (Figs. 6b, d, f and 7b, d, g). In contrast, polymyxin B (PXB) and moxifloxacin (MOX) induced elevated direct genotoxicity in LF82 infections (Fig. 6b, d, f), with genotoxicity shifts indicating increased DNA damage per intracellular bacterium beyond what could be explained by the drugs’ intrinsic toxicity (Fig. 7b, f, g and Supplementary Fig. 10). Three non-exclusive mechanisms may underlie this effect: (1) bacterial modification of the drugs enhancing local genotoxicity; (2) drug-induced secretion of bacterial virulence factors that damage host DNA; or (3) infection-mediated sensitisation of host cells, increasing susceptibility to DNA damage^25,75–80^.

In CCR20 infections, host DNA damage is primarily driven by colibactin biosynthesis, a pathway highly sensitive to disruptions in bacterial viability and metabolism. Accordingly, gepotidacin (GPC), cisplatin (CIS), and 5-fluorouracil (5-FU) produced near-zero genotoxicity shifts in CCR20 co-cultures, consistent with their strong bactericidal activity at the tested doses (Figs. 6a, c, e, 7a, c, g and Supplementary Fig. 9). This pattern indicates that reduced host DNA damage primarily resulted from bacterial clearance and the consequent decrease in extracellular toxin levels, rather than from direct inhibition of pathogen-mediated genotoxicity. In contrast, omadacycline (OMA), doxycycline (DOX) and tridecaptin (M152-P3 variant; TRD) markedly reduced direct genotoxicity despite increasing CCR20 invasion depth (Figs. 5a, c, e, 6a, c, e, 7a, e, g and Supplementary Fig. 9). These effects were specific to the genotoxic CCR20 strain, suggesting that the compounds lower host DNA damage by interfering with colibactin production or activity.

These findings highlight that even without genotoxin production, bacterial infection can alter host responses to drugs. Moreover, some compounds directly attenuate pathogen-mediated genotoxicity through mechanisms beyond bacterial killing or invasion modulation. Integrating single-cell measurements of intracellular bacterial load with host DNA damage provides a powerful workflow to dissect multifactorial drug effects on host-pathogen interactions, identify compounds that modulate pathogen-associated genotoxicity, and evaluate therapeutic safety in bacterial infection models.

## Discussion

In this study, we present MALVINA, a Machine Learning–Based Virulence Interaction Analysis framework that integrates pathogen-host co-culture, immunofluorescence staining, and deep learning–based image analysis to quantify multiple facets of bacterial virulence at single-cell resolution. MALVINA introduces several advances. It enables simultaneous quantification of bacterial invasion and host-cell genotoxicity in a unified, high-throughput assay. By capturing thousands of single cells per well, it generates a detailed “virulence scorecard” comprising invasion width (proportion of infected cells), invasion depth (bacterial load per infected cell), and genotoxicity metrics that distinguish direct DNA damage in infected cells from population-level effects in uninfected cells. In addition, MALVINA disentangles pathogen-driven and indirect genotoxic effects, while its machine learning classifier ensures high-quality, artefact-free single-cell data.

Using MALVINA in a colorectal epithelial cell–*Escherichia coli* infection model, we uncovered several conceptually novel findings to be explored further in future studies. First, we identified distinct, strain-specific virulence profiles among *E. coli* isolates, revealing substantial variability in two key parameters: bacterial dissemination across the cell layer (invasion width) and intracellular bacterial burden (invasion depth). Some isolates showed high levels in only one of these dimensions, indicating mechanistic divergence in invasion strategies. Second, MALVINA enabled the dissection of direct and population-level genotoxicity in both colibactin-producing and non-producing bacteria. The genotoxic CCR20 isolate exhibited markedly elevated DNA damage in infected and uninfected cells, consistent with potent colibactin activity. Its combination of high invasiveness and strong genotoxicity underscores multifactorial virulence and clinical relevance as a tumorigenesis-promoting strain. In contrast, the ATCC25922 strain showed a more moderate profile, suggesting variability in colibactin biosynthesis or activity. Importantly, MALVINA was readily adapted to an independent infection model involving lung epithelial cells and *Klebsiella pneumoniae* strains, demonstrating its applicability across distinct host-pathogen systems.

These findings demonstrate MALVINA’s ability to identify hypervirulent clinical strains with enhanced invasive and/or mutagenic potential. Looking ahead, the platform can also be applied to high-throughput screening of mutants^81–83^ and in vivo–evolved strain libraries^84,85^, as well as diverse natural isolate panels from genome-wide association studies^86,87^, to uncover new virulence factors and toxin biosynthetic pathways. Moreover, MALVINA enables mechanistic studies of how bacterial and host factors shape the interplay between invasion and genotoxic stress^88^.

Third, MALVINA enables analysis of cooperative and competitive dynamics in mixed bacterial infections. The hyper-invasive strain LF82 enhanced the invasion capacity of the otherwise non-invasive laboratory strain BW25113, illustrating cooperative bystander uptake^64^. Conversely, the ATCC25922 strain showed increased dissemination during co-infection while suppressing invasion of its competitor non-genotoxic partners, revealing striking asymmetry in virulence interactions. Fourth, we observed that co-infection with non-genotoxic hyper-invasive strains amplified the genotoxic potency of colibactin-producing strains, whereas a colibactin-deficient mutant was far less effective at suppressing invasion of the hyper-invasive strain, implicating colibactin in both competitive suppression and toxin potentiation.

These findings indicate that invasion and genotoxicity are dynamically rewired within microbial communities. Together, they demonstrate MALVINA’s potential to map interbacterial virulence-modulation networks, identify genetic determinants of microbial cooperation and competition, and guide the design of pathogen-tailored probiotics.

Fifth, MALVINA revealed that drugs can modulate bacterial invasion of host cells independently of bactericidal activity. Screening a diverse panel of compounds against two clinically relevant *E. coli* strains (CCR20 and LF82) showed that 70% altered at least one invasion parameter, often in a strain- and drug-specific manner. These invasion changes are not readily explained by host-cell damage, epithelial barrier disruption, or stable drug-induced changes in bacteria alone, suggesting that they arise from host-pathogen-drug interactions. Together, these findings indicate that chemical modulation of bacterial invasion is widespread yet underexplored. Notably, MALVINA revealed that gepotidacin, a recently approved bactericidal antibiotic, enhanced bacterial invasion of human cells despite its antibacterial activity, underscoring the importance of evaluating potential pro-virulent side effects during antimicrobial development^69^.

Finally, MALVINA detected subtle, pathogen-dependent alterations in drug-induced host DNA damage. Certain compounds increased host genotoxicity via mechanisms contingent on the intracellular bacterial load, suggesting bacterial modification of drug toxicity or host-cell sensitivity^25,75–80^. In contrast, omadacycline, doxycycline, and tridecaptin suppressed host DNA damage in cells infected with genotoxic strains despite increased intracellular bacterial burdens, suggesting interference with colibactin-associated genotoxicity.

Altogether, these findings further highlight MALVINA’s value in unravelling the complex triad of host-pathogen-drug interactions. The platform is well-suited to (1) identify pro-virulent drug effects, (2) evaluate bacterial enhancement of drug toxicity and (3) develop virulence-targeting treatments (Fig. 1).

Despite its broad utility, MALVINA has several limitations. First, it requires fluorescent tagging of bacteria, which may not be feasible for all strains or clinical isolates, although alternative stains could mitigate this^89,90^. Second, reliance on gentamicin for extracellular bacterial removal limits use with gentamicin-resistant strains; bacteriophages or bacteriolytic enzymes such as lysozyme may offer viable alternatives^91^. Third, MALVINA provides only a static snapshot, and live-cell-compatible reporters will be needed for real-time tracking of invasion and genotoxicity^89,92^. Finally, the model must be retrained for different host cell lines due to variations in morphology and staining. Nonetheless, these are primarily technical constraints that can be addressed through further development.

MALVINA is optimised for adherent human cell lines, enabling high-throughput profiling but not fully recapitulating tissue complexity or immune interactions. Future developments could extend the platform to organoids and additional fluorescent markers to capture host responses such as apoptosis, inflammation, or barrier integrity. With advances in machine learning, MALVINA is well-positioned to classify complex host phenotypes and deepen understanding of infection biology. It fills a critical gap in clinical microbiology and therapeutic discovery by supporting the development of anti-virulence, pathogen-tailored probiotics and therapeutics that account for the dynamic interplay among bacteria, drugs, and host cells.

## Methods

### Bacterial strains and human cancer cell lines

For the *Escherichia coli* infection experiments, four *E. coli* strains were used: a standard laboratory strain (BW25113, hereafter referred to as BW), a highly invasive clinical isolate (LF82), and two genotoxic clinical isolates (CCR20 and ATCC25922, hereafter referred to as ATCC)^7,56,58–60^. For the *Klebsiella pneumoniae* infection experiments, five strains were included: three reference strains, ATCC BAA-2524 (KP1), NCTC 13440 (KP3), and DSMZ 9376 (KP5), together with two clinical isolates (KP2, KP4). All strains were transformed with the mScarlet fluorescent reporter gene carried by the pZS2R vector (Cmᴿ), expressed under a constitutive λ phage promoter (Supplementary Figs. 2a and 3a). The YFP gene in the original pZS2R-YFP plasmid construct was replaced with the mScarlet reporter gene derived from the pMRE-Tn7-145 plasmid^93,94^. To generate an isogenic colibactin-deficient mutant (ATCC25922, hereafter referred to as ATCC(−)), genome engineering was performed using pORTMAGE and CRISPR-Cas9. During pORTMAGE, an ssDNA oligonucleotide encoding a clbQ allele with two premature stop codons was incorporated into the bacterial genome^95^. The oligonucleotide was designed according to established MAGE guidelines^96^ (5′-T*A*AGGAGTGGGACGATGAGTAATATCAGTTTGTATTG TTTGCCATAgTgAGGTGGTTCTGCCGCCATGTATTATAAATGGCGTAGCGTGCTG*G*). To increase the efficiency of mutant selection, a spacer sequence specific to the wild-type allele (spacer1: 5′-AAACATAATACATGGCGGCAGAACCACCTGAATAG; spacer2: 5′-AAAACTATTCAGGT GGTTCTGCCGCCATGTATTAT) was cloned into a plasmid encoding CRISPR-Cas9, as previously described^97^. All oligonucleotides were synthesised by Integrated DNA Technologies using standard desalting. Bacterial strains were stored as aliquots at −80 °C in a 1:1 mixture of 50% glycerol and LB medium. For short-term maintenance (up to 2 weeks), fluorescent strains were cultured on LB agar plates (1.5% agar) supplemented with 20 µg/mL chloramphenicol and stored at 4 °C.

The Caco-2 human colorectal adenocarcinoma cell line (ATCC HTB-37) was obtained from the American Type Culture Collection^57^. Caco-2 cells are adherent epithelial cells that differentiate into enterocyte-like monolayers, forming tight junctions and polarised epithelial structures, and are therefore widely used for studying intestinal epithelial barriers in pharmacological and toxicological research^98,99^. Consistent with previous reports under standard culture conditions, MUC2 staining in Caco-2 monolayers was minimal and comparable to that observed in the non-mucosal U2OS osteosarcoma reference cell line, indicating limited mucus production in this model system (Supplementary Fig. 2b)^100^. Consequently, the present study primarily captures direct epithelial–bacterial interactions rather than infection dynamics occurring in the presence of a mature mucus barrier. The A549 human alveolar basal epithelial adenocarcinoma cell line (86012804-1VL, Merck) and the U2OS human osteosarcoma cell line (ATCC HTB-96) were also obtained from authenticated cell repositories. Upon thawing, Caco-2 and U2OS cells were cultured and maintained in DMEM High Glucose (4.5 g/L) with L-Glutamine (#DMEM-HA, Capricorn Scientific) supplemented with 10% Fetal Bovine Serum (#FBS-HI-12A, Capricorn Scientific), 1% MEM Non-Essential Amino Acids (#NEAA-B, Capricorn Scientific), and 1% Penicillin-Streptomycin solution (#PS-B, Capricorn Scientific). Human alveolar basal epithelial A549 adenocarcinoma cells were maintained in DMEM/Ham’s F-12 medium with L-Glutamine (#DMEM-12-A, Capricorn Scientific) supplemented with 10% Fetal Bovine Serum (#FBS-HI-12A, Capricorn Scientific), 1% Penicillin-Streptomycin solution (#PS-B, Capricorn Scientific), and 1% MEM Non-Essential Amino Acids (#NEAA-B, Capricorn Scientific). Mycoplasma contamination was routinely monitored using the Lonza MycoAlert Detection Kit (#LT07-518, Lonza), following the manufacturer’s protocol. Once the cells reached 90% confluence, they were passaged and split. The cells were maintained for up to 4 weeks post-thaw.

### Plating human colorectal epithelial cancer cells prior to infection experiments

Human colorectal epithelial Caco-2 cells and human alveolar basal epithelial A549 adenocarcinoma cells were used at passage 2–3 post-thaw and cultured to 70–85% confluency in monolayers across two 10 cm tissue culture dishes. Prior to seeding, cells were washed twice with 1× phosphate-buffered saline (PBS; prepared from 10× stock, Capricorn Scientific, #PBS-10XA), then detached using trypsin-EDTA solution (Capricorn Scientific, #TRY-1B), and incubated for 5 min at 37 °C. The resulting suspension was resuspended in 2 mL of complete culture medium. A 10 μL aliquot of the suspension was mixed with 10 μL Trypan Blue (Gibco, #15250-061), and viable cells were quantified using the LUNA-II™ Automated Cell Counter (Logos Biosystems) with the following parameters: dilution factor = 2, noise reduction = 5, live cell sensitivity = 7, roundness = 85%, minimum cell size = 3μm, maximum cell size = 60μm, and declustering level = low. Based on the resulting cell count, 8000 viable cells per well were seeded into tissue culture-treated 96-well plates (Greiner Bio-One, #655090) and grown for 24 h at 37 °C in a humidified incubator with 5% CO₂ prior to the infection.

### Infection of human cells with bacteria

All infection experiments were performed in the appropriate human cell culture medium supplemented with 10 mM HEPES (Capricorn Scientific, #HEP-B) and without Penicillin-Streptomycin.

#### Comparative characterisation of pathogen virulence

Fluorescently labelled *E. coli* or *K. pneumoniae* strains were streaked from agar plates and cultured overnight at 37 °C with shaking at 600 rpm in the appropriate human cell culture medium. The following day, bacterial cultures were diluted in fresh appropriate human cell culture medium to an OD₆₀₀ of 0.5. To initiate infection, the medium in 96-well plates containing pre-formed human epithelial monolayers was replaced with 50 µL of fresh appropriate human cell culture medium and 50 µL of bacterial suspension, resulting in a final OD₆₀₀ of 0.25. Plates were incubated for 3 h for *E. coli* infections or 2 h for *K. pneumoniae* infections at 37 °C in a humidified atmosphere containing 5% CO₂. After co-incubation, the medium was replaced with fresh gentamicin-containing appropriate human cell culture medium (final concentration: 100 µg/mL) for 6 h at 37 °C in a 5% CO₂ atmosphere to selectively eliminate extracellular bacteria. Following incubation, the cells were washed twice with 1× PBS (pH 7.5).

#### Mapping inter-strain virulence interactions

Fluorescently labelled and unlabelled strains were cultured overnight as above. The following day, the bacterial cultures were diluted to reach a final OD₆₀₀ of 0.5 in the appropriate human cell culture medium. To initiate co-infection, the medium in 96-well plates containing pre-formed human epithelial monolayers was replaced with 50 µL of fluorescently labelled and 50 µL of unlabelled bacterial suspension, each at OD₆₀₀ of 0.25, resulting in a final OD₆₀₀ of 0.5. Plates were incubated for 3 h at 37 °C in a humidified atmosphere with 5% CO₂ to allow strain-strain interactions during host cell invasion. After co-incubation, the medium was replaced with fresh gentamicin-containing appropriate human cell culture medium (final concentration: 100 µg/mL) for 6 h at 37 °C in a 5% CO₂ atmosphere to selectively eliminate extracellular bacteria. Following incubation, the cells were washed twice with 1× PBS (pH 7.5) prior to fixation and staining.

#### Drug screening assays

Twenty-three compounds were selected to represent mechanistically diverse perturbations relevant to host-pathogen interactions, including antibiotics targeting distinct bacterial processes, antimicrobial peptides and biocides, as well as chemotherapeutic and DNA-damaging agents known to affect host-cell physiology (Supplementary Table 1). This compound diversity enabled systematic assessment of how distinct pharmacological mechanisms influence bacterial invasion and genotoxicity phenotypes. These drugs were tested at two concentrations: one approximating the minimum inhibitory concentration (1×) for the target pathogens and another at twice that level (2×) (Supplementary Table 1). Each compound was pre-dispensed in 10× concentrated form relative to the 1× dose into 96-well plates and stored at −80 °C until use. Prior to infection, human epithelial monolayers were pre-treated for 1 h with 10 µL (1×) or 20 µL (2×) of the drug stock, added to 80 µL or 70 µL of fresh appropriate human cell culture medium, respectively. For untreated controls, the equivalent volume of sterile water (the solvent of the drugs) was added. In parallel, overnight bacterial cultures were diluted to OD₆₀₀ = 2.5 in the appropriate human cell culture medium. Following drug pre-incubation, 10 µL of the bacterial suspension was added to each well to achieve a final OD₆₀₀ of 0.25. Plates were incubated for 3 h at 37 °C in a humidified incubator with 5% CO₂. After co-incubation, the drug-containing infection supernatants were collected for extracellular bacterial quantification (see “Estimation of extracellular bacterial counts following drug treatment”). The monolayers were then immediately treated with fresh gentamicin-containing appropriate human cell culture medium (final concentration: 100 µg/mL) for 6 h at 37 °C in a 5% CO₂ atmosphere to selectively eliminate extracellular bacteria. Following incubation, the cells were washed twice with 1× PBS (pH 7.5) prior to fixation and staining.

#### Bacterial pre-treatment assays

Bacterial suspensions were pre-exposed to the selected compounds (OMA, TRM, NTR, GPC at 1× or 2× concentrations; Supplementary Table 1) for 1.5 h at 37 °C prior to host-cell contact. This pre-incubation period was selected to allow drug-induced bacterial phenotypic responses to develop before infection. Following treatment, bacterial cultures were washed once in the appropriate human cell culture medium containing 0.005% Tween-20 (Sigma-Aldrich) to minimise aggregation potentially induced by compound exposure and resuspended in fresh appropriate human cell culture medium to an OD₆₀₀ of 2.5. Untreated control bacteria were processed identically, including the washing step. Subsequently, 5 µL of the pre-treated bacterial suspension was added to pre-formed Caco-2 monolayers in a final assay volume of 100 µL, resulting in a final OD₆₀₀ of 0.125. Plates were incubated for 1.5 h at 37 °C in a humidified atmosphere containing 5% CO₂. After co-incubation, the infection medium was replaced with fresh gentamicin-containing appropriate human cell culture medium (100 µg/mL) for 6 h to eliminate extracellular bacteria. Following incubation, the cells were washed twice with 1× PBS (pH 7.5) prior to fixation and staining.

#### Host-cell pre-treatment assays

Pre-formed Caco-2 monolayers were pre-treated with GPC, NTR, OMA, or TRM for 4 h prior to infection (Supplementary Table 1). Following compound exposure, monolayers were washed twice with fresh appropriate human cell culture medium to remove residual drug before infection. Untreated bacterial suspensions were then added at a final OD₆₀₀ of 0.25 and incubated with the monolayers for 3 h at 37 °C in a humidified atmosphere containing 5% CO₂. Following infection, cells were treated with gentamicin-containing appropriate human cell culture medium (100 µg/mL) for 6 h to selectively eliminate extracellular bacteria. Following incubation, the cells were washed twice with 1× PBS (pH 7.5) prior to fixation and staining.

### Immunofluorescence staining of cell lines

Cells were fixed by adding 50 µL of 4% paraformaldehyde (PFA; Thermo Scientific Chemicals, #J19943.K2) in 1× PBS and incubating for 10 min at room temperature. Cells were then permeabilised with 50 µL of 0.5% Triton X-100 in 1× PBS for 10 min at room temperature and subsequently washed with 50 µL of 0.1% Triton X-100 in 1× PBS.

For Caco-2 immunostaining, rabbit anti-ZO-1 polyclonal antibody (Invitrogen, #61-7300; 1:300) and mouse anti-phospho-Histone H2A.X (Ser139) monoclonal antibody (clone 3F2; Invitrogen, #MA1-2022; 1:500) were diluted in antibody dilution buffer (1 part fetal bovine serum and 2 parts 0.1% Triton X-100 in 1× PBS), and cells were incubated overnight at 4 °C. Following a wash with 0.1% Triton X-100 in 1× PBS, cells were incubated for 1 h at 4 °C with Alexa Fluor 647-conjugated donkey anti-rabbit IgG (H + L) (Jackson ImmunoResearch, #711-605-152; 1:500) and Alexa Fluor 488-conjugated goat anti-mouse IgG (H + L) (Invitrogen, #A11001; 1:700).

For A549 immunostaining, rabbit anti-GLUT1 polyclonal antibody (Proteintech, #21829-1-AP; 1:500) and mouse anti-phospho-Histone H2A.X (Ser139) monoclonal antibody (clone 3F2; Invitrogen, #MA1-2022; 1:500) were diluted in antibody dilution buffer (1 part fetal bovine serum and 2 parts 0.1% Triton X-100 in 1× PBS), and cells were incubated overnight at 4 °C. Following a wash with 0.1% Triton X-100 in 1× PBS, cells were incubated for 1 h in the dark at 4 °C with Alexa Fluor 647-conjugated donkey anti-rabbit IgG (H + L) (Jackson ImmunoResearch, #711-605-152; 1:500) and Alexa Fluor 488-conjugated goat anti-mouse IgG (H + L) (Invitrogen, #A11001; 1:700).

Subsequently, nuclei were stained with Hoechst 33342 (Invitrogen, #H3570; 1:10,000) for 20 min. Finally, cells were washed once with 1× PBS and maintained in 60 µL 1× PBS at 4 °C until imaging.

### Estimation of extracellular bacterial counts following drug treatment

Following the 3 h pathogen–host co-culture incubation in the presence of various drugs, supernatants were collected for quantification of extracellular bacteria. Samples were serially diluted in fivefold steps (dilutions of 5^*n*^, where *n* = 1–8), and 7 µL droplets from each dilution were microplated using a Gilson 96-channel plate replicator onto single-well plates (Greiner) containing 45 mL of 70% LB agar supplemented with 20 µg/mL chloramphenicol. Plates were incubated for 18 h at 37 °C. After incubation, bacterial colonies expressing mScarlet were imaged using a custom-built imaging system equipped with a Basler a2A5328-15ucPRO camera (excitation: 556 ± 24 nm; emission: 690 ± 100 nm). Colony counts were performed manually, and colony-forming units (CFU) were calculated based on the dilution factor and the number of colonies per microplating spot. Extracellular bacterial counts were quantified using a custom R script. The input table was parsed using regular expressions into (1) droplet volumes per well, (2) colony count matrix, and (3) metadata. A sentinel value of 5000 flagged wells where colonies could not be enumerated due to confluent growth. For each well, colony counts <5000 were summed, scaled by a droplet-to-millilitre factor (1000 µL mL⁻¹), and divided by the plated volume to calculate CFU/mL. To normalise, CFU values were divided by the mean of matching untreated controls for each strain. Normalised CFU values were then log_10_-transformed.

### High-content imaging of the infection assay

Cells were imaged using an Operetta High-Content Imaging System (PerkinElmer) controlled by Harmony v3.5.1 software and equipped with a ×60 objective and a filtered xenon light source. For each well of the 96-well plates (excluding edge wells), 65 non-overlapping fields of view were acquired, corresponding to approximately 1000 cells per well on average. As samples were fixed prior to imaging, no environmental control was required. Fluorescence channels were acquired using the following excitation and emission filter sets: mScarlet, 520–550 nm excitation and 580–650 nm emission; Alexa Fluor 488 (γH2AX), 460–490 nm excitation and 500–550 nm emission; Alexa Fluor 647 (ZO-1/GLUT1), 620–640 nm excitation and 650–700 nm emission; Hoechst 33342, 360–400 nm excitation and 410–480 nm emission.

### Image processing

Raw high-content micrographs were imported into the Biological Image Analysis Software (BIAS) framework for processing^52^. A custom pipeline was developed within BIAS’s framework, complete with a cell approximation method and specialised machine learning classification algorithms. Nuclei were segmented using BIAS’s built-in deep visual network (Generic Nucleus Segmentation model) with an input spatial scaling factor of 0.7 and a segmentation window size of 1024 pixels^53^. Following segmentation, nucleus masks were dilated to approximate whole-cell regions. Three concentric zones were defined around the nucleus for each cell: (1) a perinuclear cytoplasmic region extending 10 μm beyond the nucleus, (2) a distal cytoplasmic region extending an additional 10 μm, and (3) a pericellular interstitial region extending a further 10 μm beyond the cytoplasmic edge (Supplementary Fig. 1). To avoid overlaps, dilations were halted in areas where cytoplasmic region borders touched. These concentric zones were defined to better distinguish bacterial adherence from invasion by accounting for potential non-specific adherence and extracellular bacterial accumulation.

Masks corresponding to nuclei, cytoplasm, and interstitial areas were aligned and used to extract image features. These features supported the classification of cells as infected, noninfected, or adherent, and enabled identification of overlapping as well as detached cells that were consequently out of focus. The integrated intensity of intracellular bacterial mScarlet fluorescence (calculated from the union of nuclear and cytoplasmic regions) and the γH2AX-associated Alexa Fluor 488 signal localised to the nucleus were exported alongside the classification results for downstream analysis. On average, over 60,000 cells were profiled per 96-well plate, with approximately 1000 cells per well (excluding edge wells).

### Machine learning model for infection classification

An image database was generated for training the neural network using segmented masks (see Image processing) from BIAS. The neural network classification was performed using the built-in Machine Learning Classification module on an NVIDIA 3080Ti graphics card.

In the case of gastrointestinal epithelial cells, a total of 2373 cells were manually classified as ground truth into three categories: noninfected, infected, or containing adherent bacteria (Supplementary Fig. 1). During model training and evaluation, the nuclear, perinuclear, distal, and interstitial masks were utilised along with unified cell masks. For each mask, the following features were incorporated: Hoechst and mScarlet Haralick textures, as well as integrated, maximal, mean, minimal, and median intensities and standard deviations. The model used a multilayer perceptron (MLP) classifier with the following settings: weight scale = 0.01, momentum = 0.01, maximum iterations = 10,000, epsilon = 0.0005, and hidden layer neurons = 11. The final model was validated using 10% cross-validation and achieved an accuracy of 86% (Supplementary Table 2).

In the case of lung epithelial cells (A549; for use in Supplementary Fig. 3), two successive models were used to determine infection: first, 1286 cells were manually classified as being infected or noninfected, with 643 members each. During model training and evaluation, the nuclear, perinuclear, distal, and interstitial masks were utilised along with unified cell masks. For each mask, the following features were incorporated: mScarlet Haralick textures, as well as integrated, maximal, mean, minimal, and median intensities. The model used a multilayer perceptron (MLP) classifier with the following settings: weight scale = 0.01, momentum = 0.31, maximum iterations = 10,000, epsilon = 0.0005, and hidden layer neurons = 2. The final model was validated using 10% cross-validation and achieved an accuracy of 83.44%. Afterwards, the cells labelled as infected were separated and classified again into infected or containing adherent bacteria (Supplementary Fig. 1). For the secondary model, the output of the previous model was used, with 176 cells classified as adherent-bacteria-containing cells and 167 classified as true infected cells. The model used a multilayer perceptron (MLP) classifier with the following settings: weight scale = 0.01, momentum = 0.01, maximum iterations = 10,000, epsilon = 0.0005, and hidden layer neurons = 12. The final model was validated using 10% cross-validation and achieved an accuracy of 91.84%. Classification performance was substantially improved by incorporating texture features, compared to models relying solely on fluorescence intensity measurements, underscoring the value of high-content feature integration for infection classification and artefact filtering (Supplementary Table 2).

### Machine learning model for discarding artefactual cells

An image database was generated for training the neural network using segmented masks (see Image processing) from BIAS. The neural network classification was performed using the built-in Machine Learning Classification module on an NVIDIA 3080Ti graphics card. In the case of gastrointestinal epithelial cells, a total of 592 cells were manually classified as ground truth into two categories: normal or artefactual (overlapping, detached that were consequently out of focus, etc.). During model training and evaluation, the nuclear, perinuclear, distal, and interstitial masks were utilised along with unified cell masks. Its input features include cell morphology descriptors (such as area, circularity, convexity and form factor), Haralick texture features and intensity measurements of all fluorophores (integrated, maximum, mean, median, minimum, and standard deviation). The model used a multilayer perceptron (MLP) classifier with the following parameters: maximum iterations = 10,000, weight scale = 0.01, momentum = 0.01, hidden layers = 0, and epsilon = 0.0005. The model was validated using cross-validation with a validation ratio of 10% and achieved an accuracy of 92.98% (Supplementary Table 2).

In the case of lung epithelial cells (A549; for use in Supplementary Fig. 3), a total of 257 cells were classified as ground truth: 124 as artefactual, and 133 as normal. During model training and evaluation, the nuclear, perinuclear, distal, and interstitial masks were utilised along with unified cell masks. Its input features include cell morphology descriptors (such as area, circularity, convexity and form factor), Haralick texture features and intensity measurements of all fluorophores (integrated, maximum, mean, median, minimum, and standard deviation). The model used a multilayer perceptron (MLP) classifier with the following settings: weight scale = 0.01, momentum = 0.01, maximum iterations = 10,000, epsilon = 0.0005, and hidden layer neurons = 3. The final model was validated using 10% cross-validation and achieved an accuracy of 85.07%.

### Metric calculations for virulence profiling

Quantitative metrics describing bacterial infection and host DNA damage were calculated using a custom analysis pipeline developed in R version 4.4.1  for the MALVINA system. This pipeline aggregates single-cell fluorescence features into well-level summaries and computes normalised virulence metrics across experimental conditions.

To ensure accuracy, cells identified as artefactual or located within adherent regions were excluded from analysis. The remaining cells were grouped by infection status within each well, and four key metrics were computed: invasion depth, invasion width, direct genotoxicity, and population genotoxicity (Figs. 1–6). Invasion depth was defined as the mean integrated intracellular bacterial signal, measured via mScarlet fluorescence, across infected cells in each well, with standard deviation calculated at the single-cell level. Invasion width was calculated as the proportion of infected cells relative to the total number of infected and noninfected cells. Direct and population genotoxicity were assessed as the mean nuclear γH2AX intensity, based on integrated Alexa Fluor 488 fluorescence, in infected and noninfected cells, respectively, with standard deviations similarly calculated across individual host cells per well.

To account for variability between experiments, normalisation was performed using control wells from each independent experiment as the reference. For invasion depth, BW was used as the reference strain in characterisation experiments, the labelled single-strain control in inter-strain virulence interaction analyses, and untreated wells in drug screening assays. Genotoxicity metrics were similarly normalised using either infected or noninfected cells from the appropriate control wells, depending on whether direct or population genotoxicity was being assessed. Standard deviations for invasion depth and genotoxicity metrics were propagated through the normalisation process using error propagation formulas, ensuring that uncertainty in the normalised values was accurately captured.

After well-level metrics were computed, residual Z-score filtering was applied to identify and discard divergent replicates separately for the invasion and genotoxicity metrics. If a control well was excluded during this process, all well-level normalised metrics were recalculated accordingly (see “Filtering of divergent replicates via residual Z-scores”). Final values for invasion depth, direct genotoxicity, and population genotoxicity were then averaged across both biological and technical replicates, with propagated standard errors calculated to reflect variability. Invasion width values were similarly averaged across replicates, with standard deviations calculated directly from the replicate measurements.

To evaluate whether the normalised virulence metrics captured true biological variation rather than experimental noise, we computed Euclidean distances between pairs of true experimental replicates and randomly selected data points from the same dataset (drug screen, strain and inter-strain interaction characterisation). Pairwise Euclidean distances were calculated in two metric spaces: the invasion plane (defined by invasion width and depth) and the genotoxicity plane (defined by direct and population-level genotoxicity). Statistical comparisons were performed using the two-sample Kolmogorov–Smirnov test (Supplementary Fig. 11). In the drug screen experiments, the distributions differed significantly for both invasion (D = 0.28, *p* = 4.7 × 10⁻¹²) and genotoxicity (D = 0.15, *p* = 1.3 × 10^−^^3^) metrics. Differences were even more pronounced in strain characterisation and inter-strain interaction experiments, where invasion (D = 0.16, *p* = 7.8 × 10^−^^82^) and genotoxicity (D = 0.18, *p* = 2.2 × 10^−^^105^) metrics showed highly significant separation from randomised distributions.

### Calculation of invasion interaction scores

To evaluate the effect of each unmarked strain on the invasion width and depth of the marked reference strain, we calculated directional interaction scores. We considered a neutral interval between the single-strain control and a self-mix where the unmarked strain was isogenic to the marked strain. Interactions falling into the neutral interval were defined as 0. Interactions that reduced invasion width or invasion depth below the neutral interval were calculated as the log2-fold change compared to the lower edge of the neutral interval (log2(xi,j/x↓i,j) for j metric of the *i*-th condition, where x↓ is the lower edge of the neutral interval). Interactions that increased invasion width or invasion depth above the neutral interval were calculated as the log2-fold change compared to the upper edge of the neutral interval (log2(xi,j/x↑i,j) for j metric of the *i*-th condition, where x↑ is the upper edge of the neutral interval) (Fig. 3a).

### Agglomerative clustering of drug effects on virulence

An agglomerative clustering function was developed based on the scikit-learn package in Python 3.11, and imported into the R environment via reticulate to produce numeric cluster codes and dendrograms^101^. All non-dendrogram graphics were created using ggplot2 and ggrepel (Figs. 5 and 6). The invasion clustering for each strain was created separately via merging the invasion width and invasion depth effects of both tested doses for each drug into a single point in a four-dimensional space. Euclidean distance matrices were calculated on the four-dimensional points of each drug and clustered with complete linkage. The genotoxic clustering was calculated similarly, using population and direct genotoxicity. Cut heights and the expected number of clusters were chosen empirically for every strain and virulence metric after visual inspection and were fixed for plot generation (Figs. 5c–d and 6c–d).

### Filtering of divergent replicates via residual Z-scores

Each replicate was screened for aberrant values by using an iterative, multivariate Z-score filtering algorithm. This filtering process began by grouping all virulence metrics by each fluorescently labelled strain, separately across different experimental settings—including single-strain infection, co-infection, and drug screening assays. These experimental supergroups served as a reference baseline to allow for standardised, consistent outlier detection. The calculated metrics of invasion width, invasion depth, population and direct genotoxicity were first converted into four Z-scores per replicate, Z_i,j_ = (x_i,j_ − x̄_j_)/s_j_ where x_i,j_ is the *j* metric of replicate *i*, x̄_j_ is the supergroup mean and s_j_ is the supergroup standard deviation. Each replicate was then separated into filtering groups per experimental condition. Inside each filtering group, residual Z-scores (Z_i,j_’) were calculated for every metric from the difference between each replicate and the median (Z_i,j_’ = Z_i,j_ − Z_j,m_ for every replicate *i* and metric *j*, where Z_j,m_ is the median metric of the filtering group), and the absolute sum of residuals provided the divergence score across invasion metrics and genotoxicity metrics (ⅆ_i_ = Σ(|Z_i,j_’|) for each replicate *i* and metric *j*). If any replicate exceeded the residual Z-score cutoff of 3 for at least one metric, the replicate with the highest divergence score was discarded. First, the filtering was applied to the invasion metrics of all replicates, then the remaining replicates were filtered for a second time, across genotoxicity metrics. All conditions included a minimum of three biological replicates, and filtering was constrained to retain at least two replicates per filtering group.

In the case of lung epithelial cells (A549; for use in Supplementary Fig. 3), additional filtering steps were used before the residual filtering due to the high signal variability in the A549 cell line. First, a cellular heterogeneity filter was used to discard unstable wells. This filtering step involved an upper Tukey-fence applied to the variability of the invasion depth, population and direct genotoxicity signals calculated from each cell in a given well, discarding wells with high heterogeneity. Then, on all four calculated metrics (invasion depth, invasion width, population and direct genotoxicity), a Tukey-fence was applied to discard wells from all wells infected with a given strain and the noninfected control, to filter outliers. Finally, the residual filtering algorithm was applied.

### Calculation of genotoxicity shifts

Cell-level data were filtered to include only non-artefactual, infected cells treated with drugs classified as host genotoxicity modulators (Fig. 6). Integrated single-cell DNA damage (D) and bacterial (b) signals were analysed exclusively on a log–log scale. For each drug-strain combination (combining both tested drug doses), the integrated fluorescence intensity of intracellular bacterial and DNA damage signals was fitted via linear regression: log_10_(D_(i)_) = α_0_ + α_1_ · log_10_(b_(i)_) + ε_(i)_ where *i* denotes the *i*-th  drug condition. The slope α_1_ was extracted and angularised (φ = atan α_1_ · 180° / π). For each strain, the non-drug-treated condition served as reference, and the treatment-specific genotoxicity shift was calculated as Δφ = |φ_Drug_ − φ_No Drug_| (Fig. 7 and Supplementary Figs. 9 and 10).

### SYTOX Blue accumulation assay

For cytotoxicity measurements, 8000 viable Caco-2 cells per well were seeded into tissue culture-treated 96-well plates (Greiner Bio-One, #655090) and cultured for 24 h at 37 °C in a humidified atmosphere containing 5% CO₂. Cells were then exposed to GPC, NTR, OMA, or TRM at 1× and 2× concentrations, as defined in the drug screening assays (Supplementary Table 1). Membrane integrity was assessed after 4 h and 24 h of treatment using the membrane-impermeant nucleic acid dye SYTOX Blue (1 μM final concentration; Thermo Fisher Scientific, #S11348), while total nuclei were counterstained with DRAQ5 (2.5 μM final concentration; Thermo Fisher Scientific, #65-0880-92). Dyes were added directly to the culture medium without washing steps, followed by incubation for 10 min at 37 °C prior to imaging. After the 4 h readout, cells were returned to the incubator without removal of either the compounds or the previously added dyes. At the 24 h endpoint, DRAQ5 was re-added at a final concentration of 2.5 μM, followed by a 10 min incubation prior to imaging.

SYTOX Blue signal was acquired using a Revvity Opera Phenix high-content imaging system. Nuclear staining was imaged using 640 nm excitation and a 690–720 nm emission filter, while SYTOX Blue was imaged using 405 nm excitation and a 435–480 nm emission filter. Images were acquired from the Caco-2 cell line used throughout the study using a ×60 water-immersion objective. Single-cell SYTOX measurements were obtained by expanding each nuclear mask up to a maximum radius of 20 μm, stopping when the expansion reached a neighbouring cell region. SYTOX accumulation was quantified as the integrated fluorescence intensity within the resulting cell-associated region. Distributions of single-cell measurements were generated for each treatment condition, and effect sizes relative to untreated controls were calculated using Glass’s Δ.

### Transepithelial electrical resistance (TEER) measurements

Polyester Transwell® inserts (12 mm diameter, 0.4 μm pore size; Corning Costar, #3460) were coated with 100 μL collagen solution (Cell Applications Inc., #125-50) for 1 h at 37 °C to promote cell attachment. Caco-2 cells were seeded at a density of 5 × 10⁴ cells per insert in 600 μL complete medium in the apical compartment, while the basolateral compartment contained 1700 μL of the same medium. Cells were cultured for 10 days under standard conditions to allow formation of differentiated, polarised epithelial monolayers, with medium replacement every 3 days. Monolayers reaching TEER values between 300 and 500 Ω·cm² were considered suitable for experiments.

Sixteen hours prior to treatment, culture medium was replaced with complete medium supplemented with 10 mM HEPES to maintain pH stability during measurements outside the incubator. Final medium volumes were adjusted to 775 μL (apical) and 1650 μL (basolateral). Cell-free inserts containing identical medium were prepared in parallel for background subtraction. Prior to measurement, plates were equilibrated at room temperature for 20 min to minimise temperature-dependent variability. TEER values were recorded using a Millicell® ERS 3.0 digital voltohmmeter (Millipore, #MERS03000) equipped with STX-type chopstick electrodes. Electrodes were sterilised in 70% ethanol, rinsed in sterile PBS, and equilibrated in culture medium before use.

Test compounds (GPC, NTR, OMA, and TRM) were applied simultaneously to the apical and basolateral compartments at 2× working concentrations (Supplementary Table 1). TEER measurements were recorded at 2, 4, 8, and 24 h post-treatment. Raw resistance values were corrected by subtracting the mean resistance of cell-free inserts and normalised to the membrane surface area according to the manufacturer’s specifications, yielding TEER values expressed as Ω·cm².

### Assessment of mucus production by immunofluorescence

Mucus production was assessed by immunofluorescent detection of MUC2 in epithelial cell lines with distinct secretory phenotypes. Caco-2 cells were analysed together with U2OS osteosarcoma cells (ATCC HTB-96) as a non-mucus-producing reference. Cells were maintained in the appropriate culture medium under standard conditions (37 °C, 5% CO₂, humidified atmosphere) and seeded at a density of 8000 cells per well in black, flat-bottom, tissue culture-treated 96-well plates (Greiner Bio-One, #655090).

After 24 h, cells were fixed with pre-chilled 70% ethanol for 30 min at 4 °C, followed by washes with phosphate-buffered saline (PBS). To ensure complete rehydration after ethanol fixation, cells were incubated in PBS for an additional 30 min at room temperature. Cells were then incubated overnight at 4 °C with a rabbit anti-MUC2 polyclonal antibody (Thermo Fisher Scientific, #PA5-79702; 1:500 dilution) diluted in PBS containing 5% fetal bovine serum and 0.1% Triton X-100. Following PBS washes, cells were incubated for 1 h at room temperature with an Alexa Fluor 555-conjugated anti-rabbit secondary antibody (Thermo Fisher Scientific, #A-21428, 1:700 dilution) prepared in the same buffer. Nuclei were counterstained with Hoechst 33342 (1:10,000 dilution) for 15 min at room temperature.

MUC2 immunofluorescence signal was acquired using a Revvity Opera Phenix high-content imaging system. Nuclear staining was imaged using 405 nm excitation and a 435–480 nm emission filter, while MUC2 staining was imaged using 561 nm excitation and a 570–630 nm emission filter. Images were acquired using a ×60 water-immersion objective from the Caco-2 cell line used throughout the study and the U2OS human osteosarcoma cell line, which served as a non-mucosal negative control. For each field of view, 16 z-planes were acquired at 1 μm intervals. Single-cell MUC2-associated fluorescence was quantified by expanding each nuclear mask up to a maximum radius of 20 μm, stopping when the expansion reached a neighbouring cell region. Fluorescence measurements were performed on the mean-intensity projection of the z-stack using integrated signal intensity. Distributions of single-cell measurements were generated for each cell line, and effect sizes were calculated using Cliff’s d.

### Genome sequencing

Illumina short-read sequencing was performed for all newly sequenced strains. Genomic DNA libraries were prepared from 1 ng of input DNA using the Nextera XT DNA Library Preparation Kit, Illumina, USA, Cat. No. FC-131-1096, with Nextera-compatible index primers, Integrated DNA Technologies, Coralville, IA, USA, following the manufacturer’s protocol. Sequencing-ready libraries were quality-controlled using a TapeStation 4200 instrument with D5000 ScreenTape, Agilent Technologies, USA, Cat. No. 5067-5588. Paired-end sequencing was performed on a NextSeq 2000 system using a P2 XLEAP-SBS Reagent Kit, 300 cycles, Illumina, USA, Cat. No. IL20100985, targeting 1 million paired-end reads per sample.

Oxford Nanopore Technologies long-read sequencing was performed for one strain, CCR20, to support hybrid genome assembly. For this strain, 200 ng of genomic DNA was used for library preparation with the Native Barcoding Kit 24 V14, Oxford Nanopore Technologies, Oxford, UK. DNA end-repair and dA-tailing were performed using NEBNext FFPE Repair Mix and the NEBNext Ultra II End Prep Kit, New England Biolabs, Ipswich, MA, USA, followed by purification with Mag-Bind TotalPure NGS beads, Omega Bio-tek, Norcross, GA, USA. Adapter ligation was performed using NEBNext Quick T4 DNA Ligase, New England Biolabs, and the ligated library was purified again using Mag-Bind TotalPure NGS beads. Library concentration was determined using a Qubit 3.0 Fluorometer, Thermo Fisher Scientific, Waltham, MA, USA. The final library was mixed with Oxford Nanopore running buffer and loading beads, loaded onto a FLO-MIN114 SpotON Flow Cell, Oxford Nanopore Technologies, and sequenced on a MinION device for 24 h.

### Assembly and annotation

Raw Illumina reads were quality-assessed using FastQC v0.12.1. Adapter removal and quality trimming were performed using Trimmomatic v0.40 in paired-end mode with Phred+33 encoding, using the adapters.fa file distributed with BBMap v38.90. The following parameters were applied: ILLUMINACLIP:adapters.fa:2:30:10 LEADING:15 TRAILING:15 SLIDINGWINDOW:5:15 MINLEN:100 HEADCROP:10. Oxford Nanopore Technologies (ONT) reads were adapter-trimmed using Porechop_ABI v0.5.1 in --ab_initio mode. Nanopore reads were filtered using NanoFilt v2.8.0, retaining reads with a minimum mean Phred quality score of 10 and a minimum read length of 1000 bp.

Genome sequences were obtained using three approaches, depending on data availability. For strains with publicly available genome assemblies, assemblies were retrieved from the NCBI RefSeq database including BW25113 (GCF_000750555.1), ATCC25922 (GCF_028596225.1), LF82 (GCF_021398935.1), NCTC 13440 (GCA_900451435.1), and DSM 9376 (GCF_013180515.1). For strains with Illumina short-read data only (KP1, KP2, KP4), de novo short-read assemblies were generated using Unicycler v0.5.1 in normal mode. For CCR20, which had both Illumina short-read and ONT long-read data, a hybrid assembly was generated using the same Unicycler version and mode.

Genome annotation was performed using two approaches. For assemblies retrieved from NCBI RefSeq, the corresponding RefSeq annotations were used. Assemblies generated in this study were annotated using Bakta v1.12.0 with the Bakta database [https://doi.org/10.5281/zenodo.14916843], supplemented with PKS island genes added as custom protein sequences.

### Virulence factor analysis

Virulence factor analysis was performed using protein sequence similarity searches with BLASTP v2.16.0 against the VFDB database (full dataset, set B; accessed February 13, 2026) [https://doi.org/10.1093/nar/gkae968]. Hits corresponding to virulence factors lacking experimental validation were excluded from further analysis. Searches were performed using a minimum query coverage per high-scoring segment pair of 80%, and hits were retained only if they showed at least 90% amino acid identity. For downstream analyses, only unique virulence factor gene hits were retained.

### Computational resources used

Analysis was performed on a single workstation computer. The graphics card used for analysis and classification was an NVIDIA GeForce RTX 3080 Ti with 12GB GDDR6X memory and 10240 CUDA cores. The CPU was an Intel(R) Core(TM) i9-10900F CPU @ 2.80 GHz CPU, and was supplied with 64 GB of RAM. The Operating System was Ubuntu 22.04.5 LTS (codename Jammy Jellyfish), which was allocated with a 64 GB swap partition for overflow protection. Due to the large data size used, a Seagate Ironwolf Pro 24 TB HDD was used for storage.

The software used for image analysis was BIAS (Biological Image Analysis Software). A practical advantage of BIAS is its support for direct import of proprietary image formats generated by a wide range of microscopy platforms. This functionality enables image analysis to be performed directly on native image files, avoiding intermediate conversion steps (e.g., TIFF or OME-TIFF) and facilitating retention of instrument-specific metadata that may otherwise be lost during format conversion.

### Reporting summary

Further information on research design is available in the Nature Portfolio Reporting Summary linked to this article.

## Supplementary information

Supplementary Information

Description of Additional Supplementary Files

Supplementary Dataset 1

Supplementary Dataset 2

Supplementary Dataset 3

Supplementary Dataset 4

Supplementary Dataset 5

Supplementary Dataset 6

Supplementary Dataset 7

Supplementary Dataset 8

Supplementary Dataset 9

Supplementary Dataset 10

Supplementary Dataset 11

Reporting Summary

Transparent Peer Review file

## Source data

Source Data

## Data Availability

The raw data generated in this study, including all microscopy images and microplate images of bacterial colonies, are deposited in the HUN-REN 23FTBV public data repository in the https://repo.researchdata.hu/dataverse/malvinamethod dataverse under accession links https://doi.org/10.5158/ARP/23FTBV and https://doi.org/10.5158/ARP/YORL70 for download. Raw sequencing data and genome assemblies generated as part of this work have been deposited in the European Nucleotide Archive (ENA) under the Study Accession number PRJEB120792. Source data are provided with this paper.

## Code availability

The custom scripts and image analysis pipelines developed for this study are deposited in the HUN-REN 23FTBV public data repository in the https://repo.researchdata.hu/dataverse/malvinamethod dataverse. The preview version of the image analysis software is available in the dataset https://doi.org/10.5158/ARP/EX0QQL, and the pipelines used are available at https://doi.org/10.5158/ARP/3XYKB8 for download; the scripts used for data analysis are available in the dataset https://doi.org/10.5158/ARP/OINNBW for review.
