# Supplementary material for: Deep-learning single-cell profiling uncovers interbacterial and drug-driven reshaping of pathogen virulence
Source: Nat Commun. 2026 Sep 29;17:11236. doi: 10.1038/s41467-026-77989-w (PMC13720132; doi:10.1038/s41467-026-77989-w)
Supplement: Supplementary file 1 [file 41467_2026_77989_MOESM14_ESM.pdf]

nature portfolio

Corresponding author(s): Viktória Lázár, Szilvia Juhász

Last updated by author(s): Jun 2, 2026

Reporting Summary

Nature Portfolio wishes to improve the reproducibility of the work that we publish. This form provides structure for consistency and transparency in reporting. For further information on Nature Portfolio policies, see our Editorial Policies and the Editorial Policy Checklist.

Statistics

For all statistical analyses, confirm that the following items are present in the figure legend, table legend, main text, or Methods section.

n/a	Confirmed	
☐	☒	The exact sample size (n) for each experimental group/condition, given as a discrete number and unit of measurement
☐	☒	A statement on whether measurements were taken from distinct samples or whether the same sample was measured repeatedly
☐	☒	The statistical test(s) used AND whether they are one- or two-sided<br>*Only common tests should be described solely by name; describe more complex techniques in the Methods section.*
☒	☐	A description of all covariates tested
☒	☐	A description of any assumptions or corrections, such as tests of normality and adjustment for multiple comparisons
☐	☒	A full description of the statistical parameters including central tendency (e.g. means) or other basic estimates (e.g. regression coefficient) AND variation (e.g. standard deviation) or associated estimates of uncertainty (e.g. confidence intervals)
☐	☒	For null hypothesis testing, the test statistic (e.g. F, t, r) with confidence intervals, effect sizes, degrees of freedom and P value noted<br>*Give P values as exact values whenever suitable.*
☒	☐	For Bayesian analysis, information on the choice of priors and Markov chain Monte Carlo settings
☒	☐	For hierarchical and complex designs, identification of the appropriate level for tests and full reporting of outcomes
☐	☒	Estimates of effect sizes (e.g. Cohen's d, Pearson's r), indicating how they were calculated

Our web collection on statistics for biologists contains articles on many of the points above.

Software and code

Policy information about availability of computer code

Data collection	We used Harmony to operate the PerkinElmer Operetta used for data collection. No analytic modules were used - output images were exported for further analysis.
Data analysis	We used BIAS (version 2024.02.12.) to analyse the images and acquire single-cell data. Afterwards, we have used a custom R script for analysis.

For manuscripts utilizing custom algorithms or software that are central to the research but not yet described in published literature, software must be made available to editors and reviewers. We strongly encourage code deposition in a community repository (e.g. GitHub). See the Nature Portfolio guidelines for submitting code & software for further information.

Data

Policy information about availability of data

All manuscripts must include a data availability statement. This statement should provide the following information, where applicable:

- Accession codes, unique identifiers, or web links for publicly available datasets
- A description of any restrictions on data availability
- For clinical datasets or third party data, please ensure that the statement adheres to our policy

The raw data generated in this study, including all microscopy images and microplate images of bacterial colonies, are be deposited in the HUN-REN ARP public data repository in the https://repo.researchdata.hu/dataverse/malvinamethod dataverse under accesssion links https://doi.org/21.15109/ARP/23FTBV and https://doi.org/21.15109/ARP/YORL70 for download. Raw sequencing data and genome assemblies generated as part of this work have been deposited in the European

Nucleotide Archive (ENA) under Study Accession number PRJEB120792.

Research involving human participants, their data, or biological material

Policy information about studies with human participants or human data. See also policy information about sex, gender (identity/presentation), and sexual orientation and race, ethnicity and racism.

Reporting on sex and gender	N/A
Reporting on race, ethnicity, or other socially relevant groupings	N/A
Population characteristics	N/A
Recruitment	N/A
Ethics oversight	N/A

Note that full information on the approval of the study protocol must also be provided in the manuscript.

Field-specific reporting

Please select the one below that is the best fit for your research. If you are not sure, read the appropriate sections before making your selection.

[x] Life sciences [ ] Behavioural & social sciences [ ] Ecological, evolutionary & environmental sciences

For a reference copy of the document with all sections, see nature.com/documents/nr-reporting-summary-flat.pdf

Life sciences study design

All studies must disclose on these points even when the disclosure is negative.

Sample size	For all conditions tested, we measured using three biological replicates. To determine sample size adequacy, we compared the biologically connected measurements to random noise and random groupings, using Kolgomorov-Smirnov testing to check the distribution characteristics. We found that the biologically connected measurements (same compounds, same bacterial strains) were more connected than random groupings and the datapoints were thus adequately describing the conditions tested.
Data exclusions	Data was only excluded in cases where an individual sample's Z-score exceeded a three-fold difference, signifying a quantifiable deviation compared to other measurements; see Methods
Replication	Our high-throughput virulence was highly reproducible, showing good agreement among the three biological replicates.
Randomization	The plate allocation for the compounds were chosen at random. The replicate measurements were made independently, from distinct aliquots, cell passages, and overnight cultures.
Blinding	The virulence metrics in the presence of different compounds and/or bacterial strains were analyzed in the same way, while blind to the compound identity of each well.

Reporting for specific materials, systems and methods

We require information from authors about some types of materials, experimental systems and methods used in many studies. Here, indicate whether each material, system or method listed is relevant to your study. If you are not sure if a list item applies to your research, read the appropriate section before selecting a response.

Materials & experimental systems

n/a	Involved in the study	
[ ]	[x]	Antibodies
[ ]	[x]	Eukaryotic cell lines
[x]	[ ]	Palaeontology and archaeology
[x]	[ ]	Animals and other organisms
[x]	[ ]	Clinical data
[x]	[ ]	Dual use research of concern
[x]	[ ]	Plants

Methods

n/a	Involved in the study	
[x]	[ ]	ChIP-seq
[x]	[ ]	Flow cytometry
[x]	[ ]	MRI-based neuroimaging

Antibodies

Antibodies used	γH2AX, ZO1
Validation	Commercially available; see Methods

Eukaryotic cell lines

Policy information about cell lines and Sex and Gender in Research

Cell line source(s)	CaCo2
Authentication	Cell lines were acquired from a cell bank, which were authenticated before purchase
Mycoplasma contamination	Contamination was tested - no contamination was observed
Commonly misidentified lines (See ICLAC register)	-

Plants

Seed stocks	-
Novel plant genotypes	-
Authentication	-
