# Supplementary material for: Deep-learning single-cell profiling uncovers interbacterial and drug-driven reshaping of pathogen virulence
Source: Nat Commun. 2026 Sep 29;17:11236. doi: 10.1038/s41467-026-77989-w (PMC13720132; doi:10.1038/s41467-026-77989-w)
Supplement: Supplementary file 2 [file 41467_2026_77989_MOESM15_ESM.pdf]

nature portfolio

Peer Review File

Deep-learning single-cell profiling uncovers interbacterial and drug-driven reshaping of pathogen virulence

Corresponding Author: Dr Viktoria Lazar

This file contains all reviewer reports in order by version, followed by all author rebuttals in order by version.

Version 0:

Reviewer comments:

Reviewer #1

(Remarks to the Author)
This paper presents an image-based high-throughput screening approach to map bacterial infections in cultured cells, and investigate similarities and differences in infection load and genotoxicity by the use of two fluorescent markers.

The interesting part of the result is more on the biological side than on the computational side. The computational approach consists of image analysis as well as more in-depth analysis of the image analysis output. The most interesting novelty on the image analysis side seems to lie in the automated filtering of artefacts such as out-of-focus cells and extracellular bacterial aggregates. The most interesting finding is antimicrobials that seem to promote bacterial invasion.

In general, the paper is well-written and the findings are interesting. There are however some clarity when it comes to generalizability and choice of methods that could strengthen the paper.

It is stated that for both data and code, 'A link and access credentials have been provided to the referees for review purposes.' This does however not seem to be the case, and therefore limits the possibilities of doing a complete review. There is no password provided for https://cloud.hcemm.eu/

Concerns:
It is claimed that the proposed workflow MALVINA offers a generalizable framework for dissecting pathogen-host-drug interactions. Such generalizability is however not shown in the paper, e.g. using data from other labs etc. The presented work seems to be limited in its uniqueness from an image analysis point of view as co-culture of human cells and bacteria for the study of virulence is not novel. See e.g.

https://pmc.ncbi.nlm.nih.gov/articles/PMC3421616/
https://elifesciences.org/articles/97495

The analysis approach is based on more advanced (previously published) segmentation of individual cell nuclei followed by very simple expansion to define regions surrounding the nuclei. This is followed by extracting intensity and texture features across imaging channels within these regions. This can be regarded as standard procedures for any image-based screening today. The fact that the fluorescent stains represent genotoxicity and infection load does not change the analysis approach. The presented MALVINA pipeline builds on an in-house software, and no comparison is done to analysis based on more established open-source software, such as e.g. CellProfiler. I do not believe such a comparison should be absolutely required, but a discussion on novel functionality in MALVIA would be useful.

I would however want to see more motivation for using a learning-based approach for cell classification. Since the cells are simply classified as infected or non-infected, one wonders why both Hoechst and mScarlet stain, as well as a number of Haralick textures, integrated, maximal, mean, minimal, and median intensities would be required, in combination with machine learning. Why is e.g. integrated intensity o f mScarlet not sufficient? A 10% cross validation is claimed to reach an accuracy of 86% - what would the accuracy be with a simple classification based on one or two features, such as mean and standard deviation of mScarlet intensity?

It is nice to see that there are multiple replicates in all presented experiments. The approaches for normalization are also described in detail. It would therefore be beneficial if data points from replicates could be presented separately so better communicate variability across replicates.

A description of computational resources seems to be lacking.

Over all, the paper will inspire others to do similar screens of co-cultured human and bacterial cells, and as such the methods and presented results are well worth publishing.

(Remarks on code availability)
There is no password provided for https://cloud.hcemm.eu/

Reviewer #2

(Remarks to the Author)
In this manuscript, Bognár and co-workers have used a microscopy and image analysis approach to quantify the abundance and distribution of intracellular E. coli and host cell DNA damage. They use this approach to determine how different strains individually and in combination affect invasion. They also measure how drug treatments alter invasion, finding that 16 out of 23 drugs tested altered bacterial invasion parameters. This is a very interesting observation highlighting the potential collateral effects of these compounds. I have two general comments 1) The assay developed cannot distinguish between alteration in invasion due to the effect of the drug on the bacteria or on the host cells. This should be made clearer in the text, or, ideally, other controls should be used to distinguish these two possibilities. 2) The model for bacterial invasion here is colorectal epithelial cells. The mucus layer is an absolutely critical part of protection from pathogen invasion in the intestine. They state that these cells can produce mucus, but do not check this or look at how treatments alter this. Please see below for a complete list of my comments:

Comments:

Page 84: some more introduction to colibactin and other virulence mechanism would be useful.

149: Give details on virulence of the specific E. coli strains used – which virulence factor are encoded (beyond colibactin) and how might these be expected to alter invasion etc.

Line 164: what is the definition of hyper-invasive here? This strain is more commonly called adherent-invasive.

Figure 2,c: the CCR20 cells show a subpopulation with decreased genotoxicity – what is happening here?

Figure 2.d: please just write the name of the strain rather than the symbol key.

Line 210: This density dependence would be interesting to test more systematically.

Line 231: how were these compounds chosen?

Line 236: The intracellular concentration of the drugs will likely be very different to the extracellular concentrations used, which means discriminating between virulence inhibition (intracellular) and direct drug effects (intracellular) base on extracellular measurements are not straightforward. How do the authors account of this?

Line 248: how can you be sure this is increased virulence and not drugs affecting host cells, decreasing host defences or increasing permeability to bacteria? We know that trimethoprim for example, can perturb folate metabolism in humans (hence its contraindication in pregnancy)? Either controls are needed to rule out host cell effects, or the wording throughout the paper (and title) need to reflect that it is unclear that these are directly affecting bacterial virulence.

Line 250: as above NTR presumably strongly perturbs host cells, which could be the cause increased invasion, rather than and direct effect on virulence. This should be made clear in the text.

Line 253 the authors speculate that the effect of omadacycline and doxcyleine (antibiotics) could be epithelial tightening, e.g. an effect on host cells. What is the evidence for this? Why do they not consider host cell effects for the invasion enhancing drugs?

Figure 5: It would be helpful to have an x axis labels on panel e, f. Perhaps naming the groupings from the previous panels.

Line 254: I'm not clear to me from the methods on if wash steps are included when giving drugs to test invasion and when using gentamycin to kill extracellular bacteria. Is the doxycycline still present when gentamycin is used? If so, these are highly antagonistic and so the combination would lead to not eradicating the extracellular bacteria, which could go on to invade if not eradicated.

Line 450: the mucus layer is critical for blocking invasion, but will depend on the exact in vitro growth conditions for these cells. Please check mucus production in your setup. It would be interesting to test the effect of the drugs, strains on the host mucus production and how this alters invasion.

Line 559: A general comment on the high content imaging; Having read the methods the actual metrics used are rather simple: essentially mean fluorescence of the host cell. I'm not sure that really justifies calling this 'high content'. The example cells shown in Fig. 2 suggests that there is a lot more content - intracellular spatial distribution etc, that could be extracted. In the example cells the DNA damage maker seems to show a lot of variation beyond mean intensity.

Line 632: Infection is based on mean fluorescence: what was the cut-off of non-infected vs infected and how was it determined?

(Remarks on code availability)

Version 1:

Reviewer comments:

Reviewer #2

(Remarks to the Author)
The authors have done a good job addressing the reviewer comments and improving the clarity of the manuscript.

(Remarks on code availability)

Open Access This Peer Review File is licensed under a Creative Commons Attribution 4.0 International License, which permits use, sharing, adaptation, distribution and reproduction in any medium or format, as long as you give appropriate credit to the original author(s) and the source, provide a link to the Creative Commons license, and indicate if changes were made.
In cases where reviewers are anonymous, credit should be given to 'Anonymous Referee' and the source.
The images or other third party material in this Peer Review File are included in the article's Creative Commons license, unless indicated otherwise in a credit line to the material. If material is not included in the article's Creative Commons license and your intended use is not permitted by statutory regulation or exceeds the permitted use, you will need to obtain permission directly from the copyright holder.
To view a copy of this license, visit https://creativecommons.org/licenses/by/4.0/

Response to Reviewers

Manuscript: Deep-learning single-cell profiling uncovers interbacterial and drug-driven reshaping of pathogen virulence

REVIEWER COMMENTS

Reviewer #1 (Remarks to the Author): This paper presents an image-based high-throughput screening approach to map bacterial infections in cultured cells, and investigate similarities and differences in infection load and genotoxicity by the use of two fluorescent markers.

The interesting part of the result is more on the biological side than on the computational side. The computational approach consists of image analysis as well as more in-depth analysis of the image analysis output. The most interesting novelty on the image analysis side seems to lie in the automated filtering of artefacts such as out-of-focus cells and extracellular bacterial aggregates. The most interesting finding is antimicrobials that seem to promote bacterial invasion.

In general, the paper is well-written and the findings are interesting. There are however some clarity when it comes to generalizability and choice of methods that could strengthen the paper.

We thank Reviewer #1 for the thoughtful and constructive evaluation of our manuscript and for recognising the biological relevance of the findings enabled by the MALVINA framework. We appreciate the reviewer's comments regarding methodological clarity, positioning of novelty, and generalizability, and we have revised the manuscript extensively to address these points. Below we respond to each comment in detail.

It is stated that for both data and code, 'A link and access credentials have been provided to the referees for review purposes.' This does however not seem to be the case, and therefore limits the possibilities of doing a complete review. There is no password provided for https://cloud.hcemm.eu/

We apologise for the oversight. Access credentials have now been provided not only in the previous Data Availability document and in the submission interface, but also added to the revised Data Availability statement and included below for convenience:

Data availability: https://cloud.hcemm.eu/
Username: malvina
Password: #MLVNA2025!

Concerns:

It is claimed that the proposed workflow MALVINA offers a generalizable framework for dissecting pathogen-host-drug interactions. Such generalizability is however not shown in the paper, e.g. using data from other labs etc.

We thank the Reviewer for this important comment. While external datasets were not available in a directly compatible format for MALVINA analysis, we addressed the generalizability concern experimentally by extending the workflow to an independent infection model beyond the original Caco-2/*E. coli* model, involving clinical *Klebsiella pneumoniae* isolates and human lung epithelial A549 adenocarcinoma cells. Because of the distinct morphology and staining characteristics of A549 cells, we additionally trained a new infection classification model for this host-cell background (Methods, Extended Data figure 3, Supplementary Table 2). These experiments demonstrated that the workflow can be readily adapted across distinct human cell types and bacterial pathogens while retaining robust invasion and genotoxicity profiling capability. We have now included these additional experiments in the revised manuscript (Extended Data Figure 3 and corresponding Results section "*Comparative Characterisation of Pathogen Invasiveness and Genotoxicity*").

The presented work seems to be limited in its uniqueness from an image analysis point of view as co-culture of human cells and bacteria for the study of virulence is not novel. See e.g

https://pmc.ncbi.nlm.nih.gov/articles/PMC3421616/
https://elifesciences.org/articles/97495

We thank the reviewer for this important comment and for pointing us to relevant prior works. We fully agree that host-pathogen co-culture assays, high-content microscopy, and machine-learning-powered image analysis have been successfully applied to infection biology.

The studies highlighted by the reviewer address related but distinct image-analysis problems. For example, Osaka et al. developed an automated CellProfiler workflow for enumeration of *Chlamydia* inclusions and screening of antichlamydial agents. Similarly, López-Jiménez et al. used high-content microscopy and CNN-based image analysis to dissect *Shigella* infection heterogeneity and host-pathogen interaction phenotypes at single-cell resolution. These studies established the value of automated microscopy approaches for host-pathogen investigations.

The novelty of MALVINA lies in the development of an integrated, high-throughput, single-cell virulence-profiling framework that resolves bacterial pathogenicity into distinct phenotypic dimensions, including invasion width, invasion depth, direct genotoxicity in infected cells, and population-level genotoxicity in uninfected cells. This enables simultaneous quantification of intracellular bacterial burden and γH2AX-marked host DNA damage at single-cell resolution. To our knowledge, the cited approaches do not combine intracellular bacterial burden and host DNA damage into a unified single-cell assay, nor do they use this coupled readout to map interbacterial virulence modulation or drug-driven reshaping of both invasion and genotoxicity.

From an image-analysis perspective, MALVINA therefore contributes a task-specific analytical workflow rather than a generic new neural-network architecture. The pipeline integrates host-cell segmentation, intracellular bacterial signal quantification, infected/non-infected cell classification, exclusion of adherent or extracellular bacterial artefacts, and extraction of matched bacterial-load and γH2AX features from the same individual host cells. This design was essential for addressing the biological questions studied here. It enabled (1) systematic characterization of virulence phenotypes across bacterial strains, (2) distinction between bacterial toxin-associated genotoxicity and infection-associated DNA damage within invaded host cells, (3) quantification of how bacterial competitors reshape the virulence phenotypes of co-infecting strains, and (4) identification of drug-induced shifts in coupled invasion and genotoxicity phenotypes. In addition, MALVINA introduces analytical concepts such as the "genotoxicity shift", enabling discrimination between host DNA damage arising from drug toxicity or bacterial clearance and changes associated with direct modulation of bacteria-associated genotoxic mechanisms.

We have revised the Introduction to acknowledge these prior automated, high-content imaging host–pathogen studies.

The analysis approach is based on more advanced (previously published) segmentation of individual cell nuclei followed by very simple expansion to define regions surrounding the nuclei. This is followed by extracting intensity and texture features across imaging channels within these regions. This can be regarded as standard procedures for any image-based screening today. The fact that the fluorescent stains represent genotoxicity and infection load does not change the analysis approach. The presented MALVINA pipeline builds on an in-house software, and no comparison is done to analysis based on more established open-source software, such as e.g. CellProfiler. I do not believe such a comparison should be absolutely required, but a discussion on novel functionality in MALVINA would be useful.

Thank you for your comment and for raising this important point.

BIAS is not an in-house research tool developed specifically for this project, but a commercially available image-analysis platform developed by SingleCell Technologies Ltd. The software has been used in numerous peer-reviewed studies across a broad range of biological applications, demonstrating its robustness and suitability for high-content microscopy workflows. A publication list and trial versions of the software are available at https://sct.bio/publication_list/.

Our choice of BIAS was primarily motivated by its broad compatibility with microscopy platforms and its ability to directly import native image formats from multiple instrument manufacturers while preserving associated metadata. This is achieved through commercial deals between SingleCell Technologies Ltd. and brands such as Leica, Revvity (formerly PerkinElmer), Zeiss, GE, Olympus, etc. This eliminates the need for intermediate file conversion steps and facilitates the handling of large high-content imaging datasets.

Importantly, the image-analysis workflow presented in this study does not rely on functionality unique to BIAS. The segmentation, feature extraction, and machine-learning procedures could in principle be implemented in other image-analysis environments, including CellProfiler. We

therefore do not claim that the biological conclusions depend on the use of a particular software package.

To facilitate reproducibility, we have made the complete analysis pipelines developed for this study available in the shared data repository together with a trial version of the software. These pipelines can be executed using the trial version, which differs from the full commercial release only by the absence of GPU acceleration and selected developmental features.

We have also revised the manuscript to better describe the features of BIAS that motivated its use in this study (*Computational resources used*).

I would however want to see more motivation for using a learning-based approach for cell classification. Since the cells are simply classified as infected or non-infected, one wonders why both Hoechst and mScarlet stain, as well as a number of Haralick textures, integrated, maximal, mean, minimal, and median intensities would be required, in combination with machine learning. Why is e.g. integrated intensity o f mScarlet not sufficient? A 10% cross validation is claimed to reach an accuracy of 86% - what would the accuracy be with a simple classification based on one or two features, such as mean and standard deviation of mScarlet intensity?

We appreciate raising this important point. The cells are not only classified as infected or non-infected, since in certain situations, adherent biofilms or other artefacts can make determining infection impossible. Thus, we classify cells into three categories: infected, noninfected, and non-usable (see Methods: *Machine learning model for infection classification*; Extended data figure 1). Haralick textures, originally developed for classification applications, were an existential component in the determination of infected vs non-usable cells.

Using only the reviewer's suggested two metrics in a model with the same architecture, training and validation datasets as the original showed a marked decrease in accuracy. For example, noninfected cells had an elevated false positive rate (33.3% compared to 10.4%) with a true negative rate of 66.66% (compared to 89.6%), while infected cells had a true positive rate of 47.56% (compared to 82.54%).

We turned the former Extended Data Figure 10 presenting model accuracies into a Supplementary Table 2 showing these changes as well as the original model accuracies. We have added a line discussing this topic in Methods: *Machine learning model for infection classification.*

It is nice to see that there are multiple replicates in all presented experiments. The approaches for normalization are also described in detail. It would therefore be beneficial if data points from replicates could be presented separately so better communicate variability across replicates.

Thank you for your comment. We have created interactive, standalone html files showing the replicates of each experiment. These files can be opened in any browser, and make it possible for example to view the results per well in one experiment, or only one treatment condition across all experiments. These files are now included as supplementary files. We have also updated the code repository to include all code involved in the creation of these files.

A description of computational resources seems to be lacking. Overall, the paper will inspire others to do similar screens of co-cultured human and bacterial cells, and as such the methods and presented results are well worth publishing.

Thank you for your assessment and comment. For this reason, we have added a *Computational resources used* section in Methods, outlining the machine we used for analysis in greater detail.

Reviewer #1 (Remarks on code availability): There is no password provided for https://cloud.hcemm.eu/

We apologise for the oversight. Access credentials have now been provided not only in the previous Data Availability document and in the submission interface, but also added to the revised Data Availability statement and included below for convenience:

Data availability: https://cloud.hcemm.eu/
Username: malvina
Password: #MLVNA2025!

Reviewer #2 (Remarks to the Author):

In this manuscript, Bognár and co-workers have used a microscopy and image analysis approach to quantify the abundance and distribution of intracellular E. coli and host cell DNA damage. They use this approach to determine how different strains individually and in combination affect invasion. They also measure how drug treatments alter invasion, finding that 16 out of 23 drugs tested altered bacterial invasion parameters. This is a very interesting observation highlighting the potential collateral effects of these compounds. I have two general comments:

We thank Reviewer#2 for the constructive and mechanistically insightful comments. We agree that the Reviewer's request for clarification regarding host-versus-bacterial effects, epithelial barrier integrity, and host-cell susceptibility substantially improved both the clarity of the manuscript and the interpretation of the results. In particular, the additional control experiments performed in response to these comments helped refine our interpretation of the observed drug-induced invasion phenotypes and substantially improved the manuscript.

1) The assay developed cannot distinguish between alteration in invasion due to the effect of the drug on the bacteria or on the host cells. This should be made clearer in the text, or, ideally, other controls should be used to distinguish these two possibilities.

Thank you for this important comment. We agree that distinguishing whether drug-induced invasion phenotypes arise primarily from effects on bacteria, host cells, or their interaction is critical for interpreting the results.

To address this concern, we performed additional follow-up control experiments using selected compounds with the strongest invasion-modulating effects (TRM, GPC, OMA and NTR). In these assays, either bacteria or host cells were pre-treated separately with the compounds,

followed by washout prior to infection. We additionally assessed the effects of these drugs on epithelial barrier integrity and host-cell membrane damage using transepithelial electrical resistance (TEER) and SytoxBlue accumulation assays.

These experiments revealed that:
(i) host-cell pre-treatment alone for 4 hours did not enhance invasion by either tested pathogen (LF82 or CCR20) and instead resulted in a modest global reduction across all tested conditions (Extended Figure 7e,f);
(ii) short 1.5-hour drug pre-exposure of either CCR20 or LF82 strains prior to infection induced only subtle increases in invasion width and/or invasion depth under selected conditions (Extended Figure 7g,h); and
(iii) TEER and SytoxBlue measurements performed during drug treatment and up to 24 hours thereafter did not indicate major epithelial barrier disruption or general host-cell damage as explanations for the observed drug-induced invasion phenotypes (Extended Figure 7a-d).

Importantly, the strongest invasion-modulating effects were consistently observed only under co-treatment conditions (Figure 5), where drugs were present during infection. Together, these findings argue against a simple explanation based solely on impaired host-cell barrier integrity or reduced host defence and are more consistent with the interpretation that the observed phenotypes emerge from dynamic host-pathogen-drug interactions.

We have incorporated these new control experiments and their interpretation into the revised Results (*Profiling drug-induced modulation of infection phenotypes*, Extended Data Figure 7) and Discussion.

2) The model for bacterial invasion here is colorectal epithelial cells. The mucus layer is an absolutely critical part of protection from pathogen invasion in the intestine. They state that these cells can produce mucus, but do not check this or look at how treatments alter this. Please see below for a complete list of my comments:

We thank the Reviewer for this important point. We agree that the mucus layer is a critical component of intestinal barrier function and can substantially influence bacterial invasion dynamics. In response to this comment, we examined mucus production in our experimental system by assessing MUC2 staining in the Caco-2 monolayers used throughout the study. Under our experimental conditions, MUC2 staining in Caco-2 cells was not substantially different from that observed in U2OS osteosarcoma cells, which served as a non-mucosal reference cell line (Extended Data Fig. 2b). These findings are consistent with previous reports showing that conventionally cultured Caco-2 monolayers do not recapitulate a mature mucus-producing intestinal epithelium under standard culture conditions (Floor et al., Gut Microbes, 2024). We have now clarified this point in the revised Methods section and explicitly state that our experimental system primarily captures direct epithelial-bacterial interactions rather than infection dynamics shaped by a mature mucus layer.

Comments:

Page 84: some more introduction to colibactin and other virulence mechanisms would be useful.

Thank you for this comment. We expanded the Introduction section to provide additional background on colibactin and other virulence-associated mechanisms contributing to bacterial invasion and genotoxicity.

149: Give details on virulence of the specific E. coli strains used – which virulence factor are encoded (beyond colibactin) and how might these be expected to alter invasion etc.

We thank the Reviewer for this helpful suggestion. In response, we performed a comparative virulence factor analysis of all four *E. coli* strains using high-confidence sequence similarity searches with BLASTP against the VFDB database [DOI: 10.1093/nar/gkae968] and incorporated these results into the revised manuscript (Supplementary Figure 2-3).

The revised Results section (*Comparative Characterisation of Pathogen Invasiveness and Genotoxicity*) now includes comparative annotation of strain-specific virulence repertoires beyond colibactin production. In particular, we highlight that the invasive clinical isolates encoded expanded host-adaptation and virulence-associated determinants absent from the minimally invasive BW strain, including Chu/Shu heme uptake systems, yersiniabactin-associated iron acquisition loci, the epithelial adhesion factor FdeC, the autotransporter toxin Vat, K1 capsule-associated loci, and T6SS-associated genes in the two most invasive strains (LF82 and CCR20).

We additionally expanded the interbacterial interaction analysis (*Mapping Interbacterial Modulation of Virulence*) by relating the competitive behaviour of the ATCC strain to toxin- and host-modulation-associated determinants uniquely detected in this isolate, including TcpC, hemolysin-associated loci, and the autotransporter toxins Sat and Pic. We also note that the ATCC strain carries an inactivated rpoS allele, which may influence stress adaptation and host-associated behaviour.

Together, these additions provide functional genetic context for the observed invasion and genotoxicity phenotypes and suggest potential contributions of specific virulence-associated determinants to strain-specific invasion and competitive behaviours.

Line 164: what is the definition of hyper-invasive here? This strain is more commonly called adherent-invasive.

We thank the Reviewer for this comment. We agree that LF82 is classically defined as an adherent-invasive *E. coli* (AIEC) strain. In the revised manuscript, we therefore clarify that the term “hyper-invasive” is used specifically to describe strains exhibiting simultaneously high invasion width and invasion depth in our single-cell assay.

The revised text: “Invasion profiling revealed marked strain-specific differences in epithelial invasion behaviour (Figure 2a,c,d; Extended Data Figure 2b-e). LF82 and CCR20 displayed similarly high invasion width and depth, consistent with strongly invasive phenotypes characterised by broad epithelial dissemination and intracellular accumulation. This aligns with the established adherent-invasive E. coli (AIEC) phenotype of LF82 and its association with inflammatory bowel disease[61,62]. Hereafter, LF82 and CCR20 are referred to as “hyper-invasive” strains due to their simultaneously high invasion width and invasion depth.”

This terminology was introduced to distinguish the MALVINA-derived invasion phenotype from the established clinical/pathotype classification of AIEC strains.

Figure 2,c: the CCR20 cells show a subpopulation with decreased genotoxicity – what is happening here?

We thank the Reviewer for this insightful comment. We agree that a small subpopulation of CCR20-containing cells exhibited low γH2AX signal despite intracellular bacterial presence. This observation is biologically plausible, as γH2AX reflects activation of the host-cell DNA damage response rather than merely the physical presence of genotoxic lesions. H2AX phosphorylation is regulated by ATM/ATR/DNA-PK signaling and can therefore display substantial cell-to-cell heterogeneity depending on signaling dynamics, chromatin context, repair kinetics, and cell-cycle state (Bonner et al., 2008, Nat Rev Cancer; Mah et al., 2010, Leukemia; Redon et al., 2010, Clin Cancer Res).

To clarify this point, we have now added a note to the Figure 2 legend stating that a small subpopulation of CCR20-containing cells displayed low genotoxicity signal indicating heterogeneous induction of DNA damage signalling.

Figure 2.d: please just write the name of the strain rather than the symbol key.

We have added the strain name to Figure 2d.

Line 210: This density dependence would be interesting to test more systematically.

We appreciate this suggestion and agree that density-dependent effects on colibactin-associated genotoxicity would be interesting to investigate more systematically. In the present study, infection density was standardised to ensure comparability across single-strain, co-infection and drug-screening experiments, and we did not aim to perform a full inoculum-gradient analysis. Nevertheless, our data include conditions with different effective bacterial densities, including self-mixing, and co-infections with non-genotoxic competitor strains, which showed enhanced ATCC-associated genotoxicity. These observations are consistent with density- or interaction-dependent regulation of colibactin-associated DNA damage and align with previous reports linking colibactin activity to bacterial physiological state and interbacterial interactions (Tang-Fichaux et al., *mSphere* 2020; Wong et al., *PLoS Pathog* 2022). While a systematic mechanistic dissection falls beyond the scope of the current study, MALVINA is well suited for future analyses using controlled inoculum gradients, altered infection time points, and diverse competitor strain combinations.

Line 231: how were these compounds chosen?

We thank the Reviewer for requesting clarification. We have expanded the Methods section (*Infection of human cells with bacteria-* Drug Screening Assays) to better describe the rationale behind compound selection. Briefly, the panel was designed to capture a broad spectrum of pharmacological mechanisms relevant to host-pathogen interactions rather than to represent an exhaustive or clinically prioritised drug set. Compounds were selected to include (i) antibiotics targeting diverse bacterial processes including DNA replication, translation, cell wall synthesis, and membrane integrity; (ii) antimicrobial peptides and biocides inducing distinct bacterial stress responses; and (iii) chemotherapeutic and DNA-damaging agents known to affect host-cell physiology. This diversity enabled systematic assessment of how

mechanistically distinct perturbations influence bacterial invasion and genotoxicity phenotypes within a unified experimental framework.

Line 236: The intracellular concentration of the drugs will likely be very different to the extracellular concentrations used, which means discriminating between virulence inhibition (intracellular) and direct drug effects (intracellular) based on extracellular measurements are not straightforward. How do the authors account for this?

We thank the Reviewer for pointing this out. We agree that the previous wording was prone to misunderstanding. Drug effects on extracellular bacterial survival do not reflect intracellular drug exposure, and intracellular virulence inhibition cannot be directly inferred from these measurements. Rather, extracellular bacterial counts were quantified following the infection phase in the presence of the drugs to assess whether changes in invasion or genotoxicity could simply be explained by reduced numbers of viable extracellular bacteria available for host-cell infection.

We clarified this point in the revised manuscript.

Old text: "In parallel, extracellular bacterial counts from drug-treated co-cultures were quantified by colony-forming unit assays to directly assess each compound's bactericidal activity (Figure 1, Methods). This enabled discrimination between reductions in invasion or genotoxicity driven by decreased bacterial load and drug-associated modulation of these phenotypes."

Revised text: "In parallel, extracellular bacterial counts from drug-treated co-cultures were quantified by colony-forming unit assays to directly assess each compound's bactericidal activity (Figure 1, Methods). This enabled assessment of whether reductions in invasion or genotoxicity were attributable to reduced extracellular bacterial viability during infection, rather than to drug-associated modulation of these phenotypes."

Line 248: how can you be sure this is increased virulence and not drugs affecting host cells, decreasing host defences or increasing permeability to bacteria? We know that trimethoprim for example, can perturb folate metabolism in humans (hence its contraindication in pregnancy)? Either controls are needed to rule out host cell effects, or the wording throughout the paper (and title) need to reflect that it is unclear that these are directly affecting bacterial virulence.

Line 250: as above NTR presumably strongly perturbs host cells, which could be the cause increased invasion, rather than and direct effect on virulence. This should be made clear in the text.

We thank the Reviewer for these important comments. As discussed in response to Comment 1, we agree that drug-induced changes in bacterial invasion could theoretically arise from altered bacterial behaviour, altered host-cell susceptibility or epithelial permeability, or both. This consideration is particularly relevant for compounds such as trimethoprim (TRM) and N-nitroso-N-ethylurea (NTR), which can also affect host-cell physiology. To address this, we performed additional control experiments using representative invasion-modulating compounds, including TRM, gepotidacin (GPC), omadacycline (OMA), and NTR.

Importantly, host-cell 4-hour drug pre-treatment alone did not increase invasion of either LF82 or CCR20 and instead caused a modest overall reduction in invasion metrics (Extended Data Figure 7c,d). In addition, transepithelial electrical resistance (TEER) and SytoxBlue assays did not indicate significant epithelial barrier disruption or general host-cell damage under the relatively short drug exposure conditions used in our assay (Extended Data Figure 7e,f). The strongest invasion-promoting phenotypes were consistently observed only during co-treatment conditions, where bacteria, host cells, and drugs were present simultaneously during infection (Figure 5).

Together, these findings argue against a simple explanation based solely on impaired host-cell barrier integrity or reduced host defence and are more consistent with the interpretation that the observed phenotypes emerge from host-pathogen-drug interactions.

We incorporated these additional control experiments and their interpretation into the revised Results and Discussion sections.

Line 253 the authors speculate that the effect of omadacycline and doxcyleine (antibiotics) could be epithelial tightening, e.g. an effect on host cells. What is the evidence for this? Why do they not consider host cell effects for the invasion enhancing drugs?

We thank the Reviewer for this important comment. We agree that our previous speculation regarding possible "epithelial tightening" effects of omadacycline (OMA) and doxycycline (DOX) on host cells was not sufficiently supported by direct experimental evidence. We have therefore removed this interpretation from the revised manuscript.

Importantly, the additional control experiments performed in response to the Reviewer's comments did not support a major host-cell barrier tightening mechanism under the tested conditions. Transepithelial electrical resistance (TEER) measurements following 4-hour drug treatment did not show significant differences compared to untreated controls. Moreover, host-cell pre-treatment alone with omadacycline resulted only in a modest reduction in invasion metrics, a trend that was similarly observed with several other tested compounds. Likewise, short bacterial pre-exposure to omadacycline prior to infection induced only limited effects and did not recapitulate the invasion-modulating phenotypes observed during co-treatment conditions. In contrast, the strongest invasion-modulating effects of omadacycline were consistently observed during co-treatment conditions, in which bacteria, host cells, and drugs were present simultaneously during the infection phase.

We therefore agree that the observed phenotypes likely emerge from complex host-pathogen-drug interactions rather than from exclusively host-cell-driven effects. Accordingly, we revised the manuscript text to avoid overinterpretation of the underlying mechanism.

Line 254: I'm not clear to me from the methods on if wash steps are included when giving drugs to test invasion and when using gentamycin to kill extracellular bacteria. Is the doxycycline still present when gentamycin is used? If so, these are highly antagonistic and so the combination would lead to not eradicating the extracellular bacteria, which could go on to invade if not eradicated.

We thank the Reviewer for highlighting the need to clarify this methodological detail. We agree that the previous Methods description did not sufficiently clarify this. In our assay, the tested compounds were present only during the initial 3-hour infection phase together with the bacteria and host cells. After this co-incubation period, the drug-containing infection

supernatants were collected for extracellular bacterial quantification and removed prior to gentamicin treatment. The monolayers were then incubated in fresh gentamicin-containing human cell culture medium without the tested compounds to eliminate extracellular bacteria. Thus, doxycycline or other tested drugs were not present during the gentamicin treatment step, and potential antagonistic interactions between doxycycline and gentamicin could not affect extracellular bacterial eradication in our assay. We have clarified these experimental steps in the revised Methods section.

Figure 5: It would be helpful to have an x axis labels on panel e, f. Perhaps naming the groupings from the previous panels.

We corrected Figure 5 accordingly.

Line 450: the mucus layer is critical for blocking invasion, but will depend on the exact in vitro growth conditions for these cells. Please check mucus production in your setup. It would be interesting to test the effect of the drugs, strains on the host mucus production and how this alters invasion.

We thank the Reviewer for this important point. We agree that the mucus layer is a critical component of intestinal barrier function and can substantially influence bacterial invasion dynamics. In response to this comment, we examined MUC2 expression in the Caco-2 monolayers used throughout the study. Under our experimental conditions, MUC2 staining was minimal and comparable to that observed in U2OS osteosarcoma cells, which served as a non-mucosal reference cell line (Extended Data Fig. 2b). These findings indicate limited mucus production in our experimental system and are consistent with previous reports showing that conventionally cultured Caco-2 monolayers do not recapitulate a mature mucus-producing intestinal epithelium under standard culture conditions (Floor et al., Gut Microbes, 2024). We agree that analysing how bacterial strains or drugs influence mucus production could provide important mechanistic insight into host-pathogen interactions. However, systematic investigation of mucus-dependent invasion dynamics would likely require alternative mucus-producing epithelial models together with dedicated optimisation of culture and staining conditions, which falls beyond the scope of the current study. Nevertheless, we agree that this represents an important direction for future work.

Line 559: A general comment on the high content imaging; Having read the methods the actual metrics used are rather simple: essentially mean fluorescence of the host cell. I'm not sure that really justifies calling this 'high content'. The example cells shown in Fig. 2 suggest that there is a lot more content - intracellular spatial distribution etc, that could be extracted. In the example cells the DNA damage maker seems to show a lot of variation beyond mean intensity.

We thank the Reviewer for this comment and agree that the present analysis focuses on compact, interpretable metrics, including invasion width, invasion depth (mean integrated intracellular bacterial signal), and population-level and direct genotoxicity (mean integrated γH2AX-associated signal). However, MALVINA retains richer high-content imaging information, including texture and morphology. These features play an important role in the analysis by supporting machine-learning-based infection classification and artefact exclusion in the current study (see also response to Reviewer #1 regarding infection classification and Supplementary Table 2). Future studies could further exploit these features to analyse intracellular bacterial distribution or heterogeneous γH2AX patterns.

Line 632: Infection is based on mean fluorescence: what was the cut-off of non-infected vs infected and how was it determined?

We thank the reviewer for raising this point and welcome the opportunity to clarify our methodology, as there may have been a misunderstanding regarding how infection status was determined.

Infection status was not determined based on mean fluorescence intensity. Instead, cells were classified using a supervised machine-learning approach based on a multilayer perceptron model. The classifier was trained on more than 2,000 manually annotated examples of infected and non-infected cells across multiple independent experiments. In addition, the model was trained to exclude cells for which reliable classification was not possible, including cases with extracellular adherent bacteria that obscured the cell-associated signal (See: *Machine learning model for infection classification* in Methods).

Following classification, each cell was assigned as infected or non-infected, and cells deemed unsuitable for analysis were discarded (See also: *Machine learning model for discarding artefactual cells* in Methods). For cells classified as infected, invasion depth was quantified as the integrated fluorescence signal of the bacterial fluorescent protein. In our analyses, this value is typically presented after log10 transformation, as the resulting distribution is approximately normal on the logarithmic scale.

Thus, infection was not defined by applying a strict fluorescence-intensity cutoff. Rather, infection status was determined by a trained machine-learning classifier based on annotated training data. To avoid bias or data leakage, experiments used for training the classifier were excluded from subsequent downstream analyses.

We also revised the wording in the Methods section for clarity.

Original: "*Invasion depth was defined as the mean intracellular bacterial signal, measured via integrated mScarlet fluorescence, across infected cells in each well, with standard deviation calculated at the single-cell level."*

Revised text: "*Invasion depth was defined as the mean integrated intracellular bacterial signal, measured via mScarlet fluorescence, across infected cells in each well, with standard deviation calculated at the single-cell level."*
