# Supplementary material for: Deep-learning single-cell profiling uncovers interbacterial and drug-driven reshaping of pathogen virulence
Source: Nat Commun. 2026 Sep 29;17:11236. doi: 10.1038/s41467-026-77989-w (PMC13720132; doi:10.1038/s41467-026-77989-w)
Supplement: Supplementary file 3 [file 41467_2026_77989_MOESM1_ESM.pdf]

Supplementary Information

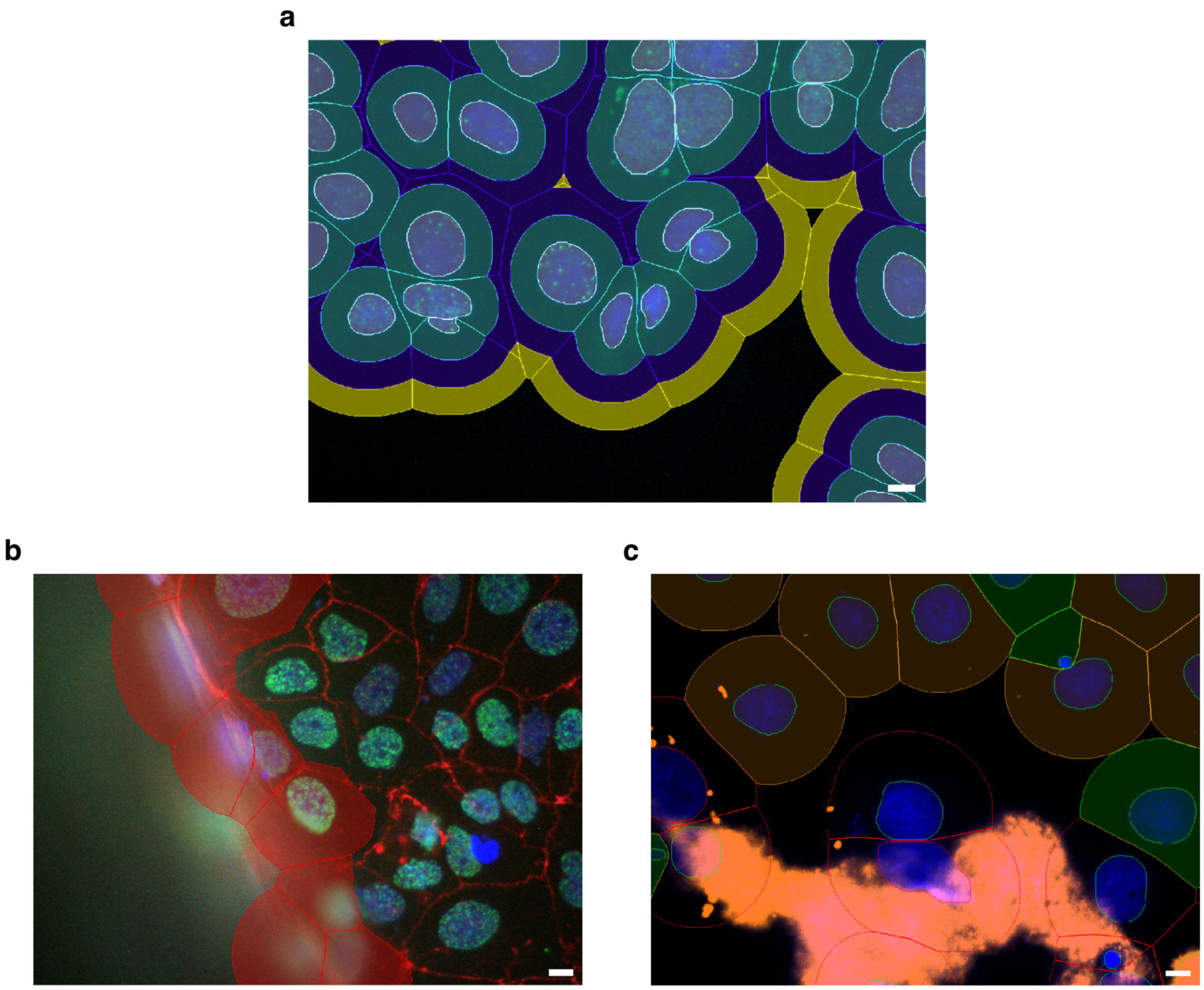

Supplementary Figure 1. Dissection and classification of human cells. a, Cellular segmentation. To approximate whole-cell boundaries, we first applied a deep neural network to segment individual nuclei (white outlines). Based on these nuclear masks, we generated successively dilated regions to approximate the perinuclear space (cyan), distal cytoplasm (blue), and surrounding pericellular interstitial area (yellow). To avoid overlap between adjacent cells, dilation was adaptively halted as neighbouring masks approached each other (Methods). **b**, Exclusion of artefactual cells. To eliminate artefactual cells from downstream analysis, such as detached cells or regions of multilayer growth, a classification step employed a machine learning model trained on all fluorescent channels to identify and remove overlapping or out-of-focus cells (red regions). **c**, Individual cell classification. A machine learning model trained on nuclear and bacterial fluorescence signals classified cells as non-infected (green outlines), truly invaded (orange outlines), or containing surface-adherent bacteria (red outlines). Scale bars represent 10 µm.

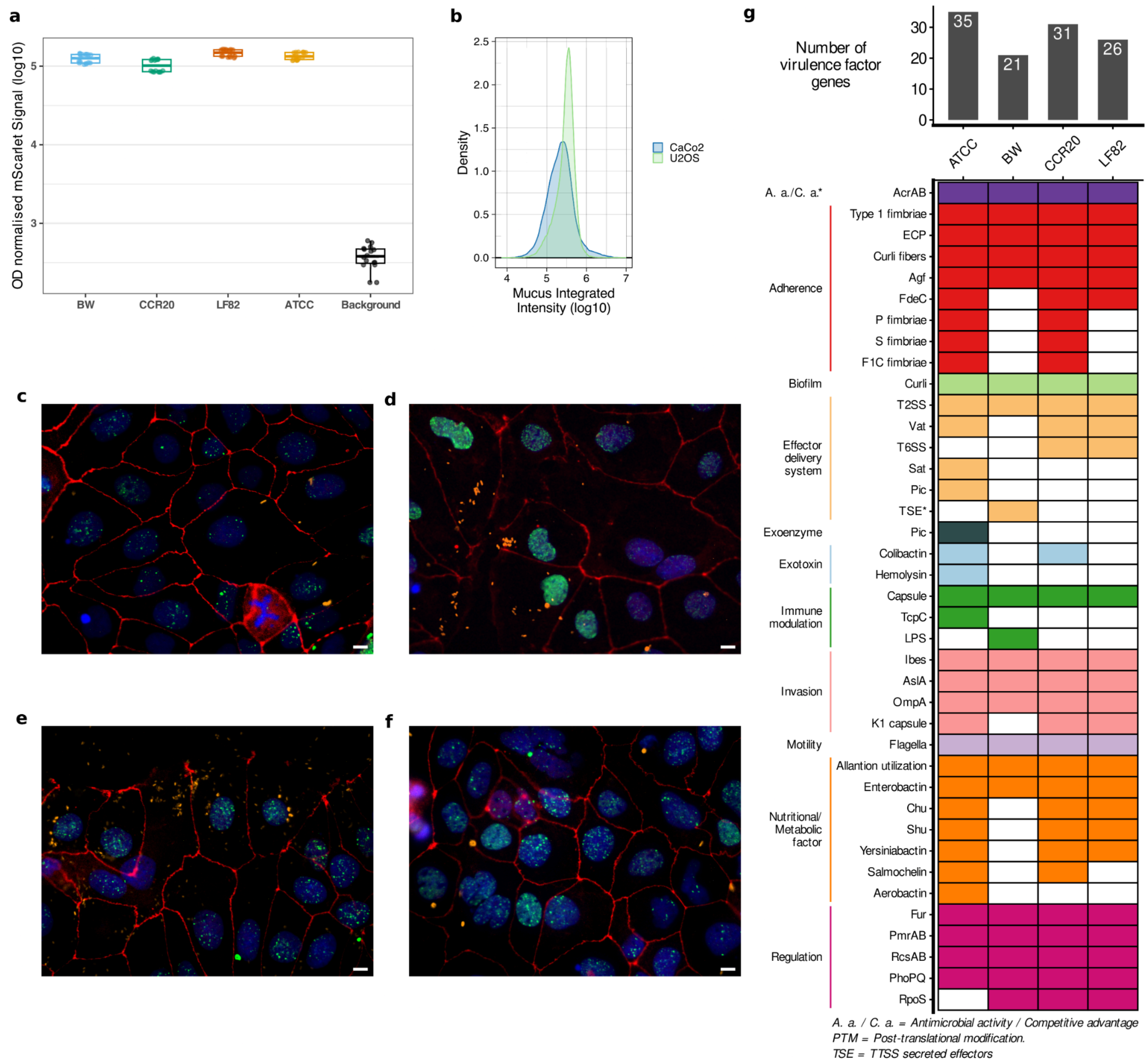

Supplementary Figure 2. *E. coli* strain characteristics. a, Optical density-normalized mScarlet fluorescence signal across *E. coli* strains. Four *E. coli* strains transformed with a plasmid encoding the mScarlet fluorescent protein showed comparable fluorescence signals when measured on a Synergy plate reader (Ex: 565 nm, Em: 593 nm), with only minor variation observed across strains. The maximum difference in fluorescence signal between strains was a 1.42-fold change. Data was derived from two biological replicates, each with at least eight technical replicates. Boxplot hinges represent the first and third quantile, and the upper (lower) whisker extends to the largest (lowest) value no further than 1.5 times the distance between hinges. **b**, Comparison of MUC2 signal with a negative control. To assess mucus production under our culture conditions, MUC2 immunofluorescence signal was quantified in Caco-2 cells and compared with that of the non-mucus-producing U2OS osteosarcoma cell line. MUC2 signal levels in Caco-2 cells were comparable to those observed in the non-mucus-producing U2OS control cell line (Cliff's d = −0.29), indicating the absence of detectable MUC2 accumulation under the culture conditions used in this study. Data was derived from 4 biological replicates, each with at least 10 technical replicates. **c–f**, Representative images of Caco2 colorectal epithelial cancer cells infected with BW (**c**), CCR20 (**d**), LF82 (**e**), and ATCC strain (**f**). Staining corresponds to host nuclei (Hoechst 33342, blue), the host DNA damage marker γH2AX (green), the tight junction protein ZO-1 (red), and mScarlet-labelled bacteria (orange). Scale bars represent 10 µm. **g,** Comparative virulence factor profiles of the four *E. coli* strains.

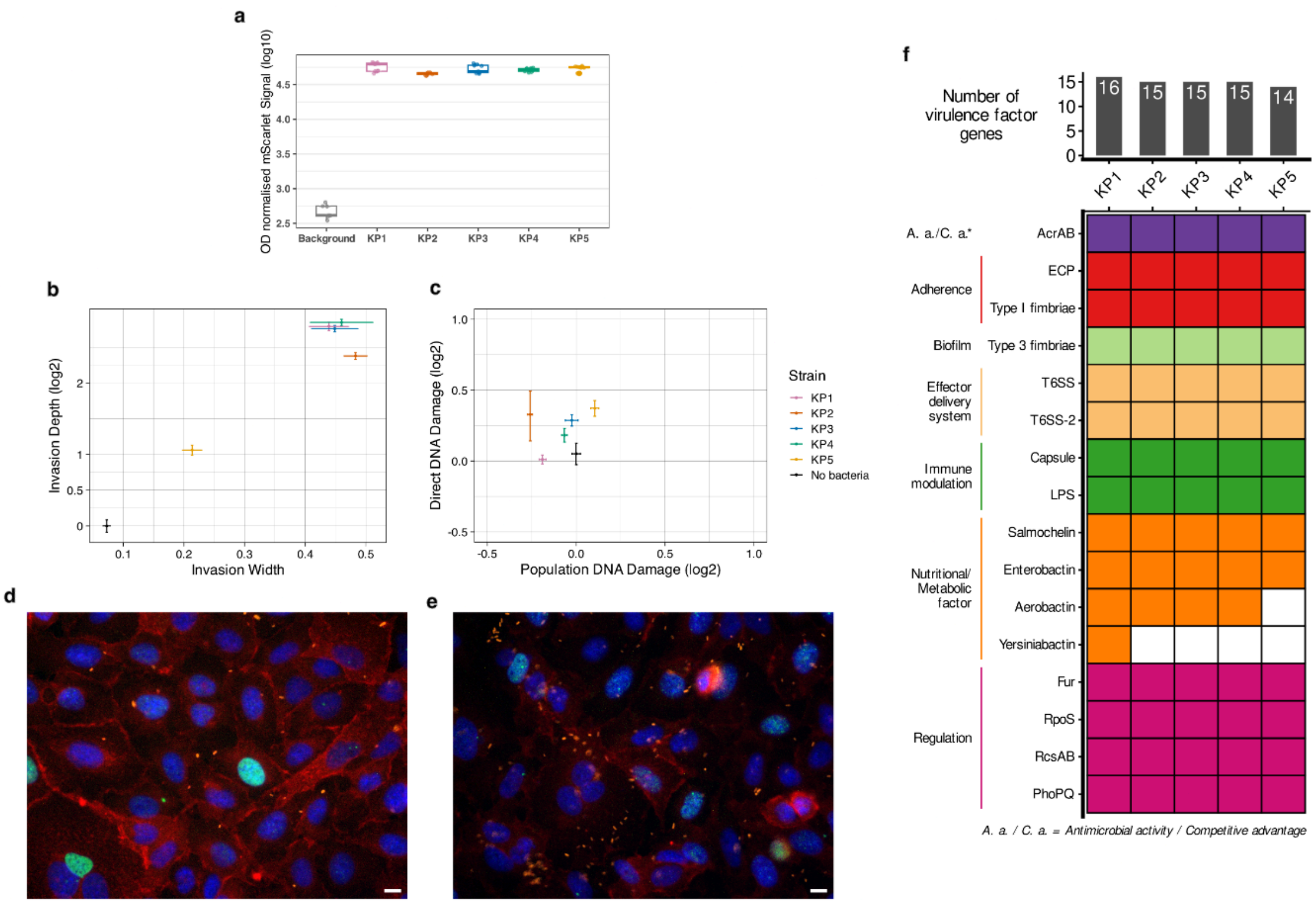

Supplementary Figure 3. Adaptation of MALVINA to lung epithelial A549 cells infected with Klebsiella pneumoniae strains. a, Optical density-normalized mScarlet fluorescence signal across *K. pneumoniae* strains. Five *K. pneumoniae* strains transformed with an mScarlet-expressing plasmid exhibited comparable fluorescence signals when measured using a Synergy plate reader (Ex: 565 nm, Em: 593 nm), with only minor variation observed between strains. The largest difference in fluorescence signal was 1.26-fold. Data were derived from two biological replicates, each comprising at least eight technical replicates. Boxplot hinges represent the first and third quartiles, and whiskers extend to the most extreme values within 1.5× the interquartile range. **b,** Invasion and genotoxicity profile of *K. pneumoniae* strains and the no. Data was derived from 3 biological replicates, each with at least 9 technical replicates. Errors represent s.e.m.. **d,e**, Representative images of A549 lung epithelial cancer cells infected with the KP5 strain (**d**), and the KP3 strain (**e**). Staining corresponds to host nuclei (Hoechst 33342, blue), the host DNA damage marker γH2AX (green), the membrane glucose transporter GLUT1 (red), and mScarlet-labelled bacteria (orange). Scale bars represent 10 µm. **g,** Comparative virulence factor profiles of the five *K. pneumoniae* strains. Data was derived from three biological replicates, each with at least 9 technical replicates (Supplementary Data 5,6).

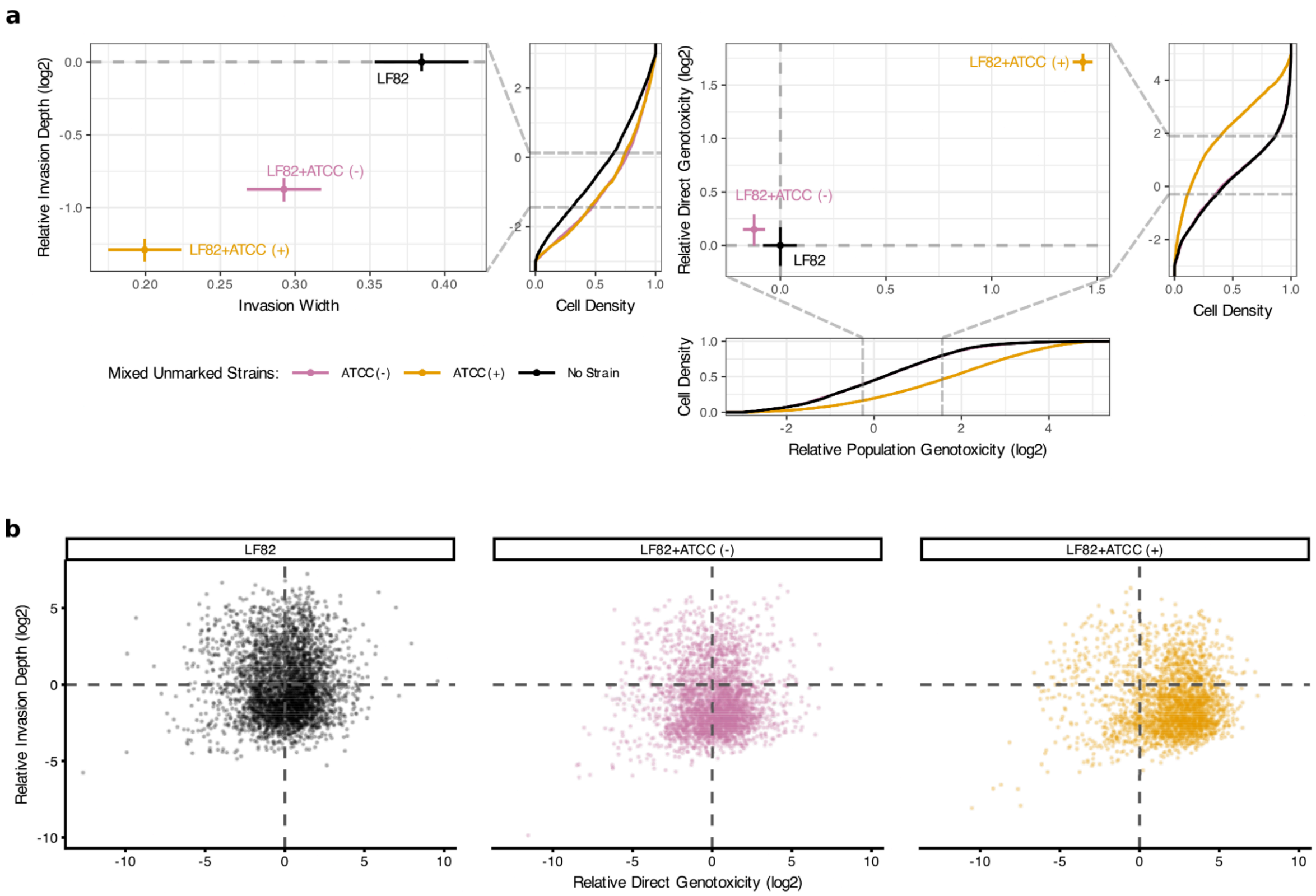

Supplementary Figure 4. Colibactin-dependent suppression of LF82 invasion. **a,** Invasion and genotoxicity-modulation landscape of fluorescently labelled LF82 strain during co-infection with the genotoxic ATCC(+) strain or its isogenic non-genotoxic mutant, ATCC(−). Invasion depth, population-level, and direct genotoxicity are calculated relative to the corresponding LF82 single-strain control, indicated by the black cross in each plot. Geometrically normalized cumulative density plots show the distribution of single-cell–derived metrics from labelled LF82–infected cells, either alone (black) or mixed with unlabelled ATCC(−) (pink) or unlabelled ATCC(+) (orange). **b,** Comparative scatter plot of direct genotoxicity versus invasion depth for single cells identified as labelled LF82–infected, either infected alone (black) or co-infected with unlabelled ATCC(−) (pink) or ATCC(+) (orange), shown relative to the mean of the labelled LF82 control. Each point represents a single cell measurement aggregated across replicates. Data was derived from two biological replicates, each with at least three technical replicates (Supplementary Data 7,8). Errors represent s.e.m.

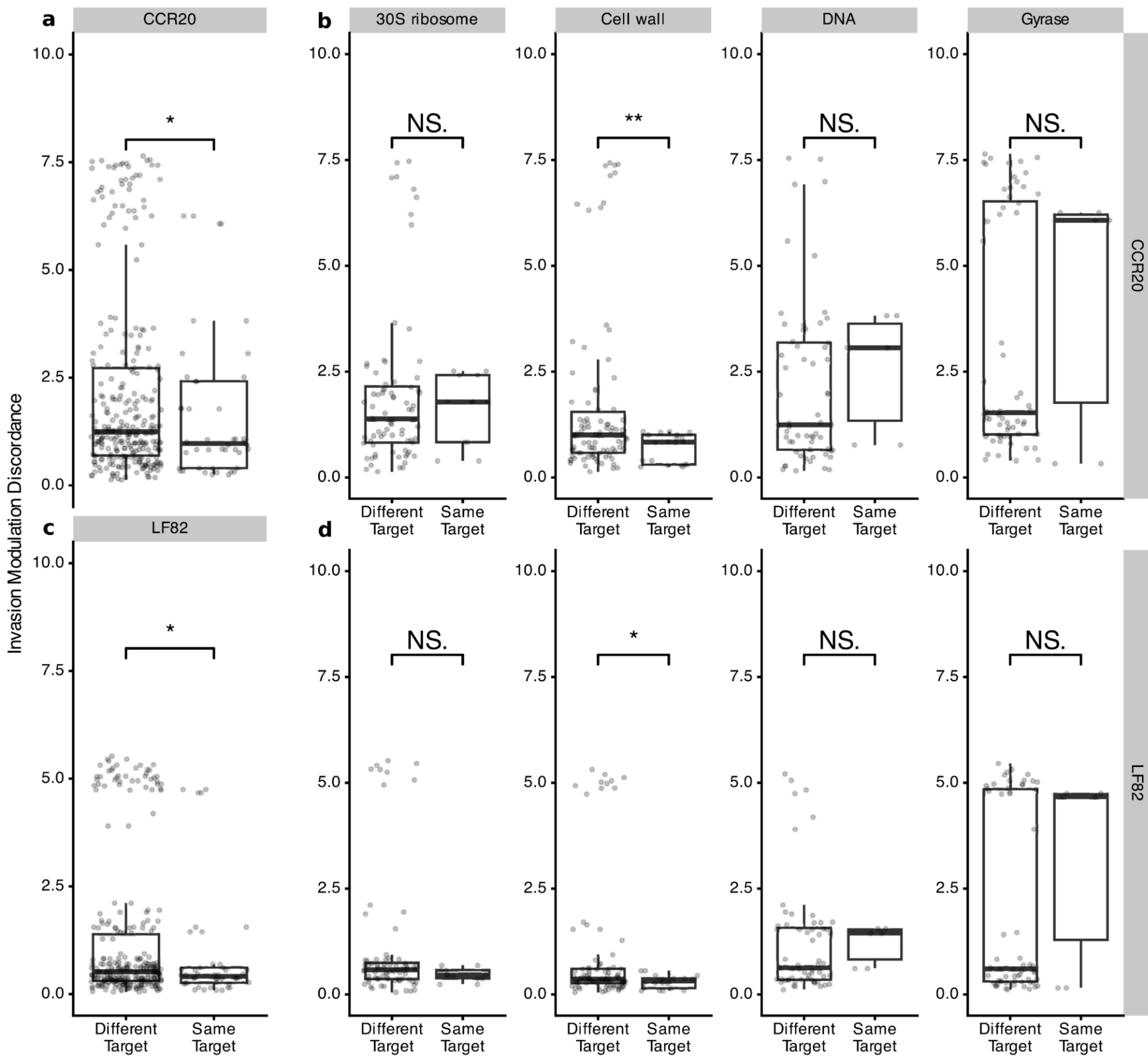

Supplementary Figure 5. Pairwise discordance of invasion modulation within and between drug target groups. Invasion width and depth were quantified for both doses of each drug, defining a four-dimensional invasion modulation space for each strain infection. Euclidean distances between drugs in this space were used to calculate invasion modulation discordance for each strain. Grouping discordance values by drug target allowed assessment of whether drugs acting on the same pathway modulate invasion consistently. For both CCR20 (**a–b**) and LF82 (**c–d**), drugs sharing the same target showed significantly lower modulation discordance compared to drugs with different targets (**a**, $p = 4.02 \times 10^{-2}$; **c**, $p = 3.52 \times 10^{-2}$). A similar trend was observed when comparing cell wall-targeting drugs to those with other targets (**b**, $p = 5.1 \times 10^{-3}$; **d**, $p = 2.13 \times 10^{-2}$, Wilcoxon test). Data was obtained from three biological replicates at two doses per drug. Boxplot hinges represent the first and third quantile, and the upper (lower) whisker extends to the largest (lowest) value no further than 1.5 times the distance between hinges.

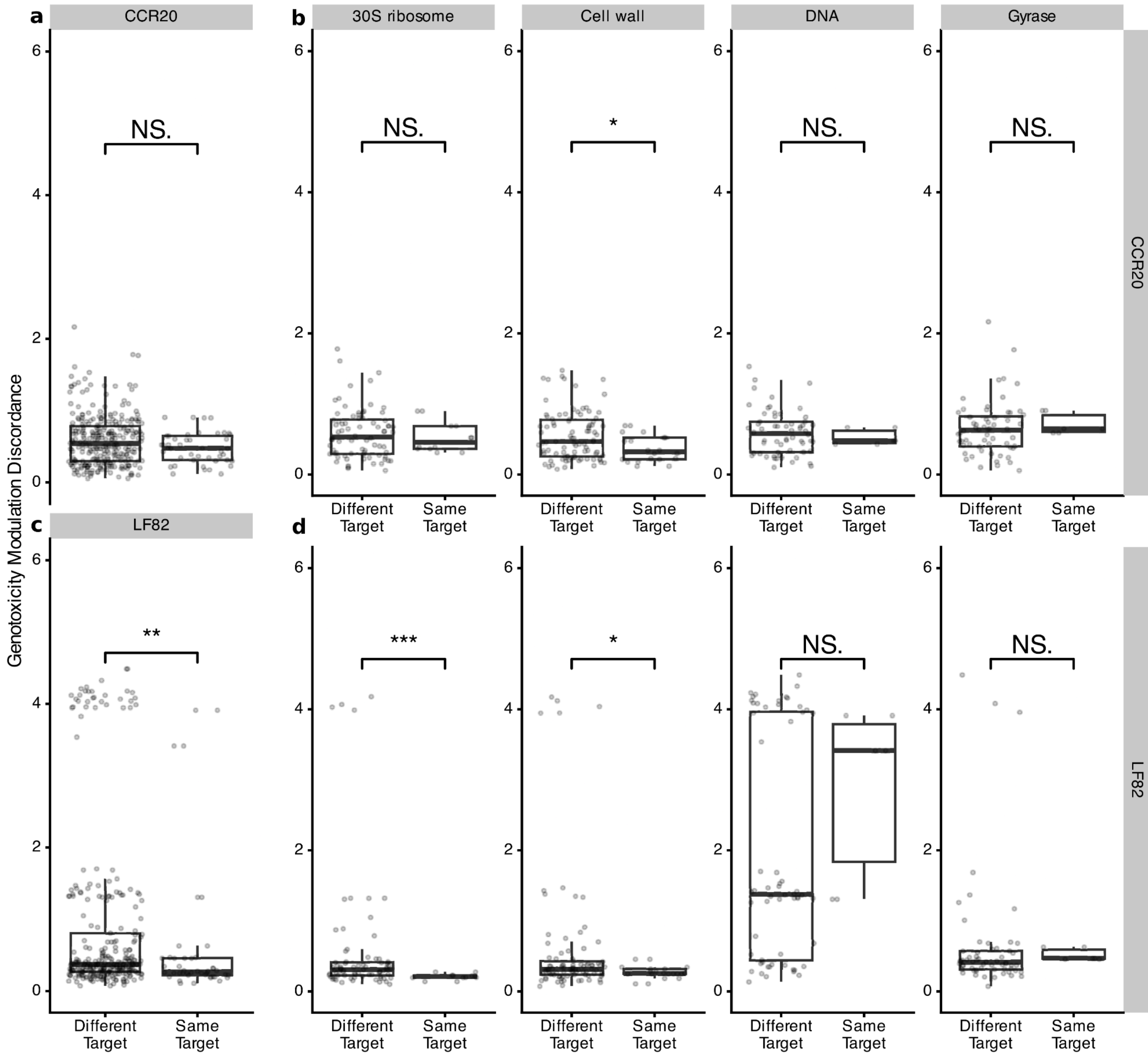

Supplementary Figure 6. Pairwise discordance of genotoxicity modulation within and between drug target groups. Population-level and direct genotoxicity effects were quantified for both doses of each drug, defining a four-dimensional genotoxicity modulation space. Euclidean distances between drugs in this space were used to compute genotoxicity modulation discordance for each strain (CCR20, **a–b**; LF82, **c–d**). Grouping discordance values by drug target enabled assessment of whether drugs acting on the same pathway modulate genotoxicity consistently. For LF82 infection (**c–d**), drugs sharing the same target showed significantly lower genotoxicity modulation discordance than those with different targets (**c**, $p = 2.50 \times 10^{-3}$). A similar trend was observed when comparing 30S ribosome-targeting drugs to those with other targets (**d**, $p = 7.372 \times 10^{-3}$). In both CCR20 (**a–b**) and LF82 (**c–d**) infections, cell wall-targeting drugs also showed significantly lower genotoxicity modulation discordance relative to drugs with other targets (**b**, $p = 1.768 \times 10^{-2}$; **d**, $p = 2.473 \times 10^{-2}$; Wilcoxon test). Data was obtained from three biological replicates at two doses per drug. Boxplot hinges represent the first and third quantile, and the upper (lower) whisker extends to the largest (lowest) value no further than 1.5 times the distance between hinges.

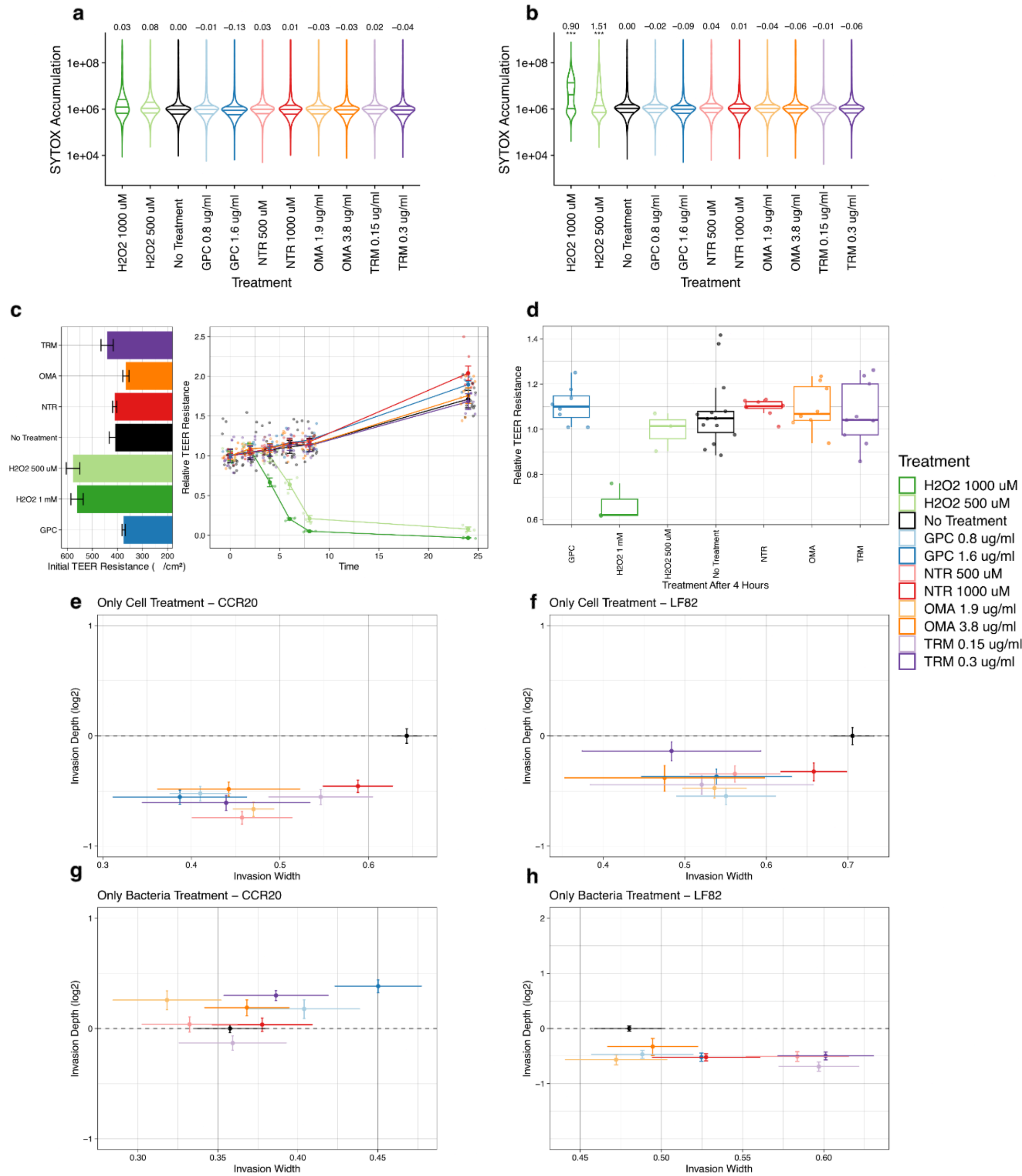

Supplementary Figure 7. Control experiments assessing host-cell toxicity, epithelial barrier integrity, and unidirectional drug pre-treatment effects on invasion phenotypes. a–b, SYTOX Blue accumulation in epithelial cells after 4 h (**a**) or 24 h (b) exposure to selected treatments, including H2O2 positive controls inducing membrane permeabilisation and epithelial barrier disruption, GPC, NTR, OMA, and TRM at the indicated concentrations. Values above violins indicate effect sizes relative to untreated controls (Glass's Δ). Large effects (Δ ≥ 0.8) are marked with three asterisks (***). Except for H2O2, treatments induced minimal changes in SYTOX accumulation (Δ < 0.1). Crossbars indicate mean and interquartile range. Data represent distributions of single-cell signals derived from six replicates across four independent experiments. **c**, Time-resolved transepithelial electrical resistance (TEER) measurements following highest-dose compound treatment. H2O2 caused a marked reduction in TEER, whereas the remaining treatments largely preserved epithelial barrier integrity over 24 h. **d**, Endpoint relative TEER values after 4 h treatment. Boxplots show replicate distributions for each condition. Treatments other than H2O2 remained within the range of untreated controls. Only the higher compound doses were tested in TEER experiments, with at least six replicates per condition. **e–f**, Invasion phenotypes following 4-hour host-cell-only pre-treatment and drug washout prior to infection with CCR20 (**e**) or LF82 (**f**). Data were obtained from three replicates across two independent experiments at two doses per drug (Supplementary Data 9). **g–h,** Invasion phenotypes following 1-hour bacteria-only pre-treatment and drug washout prior to infection with CCR20 (**g**) or LF82 (**h**). Points indicate treatment-level invasion phenotypes relative to untreated controls, with horizontal and vertical error bars representing standard errors of invasion width and invasion depth, respectively. The untreated condition is shown in black. Data were obtained from 10 replicates across two independent experiments at two doses per drug (Supplementary Data 10).

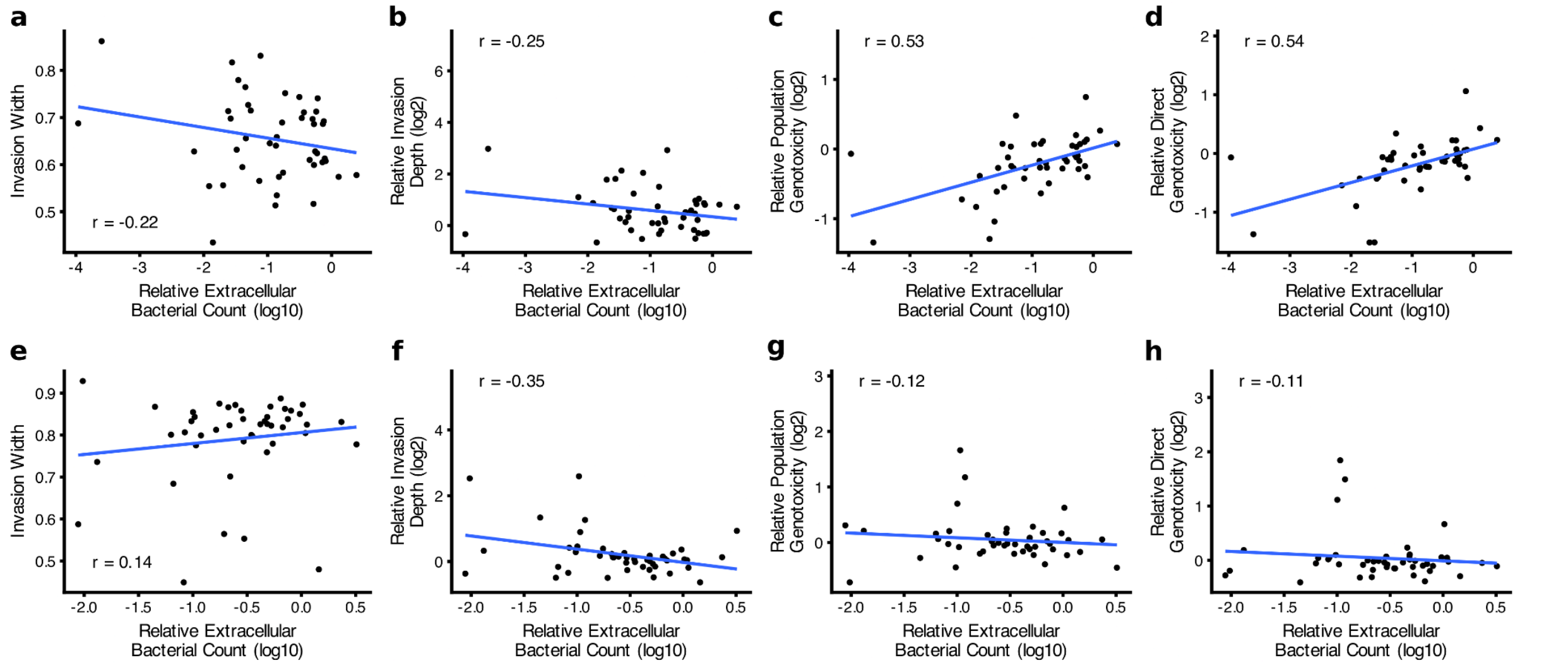

Supplementary Figure 8. Correlation of drug-driven bacterial clearance with invasion and genotoxicity modulation. To test whether antimicrobial effects on invasion and genotoxicity reflected direct virulence modulation rather than bacterial clearance, we examined correlations between extracellular bacterial abundance and each virulence metric, expressed relative to untreated controls. Pearson's correlation was calculated for invasion width (**a,** $p = 1.45*10^{-1}$; **e,** $p = 3.54*10^{-1}$), invasion depth (**b**, $p = 9.45*10^{-2}$**;** **f**, $p = 1.81*10^{-2}$), population genotoxicity (**c**, $p = 1.38*10^{-4}$**;** **g**, $p = 4.19*10^{-1}$), and direct genotoxicity (**d**, $p = 9.99*10^{-5}$**;** **h**, $p = 4.57*10^{-1}$). Analyses were performed separately for CCR20 (**a–d**) and LF82 (**e–h**) infections. Correlation coefficients (Pearson's *r*) are shown in the top or bottom left corner of each panel. Data was obtained from three biological replicates at two doses per drug.

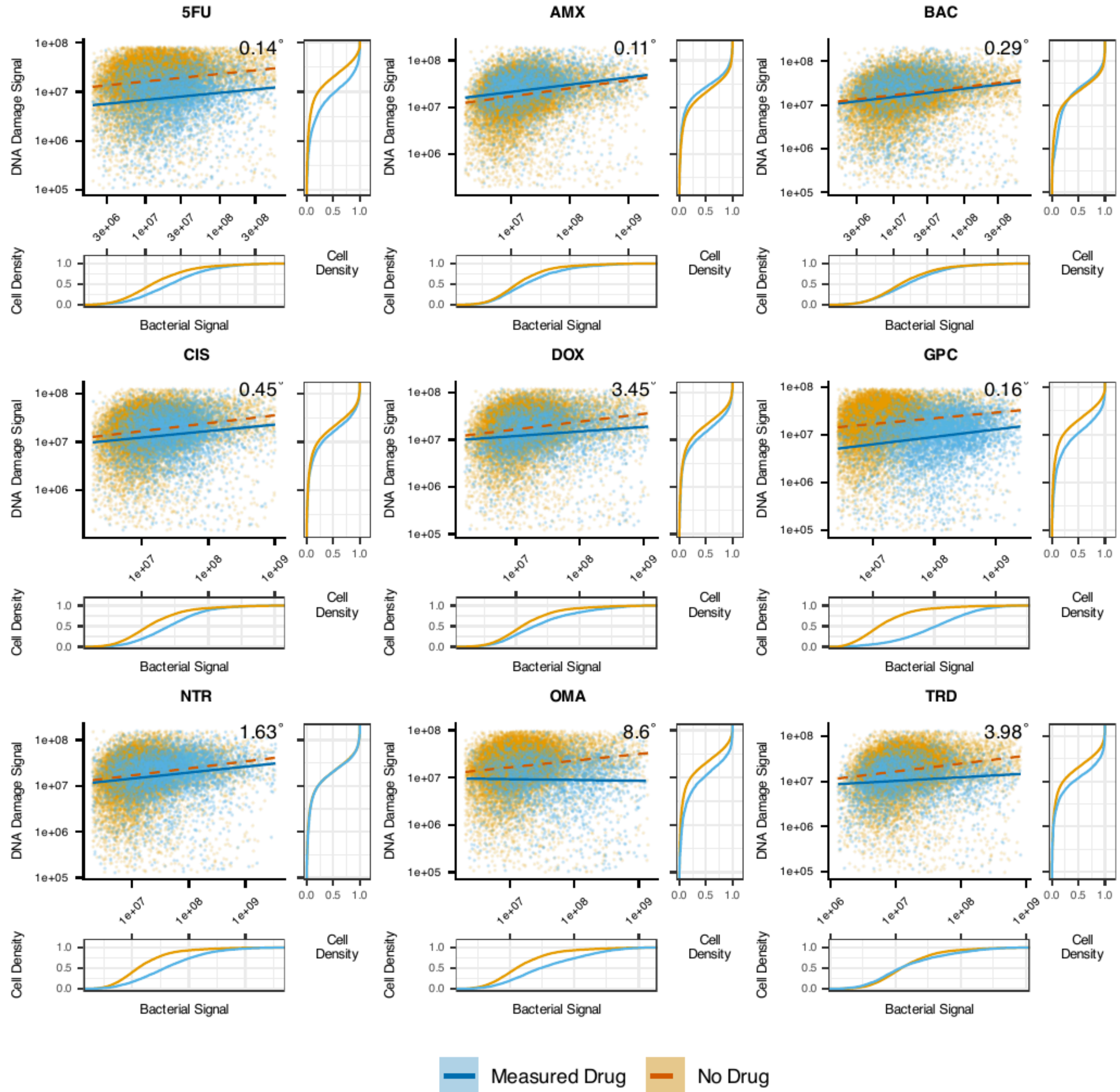

Supplementary Figure 9. Genotoxicity shifts in the presence of the CCR20 strain. Shifts are shown for drugs from different genotoxicity-modulation classes during CCR20 infection (see Figure 5d). Bacterial and DNA damage signals in infected human cells were plotted and fitted with a generalized linear model. The genotoxicity shift angle (top right of each plot) is defined as the absolute change in the slope angle of the log–log relationship between intracellular bacterial load and DNA damage, measured between untreated (orange) and drug-treated (blue) conditions. Data was obtained from three biological replicates at two doses per drug.

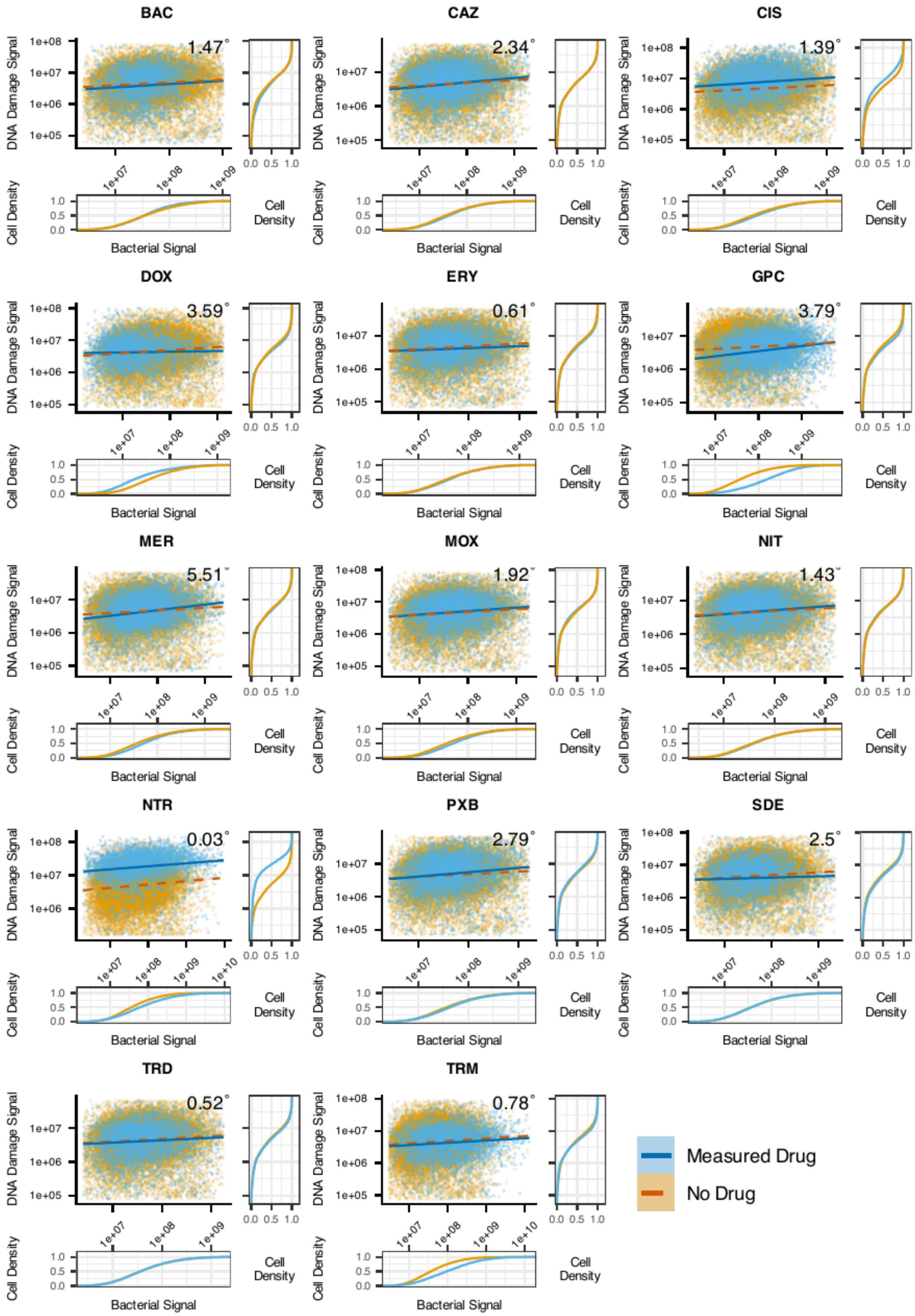

Supplementary Figure 10. Genotoxicity shifts in the presence of the LF82 strain. Shifts are shown for drugs from different genotoxicity-modulation classes during LF82 infection (see Figure 5c). Bacterial and DNA damage signals in infected human cells were plotted and fitted with a generalized linear model. The genotoxicity shift angle (top right of each plot) is defined as the absolute change in the slope angle of the log-log relationship between intracellular bacterial load and DNA damage, measured between untreated (orange) and drug-treated (blue) conditions.

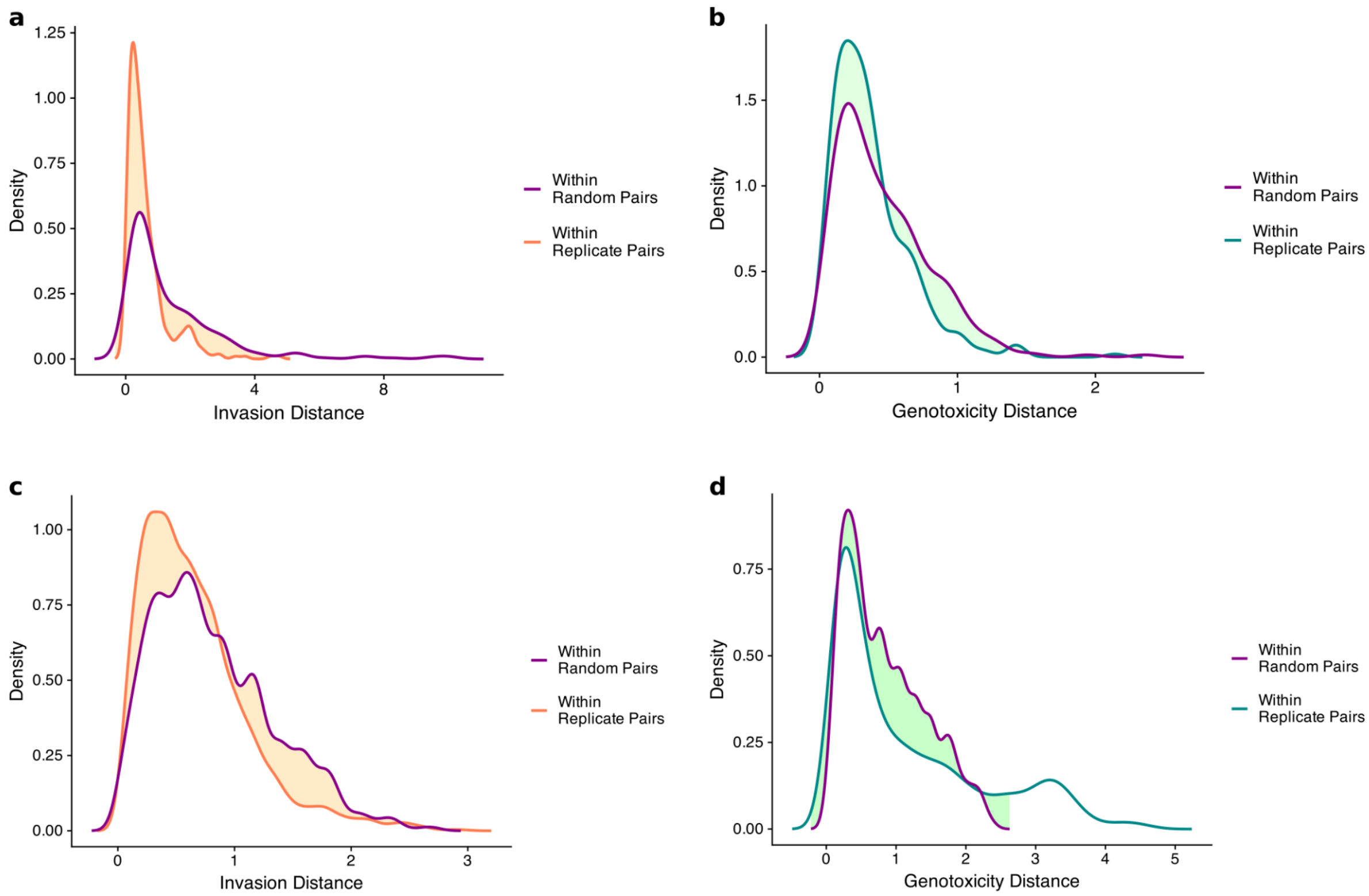

Supplementary Figure 11. Comparison of virulence metric distances between replicate and random pairs. To determine whether the virulence metrics captured true biological variation rather than noise, we compared pairwise Euclidean distances between experimental replicates and randomly selected data points from the same experiments. Distances were computed separately in two metric spaces: the invasion plane (**a**,**c**; invasion width and depth) and the genotoxicity plane (**b,d**; direct and population-level genotoxicity). Distributions of distances for true replicate pairs and random pairs were compared for drug screens (Kolmogorov–Smirnov test: **a**, D = 0.28, $p = 4.7 \times 10^{-12}$; **b**, D = 0.15, $p = 1.3 \times 10^{-3}$) and for strain and intra-strain interaction characterisations (Kolmogorov–Smirnov test: **c**, D = 0.16, $p = 7.8 \times 10^{-82}$; **d**, D = 0.18, $p = 2.2 \times 10^{-105}$). Orange and green curves represent distances between replicate pairs, while purple curves represent distances between random pairs.

Compound	Short name	1x dose	2x dose	Type	Drug class	Target
Omadacycline	OMA	1.9 μg/mL	3.8 μg/mL	AB	Tetracycline	30S ribosome
Doxycycline hydrochloride	DOX	6.4 μg/mL	12.8 μg/mL	AB	Tetracycline	30S ribosome
Zoliflodacin	ZOL	1 μg/mL	2 μg/mL	AB	Hydroquinoline	Gyrase
Moxifloxacin	MOX	0.03 μg/mL	0.05 μg/mL	AB	Fluoroquinolone	Gyrase, topo IV
Benzalkonium chloride	BAC	3.9 μg/mL	7.8 μg/mL	BI	QAC[a] salt	Inner membrane
Nitrofurantoin	NIT	6.4 μg/mL	12.8 μg/mL	AB	Nitrofuran	Multiple
Metronidazole	MET	50 μg/mL	100 μg/mL	AB	Nitroimidazole	DNA
Apramycin sulfate	APS	12.8 μg/mL	25.6 μg/mL	AB	Aminoglycoside	30S ribosome
Amikacin	AMI	32 μg/mL	64 μg/mL	AB	Aminoglycoside	30S ribosome
Amoxicillin	AMX	500 μg/mL	1000 μg/mL	AB	Aminopenicillin	Cell wall
5-fluorouracil	5FU	50 μM	100 μM	CT	Nucleoside antimetabolite	Thymidylate synthase
Aztreonam	AZT	0.1 μg/mL	0.2 μg/mL	AB	Monobactam	Cell wall
Meropenem	MER	0.005 μg/mL	0.009 μg/mL	AB	Carbapenem	Cell wall
Ceftazidime	CAZ	0.05 μg/mL	0.10 μg/mL	AB	Cephalosporin	Cell wall
Gepotidacin	GPC	0.8 μg/mL	1.6 μg/mL	AB	Triazaacenaphthylene	Gyrase, topo IV
Sodium sulfite	SDE	500 μg/mL	1000 μg/mL	BI	Inorganic salt	Multiple
Tridecaptin M152-P3	TRD	3 μg/mL	6 μg/mL	AB	Cationic lipopeptides	Lipid II
Triclosan	TCS	0.011 μg/mL	0.022 μg/mL	BI	Phenoxyphenol	Fatty acid synthesis
Erythromycin	ERY	25 μg/mL	50 μg/mL	AB	Macrolide	Protein synthesis
Polymyxin B	PXB	0.4 μg/mL	0.8 μg/mL	AB	Polymyxin	Cell wall
Trimethoprim	TRM	0.15 μg/mL	0.3 μg/mL	AB	Antifolate antibiotic	Dihydrofolate reductase
Cisplatin [b]	CIS	200 μM	400 μM	CT	Platinum compound	DNA
n-Nitroso-n-Ethylurea	NTR	500 μM	1000 μM	CT	DNA Alkylating agent	DNA

[a] Quaternary ammonium compound, less formerly known as Quat
[b] Formerly: Peyrone's salt, classified as a platinum salt

Supplementary Table 1. A list of the drugs and their chosen concentrations used in the study (1x and 2x; Figure 5,6,7 and Methods). AB: antibiotic, BI: biocid, CT: chemotherapeutic
