# Supplementary material for: Deep-learning single-cell profiling uncovers interbacterial and drug-driven reshaping of pathogen virulence
Source: Nat Commun. 2026 Sep 29;17:11236. doi: 10.1038/s41467-026-77989-w (PMC13720132; doi:10.1038/s41467-026-77989-w)
Supplement: Supplementary file 4 [file 41467_2026_77989_MOESM2_ESM.pdf]

Title: Supplementary Dataset 1: Replicates of inter-strain modulation of invasion measurements (Figure 3).

Description: This interactive file presents the technical and biological replicates used in the analysis.

Title: Supplementary Data 2: Replicates of inter-strain modulation of genotoxicity measurements (Figure 4).

Description: This interactive file presents the technical and biological replicates used in the analysis.

Title: Supplementary Dataset 3: Replicates of LF82 and CCR20 drug-induced bacterial invasion modulation measurements (Figure 5).

Description: This interactive file presents the technical and biological replicates used in the analysis.

Title: Supplementary Dataset 4: Replicates of LF82 and CCR20 drug-induced bacterial genotoxicity modulation measurements (Figure 6).

Description: This interactive file presents the technical and biological replicates used in the analysis.

Title: Supplementary Dataset 5: Replicates of K. pneumoniae measurements on A549 lung cells (Invasion).

Description: This interactive file presents the technical and biological replicates used in the analysis.

Title: Supplementary Dataset 6: Replicates of K. pneumoniae measurements on A549 lung cells (Genotoxicity).

Description: This interactive file presents the technical and biological replicates used in the analysis.

Title: Supplementary Dataset 7: Replicates of LF82 and ATCC co-infection measurements (Extended Data Figure 3) on CaCo intestinal epithelial cells (Invasion).

Description: This interactive file presents the technical and biological replicates used in the analysis.

Title: Supplementary Dataset 8: Replicates of LF82 and ATCC co-infection measurements (Extended Data Figure 3) on CaCo intestinal epithelial cells (Genotoxicity).

Description: This interactive file presents the technical and biological replicates used in the analysis.

Title: Supplementary Dataset 9: Replicates of LF82 and CCR20 only human cell treatment experiments (Extended Data Figure 7e,f).

Description: This interactive file presents the technical and biological replicates used in the analysis.

Title: Supplementary Dataset 10: Replicates of LF82 and CCR20 drug-induced bacterial invasion modulation measurements (Extended Data Figure 7g,h).

Description: This interactive file presents the technical and biological replicates used in the analysis.

Title: Supplementary Dataset 11: Model Analytics

Description: This file contains the validation accuracies and error metrics of the models used in this study.
