# Supplementary material for: Deep-learning single-cell profiling uncovers interbacterial and drug-driven reshaping of pathogen virulence
Source: Nat Commun. 2026 Sep 29;17:11236. doi: 10.1038/s41467-026-77989-w (PMC13720132; doi:10.1038/s41467-026-77989-w)
Supplement: Supplementary file 6 [file 41467_2026_77989_MOESM11_ESM.zip › Supplementary Data 9.html]

LF82, CCR20 Host-Treated Invasion


**Supplementary Data 9: Replicates of LF82 and CCR20 only human cell treatment experiments (Extended Data Figure 7e,f).** This interactive plot presents the technical and biological replicates used in the analysis. The *Replicate* column shows biological replicates. Each datapoint is presented per well. Scale bar represents cellular signal variance calculated as standard error. The Treatments are separated by color, and clicking on a treatment label in the top section can be used to hide/show the treatment in question.
