# Supplementary material for: Deep-learning single-cell profiling uncovers interbacterial and drug-driven reshaping of pathogen virulence
Source: Nat Commun. 2026 Sep 29;17:11236. doi: 10.1038/s41467-026-77989-w (PMC13720132; doi:10.1038/s41467-026-77989-w)
Supplement: Supplementary file 8 [file 41467_2026_77989_MOESM3_ESM.zip › Supplementary Data 1.html]

Inter-strain modulation of invasion


**Supplementary Data 1: Replicates of inter-strain modulation of invasion measurements (Figure 3).** This interactive plot presents the technical and biological replicates used in the analysis. The *Replicate* column shows biological replicates and the Marked Strains column shows the fluorescently labelled strain of interest, both of which can be filtered freely. Each datapoint is presented per well. Scale bar represents cellular signal variance calculated as standard error. The unmarked, modulating strains are separated by color, and clicking on a strain lable in the top section can be used to hide/show the strain in question.
