# Supplementary material for: Deep-learning single-cell profiling uncovers interbacterial and drug-driven reshaping of pathogen virulence
Source: Nat Commun. 2026 Sep 29;17:11236. doi: 10.1038/s41467-026-77989-w (PMC13720132; doi:10.1038/s41467-026-77989-w)
Supplement: Supplementary file 11 [file 41467_2026_77989_MOESM6_ESM.zip › Supplementary Data 4.html]

LF82, CCR20 Drug-Modulated Genotoxicity


**Supplementary Data 4: Replicates of LF82 and CCR20 drug-induced bacterial genotoxicity modulation measurements (Figure 6).** This interactive plot presents the technical and biological replicates used in the analysis. The *Replicate* column shows biological replicates of experiment *Sets* 1 and 2, where both can be filtered freely. Each datapoint is presented per well. Scale bar represents cellular signal variance calculated as standard error. The Treatments are separated by color, and clicking on a treatment label in the top section can be used to hide/show the treatment in question.
