# Supplementary material for: Deep-learning single-cell profiling uncovers interbacterial and drug-driven reshaping of pathogen virulence
Source: Nat Commun. 2026 Sep 29;17:11236. doi: 10.1038/s41467-026-77989-w (PMC13720132; doi:10.1038/s41467-026-77989-w)
Supplement: Supplementary file 13 [file 41467_2026_77989_MOESM8_ESM.zip › Supplementary Data 6.html]

K. pneumoniae Genotoxicity


**Supplementary Data 6: Replicates of** ***K. pneumoniae*** **measurements on A549 lung cells (Genotoxicity).** This interactive plot presents the technical and biological replicates used in the analysis. The *Replicate* column shows biological replicates, which can be filtered freely. Each datapoint is presented per well. Scale bar represents cellular signal variance calculated as standard error. The Strains are separated by color, and clicking on a strain lable in the top section can be used to hide/show the strain in question.
